# Two birds with one stone: SGI1 can stabilize itself and expel the IncC helper by hijacking the plasmid *parABS* system

**DOI:** 10.1093/nar/gkae050

**Published:** 2024-02-01

**Authors:** Gábor Murányi, Mónika Szabó, Károly Acsai, János Kiss

**Affiliations:** Department of Microbiology and Applied Biotechnology, Institute of Genetics and Biotechnology, Hungarian University of Agriculture and Life Sciences, Gödöllő, H2100 Hungary; Department of Microbiology and Applied Biotechnology, Institute of Genetics and Biotechnology, Hungarian University of Agriculture and Life Sciences, Gödöllő, H2100 Hungary; Agribiotechnology and Precision Breeding for Food Security National Laboratory, Gödöllő, H2100 Hungary; Ceva Animal Health, Ceva-Phylaxia, Budapest, H1107 Hungary; Department of Microbiology and Applied Biotechnology, Institute of Genetics and Biotechnology, Hungarian University of Agriculture and Life Sciences, Gödöllő, H2100 Hungary; Agribiotechnology and Precision Breeding for Food Security National Laboratory, Gödöllő, H2100 Hungary

## Abstract

The SGI1 family integrative mobilizable elements, which are efficient agents in distribution of multidrug resistance in Gammaproteobacteria, have a complex, parasitic relationship with their IncC conjugative helper plasmids. Besides exploiting the transfer apparatus, SGI1 also hijacks IncC plasmid control mechanisms to time its own excision, replication and expression of self-encoded T4SS components, which provides advantages for SGI1 over its helpers in conjugal transfer and stable maintenance. Furthermore, SGI1 destabilizes its helpers in an unknown, replication-dependent way when they are concomitantly present in the same host. Here we report how SGI1 exploits the helper plasmid partitioning system to displace the plasmid and simultaneously increase its own stability. We show that SGI1 carries two copies of sequences mimicking the *parS* sites of IncC plasmids. These *parS*-like elements bind the ParB protein encoded by the plasmid and increase SGI1 stability by utilizing the *parABS* system of the plasmid for its own partitioning, through which SGI1 also destabilizes the helper plasmid. Furthermore, SGI1 expresses a small protein, Sci, which significantly strengthens this plasmid-destabilizing effect, as well as SGI1 maintenance. The plasmid-induced replication of SGI1 results in an increased copy-number of *parS*-like sequences and Sci expression leading to strong incompatibility with the helper plasmid.

## Graphical Abstract

**Graphical Abstract:**
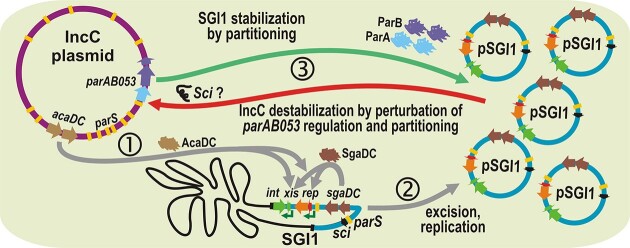

## Introduction

Multidrug-resistant (MDR) pathogenic bacteria represent a global threat to public health and animal husbandry. The current practice of preventive overuse, or even misuse, of antibiotics in livestock farming could lead to a post-antibiotic era when drugs may be ineffective for treatment of bacterial infections (1). The emergence of MDR bacteria is mainly facilitated by the activity of various mobile genetic elements such as transposons, genomic islands and conjugative plasmids, which often carry antibiotic resistance (AR) genes. Mobile genomic islands, also referred to as integrative elements (IE), have recently been recognized as key players in multidrug resistance propagation (2,3) as they generally contain arrays of resistance genes associated with, or independently of, integrons. Two major types of IE have been distinguished based on their autonomous functions. Integrative and conjugative elements (ICEs, formerly known as conjugative transposons (4)) possess the entire genetic apparatus required for chromosomal integration, excision, and conjugative transfer. Unlike ICEs, integrative mobilizable elements (IMEs) can integrate autonomously, but lack a complete set of conjugation genes, and therefore require a transfer-competent helper element, such as a conjugative plasmid or ICE, to ensure their horizontal transfer (5).

To date, almost 260 IMEs have been identified (6) in a wide range of Gram− and Gram+ bacteria. One of the largest IME families encompasses variants and more distant relatives of *Salmonella* Genomic Island 1 (SGI1). The prototype SGI1 element was first identified in a multiresistant pandemic clone of *Salmonella* serovar Typhimurium DT104 (7) in the early 1980s, became prevalent in the 1990s (8), and now SGI1 variants and related elements are the main causative agents of multi-resistance in many human pathogens, such as *Salmonella enterica* serovars, *Proteus mirabilis*, *Morganella morganii*, *Acinetobacter baumannii*, *Providencia stuartii*, *Enterobacter* spp., *Escherichia coli* and *Klebsiella pneumoniae* strains (9–15).

**Figure 1.**
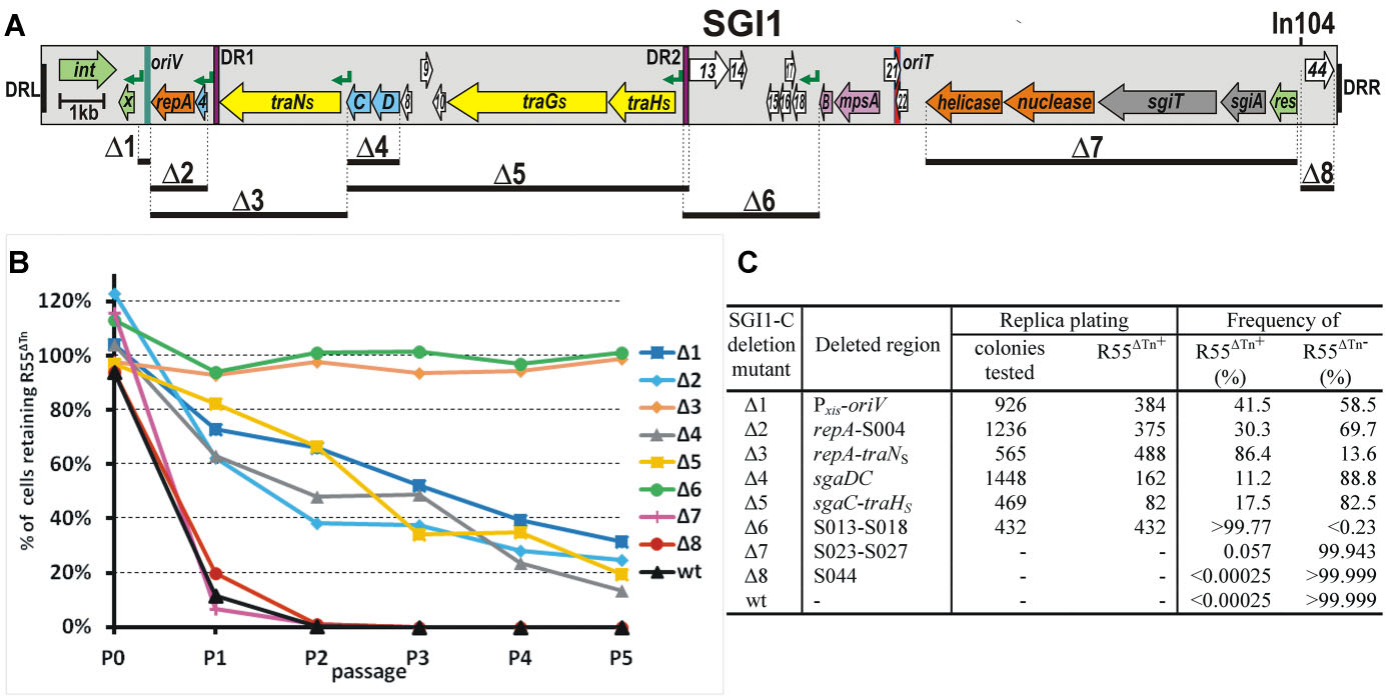
Incompatibility of wt SGI1-C and its deletion mutants with the IncC plasmid R55^ΔTn^. (**A**) Schematic map of the conserved SGI1 backbone. Annotated ORFs S001-S044 are indicated by colour-coded arrows: green – recombinase; orange – replication; blue – transcription regulator; yellow – T4SS components; purple – conjugation initiation, grey – TA system; white – unknown function. Names of genes with known functions or identified homologs are indicated. Abbreviations: *x* – *xis*, *C* and *D* – *flhC*_SGI1_ and *flhD*_SGI1_, *sgiT* and *sgiA* – toxin and antitoxin genes of the TA system, *B* – *mpsB*. Genes of unknown functions are numbered according to the original numbering (e.g. ‘8′ refers to S008, etc.). Terminal direct repeats are shown as black boxes, green and red vertical lines indicate *oriV* and *oriT*, respectively. Purple vertical lines indicate the 31-bp imperfect direct repeats (DR1 and DR2), and green broken arrows represent AcaCD/SgaDC-responsive promoters. Positions of In104 (carrying the AR genes *aadA2*, *floR*, *tetG*, *pse-1*, *sul1* – in the case of protoype SGI1) and eight deletions (Δ1–Δ8) generated in SGI1-backbone are indicated (the map is drawn to scale). (**B**) Dynamics of R55^ΔTn^ loss from TG1Nal strains harbouring wt or one of the Δ1–Δ8 deletion mutant SGI1-C. Graph shows the percentage of R55^ΔTn+^ cells grown under selection for SGI1 but not for R55^ΔTn^ through 5 passages. Each passage represents approx. 20 generations. The % values are means of four biological replicates (for the individual segregation curves with standard deviations, see Supplementary Figure S1). **(C)** Proportion of R55^ΔTn+^ cells after the 5th passage (100 generations growth). Four replicates from the 5th passage were pooled, spread on LB plates selective for SGI1, and retention of R55^ΔTn^ was individually tested by replica plating. Number of total and R55^ΔTn+^ colonies tested are listed and the plasmid loss is indicated as % of the R55^ΔTn−^ colonies. If no R55^ΔTn−^ colonies were found, the threshold limit was calculated as 1/total colony count. If high-frequency loss of R55^ΔTn^ was observed, the percentage of R55^ΔTn+^ cells was calculated as mean of the Cm^R^/total cell titers of the four parallels measured in the 5th passage.

The prototype SGI1 and most of its variants (16,17) share a conserved backbone interrupted by the In104 cluster, which harbours AR genes (Figure 1A). The backbone includes: an integration/excision (*int* and *xis*) module; a replication module, consisting of *oriV*, and genes encoding a leader peptide (S004) and *repA* (18); genes related to plasmid-borne T4SS genes (*traN_S_, traG_S_, traH_S_*) (19,20); an operon encoding FlhDC-family regulators (21); a mobilization module, including the transfer origin (*oriT*) and genes encoding two mobilization proteins MpsA and MpsB, and a small RNA, sgm-sRNA (22,23); a helicase and a nuclease gene (19); a toxin–antitoxin (TA) system (24); a resolvase gene; and several further annotated open reading frames (ORFs) with unknown functions (*S008-S010, S013-S018, S044*) (19). The In4-type In104 integron cluster of SGI1 comprises two integrons containing AR genes, IS elements (IS*CR3*, IS*6100*), and some additional genes of unknown function (19).

SGI1-family elements are mobilized by the broad host range, low-copy IncA and IncC conjugative plasmids (25–27). These closely-related plasmids (27,28) have the same conjugative system as the MOB_H12_ group (29). The conjugation genes are regulated by the FlhDC-family master activator, AcaCD, whose expression is controlled by negative and positive feedback loops established by the Acr1 and Acr2 repressors and the transcription activator, AcaB (30–32). SGI1 does not simply utilize this conjugation apparatus, but applies several refined interventions at the expense of the plasmid, to increase the success of its own lateral transfer and ensure stable maintenance in donor and recipient cells (20,18,33).

After entry into the new host, SGI1 integrates into the chromosome at the 3′ end of *trmE* (also known as *thdF* or *mnmE*) via site-specific recombination carried out by the SGI1 λ-family integrase (Int) (25). This ensures highly stable vertical transfer of SGI1 (34,24) until an IncC plasmid appears. When the plasmid is present, five SGI1 operons, including *xis*, *rep*, *traN_S_*, and *traH_S_G_S_* (Figure 1A), that are controlled by AcaCD-responsive promoters (35), are activated, allowing SGI1 to exploit several functions of the plasmid. AcaCD-activation induces production of the recombination directionality factor, Xis, which promotes SGI1 excision catalysed by Int (30,21). This recombination event generates the extrachromosomal circular form of SGI1, which can be mobilized by the helper plasmid, but is also liable to segregation (21,24,36,18). To prevent SGI1 loss from cells in which it coexists with the helper plasmid, the island both expresses a TA system (24) and begins to replicate like a plasmid. Replication initiated by an IncN2-type RepA protein expressed from the *S004*-*rep* operon, which is also activated by AcaCD, maintains excised SGI1 at 6–8 copies (36,18), significantly increasing its stability (18); however, another FlhDC-family activator, the SGI1-encoded SgaDC (also known as FlhDC_SGI1_) is also necessary to reach the optimal SGI1 copy number. In the absence of SgaDC, AcaCD-activation of *S004*-*rep* is reported to maintain SGI1 in an approximately single-copy state (18); however, complete loss of the SGI1^Kn^Δ*sgaDC* replication has also been reported (37).

Many observations suggest that coexistence of SGI1 and IncC plasmids is not stable. Natural isolates harbouring both elements have never been reported. Under experimental circumstances, a moderate frequency of SGI1 loss was observed in *Salmonella* Typhimurium strains when antibiotic selection was applied only for the IncC plasmid, R55 (21). SGI1 loss was also observed in *E. coli* under nonselective conditions; however, SGI1 appears to be more stable than the IncC plasmid, which is lost from 45%-85% of the cell population after 20 generations (38,36,18,39). Since SGI1 behaves like a plasmid in the presence of an IncC plasmid, their mutual destabilization resembles the phenomenon of plasmid incompatibility, which is defined as the failure of stable inheritance of co-resident plasmids without external selection. This phenomenon occurs if a plasmid destabilizes another within the same host cell because their maintenance functions, such as elements involved in replication and copy number control, or partitioning, interfere with one another due to their relatedness, and leading to irregular plasmid inheritance. Incompatibility is symmetric when both plasmids are lost with equal probability, or vectorial when one plasmid is lost with higher probability (40).

Replication-mediated incompatibility is a well-established model that has led to the paradigm of classifying plasmids into widely used incompatibility groups (Inc). In this case, the sharing of related elements (iterons, antisense RNAs, RepA proteins) that control the replication of both plasmids can lead to a complete or partial blocking of replication of one plasmid, or prevent the replication of both plasmids at the frequency required for stability (40). For stable maintenance, low-copy number plasmids need active partitioning by Par systems, which ensure that every daughter cell receives at least one plasmid copy after segregation (41). Par systems are also important for ICE stability (42–44). The systems responsible for partitioning are also significant incompatibility factors. Similarly to replication-mediated incompatibility, competition arises between two plasmids or ICEs for shared components if they have closely related Par systems, which can lead to partition-mediated incompatibility (41,45). Generally, partition systems comprise three main components: one or more copies of a partition site (‘centromere’), a centromere binding protein (CBP), and an ATPase or GTPase protein. Three general system types have been identified and classified based on their partition NTPases (46,45). The most prevalent Type I systems (*parABS*), which occur in many plasmids, such as F, P1 or RK2, and are the only Par systems of bacterial chromosomes, include a Walker-type ATPase (ParA), a centromere binding protein (CBP or ParB), and one or more centromere-like DNA sites, *parS*. Interestingly, a number of plasmids have two Par systems, generally of different types (e.g. pB171, R27). In these cases, Type I systems appear to be more important for plasmid stabilization (47,48). In addition, TA system-mediated incompatibility has also been reported among Ti plasmids (49).

Such systems may be associated with the incompatibility between SGI1 and the IncA and IncC plasmids, although no Par system has yet been identified in SGI1. Replication of IncC plasmids and SGI1 are driven by the unrelated IncC (50) and IncN2 (18) replicons, respectively. The TA systems of each element are also unrelated; the conserved IncC backbone contains a HigBA-like RelE/ParE family Type II TA-system (30,51,52), while the SGI1 *sgiAT* operon encodes an S8 family subtilisin-like serine protease toxin (24) and an AAA+-type ATPase-like antitoxin related to the SpoVK/Ycf46/Vps4 superfamily of NTPases.

The third major factor that often mediates incompatibility is partitioning. IncC plasmids belong to a class of plasmids that have two Par systems, as both *parABS* and *parMRC* loci have been identified in their backbone. The Type I *parABS* system is crucial for plasmid maintenance, as no insertions were obtained in the *parA* or *parB* genes in a Tn*5* mutagenesis screen, indicating that these genes are essential. A further indispensable factor, *ORF053*, was also detected (51), however, its exact role and identification of the cognate *parS* site(s) of the system remain to be established. In contrast, the *parMRC* system, which appears to be part of the AcaCD-regulon (30), may be less important for IncC stability, as this locus was not identified as non-mutable (51). Interestingly, this Par system is related to *srpMRC* identified in SXT/R391 ICEs, which is expressed under the control of the FlhDC-family activator, SetCD, and involved in reducing R391 loss (44). Unlike SXT-family ICEs, SGI1 appears to lack a functional Par system. Thus, apparently neither replication-mediated, nor TA- or partition-mediated incompatibility plays a role in the SGI1-IncC relationship.

Recent works have indicated that the SGI1-encoded regulator, SgaDC, which has a key role in crosstalk between SGI1 and IncC plasmids, may also be involved in their incompatibility. The SGI1-K variant, lacking the *sgaDC* operon due to a deletion did not destabilize the IncC plasmid pRMH760 (38,39). Further support for this hypothesis came from stability assays, where the IncC plasmid, R55, appeared to be much more stable under nonselective conditions in the presence of an SGI1 mutant lacking *sgaDC* than in the presence of a wt SGI1-C (18). Since SgaDC is proven to be a key factor in SGI1 replication, a consensus has emerged that it acts on SGI1-IncC incompatibility by influencing the control of SGI1 replication and copy number (18,37), however, the exact mechanism remains unclear. Alhough SgaDC appears to have a key role in SGI1-IncC incompatibility, it has been previously suggested that the ultimate explanation for this phenomenon may rather be the presence of an, as yet unidentified, SGI1-encoded factor, whose dosage is increased due to SGI1 replication in the presence of the helper (18). In this study, we report discorvery of such factors and describe how SGI1 destabilizes the IncC plasmid and increases its own stability by disturbing and hijacking the main partition system of the plasmid using these factors.

## Materials and methods

### DNA and microbial techniques

Standard molecular biology procedures were carried out according to (53). Enzymes were purchased from Thermo Fisher Scientific, New England Biolabs and Merck; chemicals were from Merck, Roth and Reanal. For cloning purposes, *E. coli* TG1 was used as the host strain. Pwo (Roche) or Phusion (Thermo Fisher Scientific) high-fidelity DNA polymerases were applied for PCR and cloned PCR products were sequenced on an ABI 3500xL Genetic Analyzer (Life Technologies). Oligonucleotides used in this work are listed in Supplementary Table S1. Primers were designed using the published sequence of SGI1 (GenBank: AF261825). Test/colony PCRs were performed using DreamTaq polymerase (Thermo Fisher Scientific), as described previously (34). Relevant features of plasmids are listed in Supplementary Table S2. Detailed methodology of construction of plasmids is described in Text S1.

Total DNA for RT-qPCR assays was isolated using ExctractMe DNA & RNA Extraction kit (Blirt S.A.), according to the manufacturer's protocol.

Bacterial strains listed in Supplementary Table S3 were grown at 37°C in LB broth/agar supplemented with the appropriate antibiotics at the following final concentrations: ampicillin (Ap) 150 μg/ml, chloramphenicol (Cm) 20 μg/ml, kanamycin (Km) 30 μg/ml, spectinomycin (Sp) 50 μg/ml, streptomycin (Sm) 50 μg/ml, nalidixic acid (Nal) 20 μg/ml, and tetracycline (Tc) 10 μg/ml.

Mating was carried out as described previously (23). Briefly, 100 μl of overnight (ON) cultures (1–2 × 10^9^ cells/ml) of donor and recipient strains grown in LB supplemented with appropriate antibiotics were mixed, centrifuged for 1 min, washed with 0.5 ml 0.9% NaCl, spread on LB agar plates, and incubated for 6 h at 37°C. After suspension and dilution of the bacterial lawn in 0.5 ml 0.9% NaCl, transconjugants were selected on LB plates for the chromosomal and SGI1 markers of the recipient (NalSmSp) and the transferred marker of the test plasmid (Cm).

SGI1-C, a derivative of full-length wt SGI1 identified in a Hungarian *Salmonella* Typhimurium DT104 isolate ST1375 (34,23), was used in matings and as template for generating knockout (KO)/Δ fragments or amplicons for subcloning of SGI1 fragments. The Δ1-Δ8 and Δ*sci* (ΔIGR_ORF2) deletion mutants were generated using the one-step recombination method (54), while the DR1 and DR2 KO mutants were created according to the scarless deletion method (55) using the λ Red recombinase producer plasmid, pKD46, and a Cm^R^ template plasmid (pKD3 or pSG76-CS). The integrated Cm^R^ cassette was removed using the FLP recombinase-producing plasmid pCP20 or the I-*Sce*I-producing plasmid pMSZ934 (KODR1/DR2). The following primer pairs were used to amplify deletion fragments: Δ1, sgiPxisdelfor1 – sgiPxisdelrev; Δ2, 003delfor – 004delrev; Δ3, 003delfor – 005delrev; Δ4, 006delfor – 007delrev; Δ5, 006delfor – 012delrev; Δ6, 013delfor – 018delrev; Δ7; 023delfor – 027delrev; Δ8, 044delfor – minidelrev; Δ*sci*, NCR_ORF2 – delNCRfor – delNCRrev. DR1 and DR2 KOs were generated using the primer pairs KODR1_ABfor – KODR1_Crev and KODR2_ABfor – KODR2_Crev, respectively. To generate double and triple mutants, DR1 was knocked out in the KODR2 strain, and IGR_ORF2 was deleted in the KODR1 + KODR2 double mutant.

KO fragments were electroporated using a BTX Electro Cell Manipulator 600 and 2-mm gap electroporation cuvettes as described previously (56). To maintain and cure the plasmids with a temperature-sensitive pSC101 replication system, incubation at 30°C and 42°C was applied, respectively.

β-galactosidase assays (57) were performed in four biological replicates with some modifications (21). P*_parA_* promoter activity was measured using the β-galactosidase test plasmid pMSZ1260 (Sm^R^Ap^R^) harbouring the 173-bp upstream region of the *parAB053* operon of R55 in front of a promoterless *lacZ* gene. Measurements were conducted in the presence or absence of R55^ΔTn^ (Cm^R^), R16a (Km^R^), the single copy minimal IncC plasmid, pMSZ1248 (Cm^R^), or the pBeloBac11-derived expression vector, pMSZ1239 (Km^R^), with the p15A-based IGR-bearing plasmids, pJKI1149 (Km^R^) or pMSZ1164 (Cm^R^), or the negative control plasmids, pJKI88 (Km^R^) or pJKI405 (Cm^R^). The test plasmid and the p15A-based IGR-bearing or negative control plasmids were co-transformed into *E. coli* strain TG1Nal harbouring R55^ΔTn^, R16a, pMSZ1248, or pMSZ1239. Starter cultures were grown in LB medium supplemented with antibiotics selecting for all plasmids at 37°C ON. These cultures were diluted 100-fold in LB + antibiotics and grown at 37ºC to OD_600_ ∼0.3. In the case of pMSZ1239, expression of ParAB053 proteins was induced with 0.1% l-arabinose during growth to OD_600_ ∼0.3.

### Plasmid stability assays

To monitoring IncC plasmid stability of the in the presence of wt SGI1-C or its mutant derivatives, R55^ΔTn^ (Cm^R^) was conjugated from *E. coli* TG90/R55^ΔTn^ (Tc^R^Cm^R^) into an *E. coli* TG1Nal (Nal^R^) recipient strain carrying Sm^R^Sp^R^ SGI1-C^wt^ or one of the Δ1–Δ8 mutants. Transconjugants harbouring both SGI1 and the IncC plasmid were selected on LB plates supplemented with Nal,Sm,Sp,Cm.

When the stability of R55^ΔTn^ was assayed in the presence of SGI1-derived fragments cloned into different copy-number plasmid vectors, empty plasmids (used as negative control) and their SGI1-fragment-containing derivatives were transformed into *E. coli* strain TG1Nal/R55^ΔTn^ (Nal^R^Cm^R^). Transformants harbouring both R55^ΔTn^ and one of the test plasmids were selected on LB plates supplemented with Cm,Km or Cm,Ap. For *in trans* systems, Cm,Km,Ap selection was applied.

The truncated SGI1 of pJKI670 (and its derivatives in pGMY14, pGMY20, and pGMY28) originated from the SGI1-C variant of the *S*. Typhimurium DT104 isolate, ST28S/1, which emerged spontaneously from a wt SGI1 in the parental strain ST1773 (34). The d1 deletion between DR1 and DR2 repeats occurred during the entrapment of SGI1-C, resulting in the plasmid pJKI669, which carries the SGI1-Cd1 variant (formerly designated SGI28S/1d1). Since the SGI1-Cd1 variant has a different origin from that of our wt SGI1-C, it was sequenced and several SNPs were identified as follows: T9C in DRL, A53G in ORF S017, an A deletion 150 bp upstream of S018, and T499A, T502G, A530C, G557A, C669A and C582A in ORF S044. All other cloned SGI1 fragments were amplified from the wt SGI1-C.

Assays were conducted with four biologically independent replicates, as described in (38), with some modifications. Single transconjugant or transformant colonies were grown ON at 37°C in 2 ml LB broth under selection for R55^ΔTn^ and SGI1, or the test plasmids. Then, 100 μl of a 10^5^× dilution of the starter culture (several thousand cells) was transferred into 5 ml fresh medium and cultured at 37°C to stationary phase, with selection for SGI1 or the test plasmids, but without selection for R55^ΔTn^. Passaging (approx. 20 generations/passage) was repeated daily five times. Cultures of each passage were serially diluted and the cell counts were determined on LB agar plates with selection for SGI1 (Sm,Sp) or the test plasmids (Km or Ap or Km,Ap in the cases of *trans* systems) to determine the total number of cells, or for R55^ΔTn^ (Cm) to determine the frequency of cells that retained the IncC plasmid. Rate of IncC plasmid loss was calculated as the ratio of R55^ΔTn+^ to total cell titres, mean and standard deviation values of the replicates were plotted against the number of passages. In most cases, stability assays were repeated 2–4 times, but references (e.g. pJKI670, pGMY64, pJKI1153) (Supplementary Dataset 1) and negative controls were tested several times (pJKI88: 4 replicates in 31 experiments, pJKI298: 4 replicates in 4 experiments). Data from all assays were used to calculate mean and standard deviation values and for statistical comparisons. When low rates of plasmid loss were observed by titration, R55^ΔTn^ retention in the 5th passage was also confirmed by replica plating. In these cases, 10^6^× dilutions of the four parallel cultures were pooled and spread onto LB agar plates supplemented with antibiotics selective only for SGI1 or the test plasmids. After ON incubation at 37°C, colonies were replica plated onto LB + Cm to determine the proportion of R55^ΔTn+^ colonies.

To monitor the stability of Cm^R^ SGI1-based minimal replicons in the presence of the R55-derived *parAB053* operon, pMSZ1175b and pMSZ1200 were transformed into TG1 cells containing the Km^R^ plasmids, pJKI625 (negative control) or pJKI1128 (P*_ara_*::*parAB053*). Four transformant colonies with every plasmid combination selected on LB + Km + Cm plates were grown ON in LB + Km + Cm broth at 37°C, then 100 μl of 10^5^× dilutions of ON cultures was transferred into 5 ml LB broth supplemented with Km and 0.001% l-arabinose. Passaging was repeated 5 times, as described above.

For stability assays under nonselective conditions, four colonies containing R55^ΔTn^ and wt SGI1-C, or one of the single (KODR1 or KODR2), double (KODR1 + KODR2) or triple (KODR1 + KODR2+Δ*sci*) KO mutants, were grown ON in LB + Nal + Cm + Sm + Sp broth (selecting for both elements). Then, 40 μl cultures from the 10^5^× dilution (∼400 cells) were transferred into 2 ml fresh LB broth supplemented with 0.1% glucose and grown to stationary phase without antibiotic selection, to obtain cell populations of ∼22 generations, respectively. Total cell counts of four replicates were determined on LB + Nal agar plates. Proportions of cells retaining R55^ΔTn^ and/or SGI1 were determined by replica plating onto LB + Nal + Cm, LB + Nal + Sm + Sp, and LB + Nal + Cm + Sm + Sp plates. The percentages of bacteria harbouring R55^ΔTn^, SGI1 or both were calculated as the fractions of Cm^R^Sm^S^Sp^S^/Nal^R^, Cm^S^Sm^R^Sp^R^/Nal^R^ and Cm^R^Sm^R^Sp^R^/Nal^R^ colonies, respectively. Ratios of the subpopulations of cells that lost both SGI1 and R55^ΔTn^ were calculated as Cm^S^Sm^S^Sp^S^/Nal^R^, where Cm^S^Sm^S^Sp^S^= Nal^R^–(Cm^R^Sm^S^Sp^S^+ Cm^S^Sm^R^Sp^R^+ Cm^R^Sm^R^Sp^R^).

### Mobility shift assay

*Purification of ParB protein*. Overnight culture of *E. coli* strain Tuner (DE3) transformed with pMSZ1166 (pET16b-based ParB-producer) was diluted 100-fold in 25 ml LB + Ap broth and grown to an OD_600_ of 0.5 at 37ºC. The culture was induced with 0.3 mM IPTG at 30ºC for 3 h under vigorous shaking, then bacteria were harvested by centrifugation and resuspended in 1 ml lysis buffer (50 mM Tris pH 8.1, 300 mM NaCl, 0.01% Triton X-100) supplemented with 60 μg/ml lysozyme and 30 μl Complete protease inhibitor cocktail (Roche) prepared according to the manufacturer's recommendations. Cells were frozen at –70ºC, then thawed and sonicated on ice at 50% activity for 4 × 10 s. Lysates were centrifuged at 16000 g for 30 min at 4°C and supernatants (cleared lysates) used for purification of ParB with a Dynabeads^®^ His-Tag Isolation & Pulldown Kit (Novex Life Technologies), according to the manufacturer's protocol. Purified protein (∼250 ng/μl) was kept on ice until use.

*Labelling of DNA fragments*. The high copy plasmid pEMBL19 and its derivatives, pMSZ1174, pMSZ1177, pMSZ1178, pMSZ1256 and pMSZ1257, served as templates for PCR amplification using pucfor24_5′FAM and pucrev25_5′FAM primers labelled at the 5′ end with 6-carboxyfluorescein (FAM). Labelled amplicons (138, 126, 139, 139, 167 and 158 bp, respectively) were purified on 6% non-denaturing polyacrylamide gel, according to (21).

*Electrophoretic mobility shift assays (EMSA)*. Binding reactions were performed in a final volume of 10 μl containing 10 mM Tris pH 7.5, 50 mM KCl, 1 mM MgCl_2_, 0.5 mM DTT, 5% glycerol, 1 μg poly [d(I-C)], 3 ng labelled DNA fragment, and different amounts of purified ParB protein (0, 30, 75, 150, 300, 600, 900 ng). Samples were kept on ice for 15 min and loaded onto a 6% non-denaturing polyacrylamide gel for electrophoresis in TBE buffer at 8 V/cm and 4 ºC. FAM-labelled DNA fragments were detected in gels using a ChemiDoc™ MP Imaging System (Bio-Rad). For binding specificity tests, 3 ng of labelled, 75 ng (25-fold) of unlabelled competitor DNA fragment, and 300 ng protein were added to binding reactions.

### Real-time quantitative PCR (RT-qPCR) for relative quantification of the copy number and excision of DR1 KO, DR2 KO and *Δsci* mutant SGI1-C

Copy numbers of free circular SGI1 per cell (*attP*), and the unoccupied integration site (*attB*) in TG1Nal strains carrying wt, or the DR1 KO, DR2 KO, and Δ*sci* (ΔIGR_ORF2) mutant SGI1-C, in the presence of R55^ΔTn^ were determined by RT-qPCR. Amounts of *attB* and *attP* were normalized to those of the single-copy chromosomal gene, *trmE*. The TG1Nal::*attP*_SGI1_ strain, containing single chromosomal copies of *att*B and *attP* (18), was used to calibrate the RT-qPCR assay. The LJ3-RJ5, attsgifor2-attsgirev2 and attsgifor2-attsgirev3 primer pairs were used to amplify specific 251, 257 and 207 bp *attP, attB* and *trmE* fragments, respectively. For RT-qPCRs, total DNA was extracted using the EXtractMe Kit (Blirt DNA, Gdansk), according to the manufacturer's instructions. RT-qPCRs were performed in a final volume of 10 μl using a LightCycler^®^96 detection system (Roche). Each reaction mixture contained 1× qPCRBIO SyGreen Lo-ROX master mix (PCR Biosystems), 400 nM forward and reverse primers, and 10 ng total DNA as template. PCR conditions were: initial denaturation at 95°C for 2 min; 40 cycles of 95°C for 5 s and 60°C for 30 s. Amplification specificity was confirmed by generating melting curves. Relative amounts of amplified *attP* and *attB* sequences were calculated based on the Ct deviation of samples compared with the single-copy-containing control sample, TG1Nal::*attP*_SGI1_, and are expressed relative to the calibrator (*trmE*) sequence. Consequently, the ratio of *attP* and *trmE* was used to measure excised SGI1 copy number, while the ratio of *attB* and *trmE* measured the excision of the island. Results are presented as mean and standard deviation values of data obtained from total DNA of four individual colonies.

### Bioinformatics and statistical analyses

Multalin Interface (58) was used to generate alignment of *parS* sites. The sequence logo was created using the WebLogo server (59). Promoter motifs were predicted using BPROM (60) and BDGP (61), as well as by manual search. All homology searches were performed using the NCBI BLAST server. SGI1-related elements in GenBank were identified via nucleotide BLAST search using the previously defined SGI1 backbone (22) as the query sequence. Putative small RNA secondary structures were predicted using mFold server (62). Protein structures were predicted using Phyre^2^ (63), PSIPRED (64) and AlphaFold (65,66). Pfam (67) was used for protein family or domain searches.

Statistical analyses for comparisons of R55^ΔTn^ stability in the presence of test plasmids containing different SGI1 fragments were carried out by fitting a monoexponential decay model to the plasmid presence ratio data with passage number as an independent variable. The decay model was expressed in its common version of $\mu = {2}^{( - pn/{t}_{1/2})}$where μ is the mean predicted amount of plasmid expressed as a percentage of the initial 100%, *pn* is the passage number, and *t*_1/2_ is the virtual passage number at which the plasmid amount drops to 50%. This model allows direct estimation of the half-life parameter (*t*_1/2_) for each sample, together with the corresponding 95% confidence limits by means of nonlinear regression analysis. Comparisons of the decay kinetics between the samples and references were then conducted by computing the ratios and 95% confidence limits of the corresponding half-life parameters, and significant influences were identified when the confidence interval of the ratio did not contain 1.0. Regression models fitted to the measured data points were plotted against passage number to visualize the decay kinetics of the samples and the references in the same figures (Supplementary Dataset 1). Due to the limited number of experiments, no adjustment for multiple comparisons was applied to avoid the loss of statistical power in detecting practically important differences in decay kinetics. All statistical analyses presented in Supplementary Dataset 1 were carried out using the web-based academic version of SAS Studio 3.81.

## Results

### Deletion mapping of SGI1 functions responsible for destabilization of IncC plasmids

As a first approach to identify SGI1 regions that contribute to expelling IncC plasmids, the stability of R55^ΔTn^*^6187^* (abbreviated as R55^ΔTn^), a Flo^R^/Cm^R^Km^S^Gm^S^Ap^S^ derivative of the IncC Type2 plasmid, R55, was examined in an *E. coli* TG1Nal host harbouring wt or different deletion mutants of SGI1-C (Figure 1A). Since several previous observations published recently (21,24,36,18,37) indicate that wt SGI1, wt SGI1-C, and especially the replication deficient SGI1 derivatives are unstable in the presence of the IncC helper plasmids without selection, to identify and analyse SGI1-encoded factors responsible for IncC plasmid destabilization, antibiotic selection for SGI1 was applied to avoid SGI1 loss during passaging. Although there have been reports of wt SGI1 being completely stable besides IncC plasmid pRMH760 (38,39), based on our own and others' experience, and due to a consistent experimental setup, all strains were grown without antibiotic selection for R55^ΔTn^ but with selection for SGI1-C wt and mutants for 5 passages (approx. 20 generations/passage), and the proportion of cells retaining R55^ΔTn^ was determined at each passage. As observed previously, the plasmid was rapidly lost from a host carrying wt SGI1-C. The proportion of R55^ΔTn+^ cells dropped to approximately 10% at the first passage and to 0.3% at the second. After 100-generations of growth, no R55^ΔTn+^ cells were detected (<0.073%). Similar dynamics of plasmid loss were observed if the S023–S027 (Δ7) or S044 (Δ8) regions were deleted from SGI1-C (Figure 1B, C, Supplementary Figure S1), indicating that these deletions had no effect on SGI1-mediated incompatibility. The next class of deletions affected the excision, replication, or control of these functions (Δ1: P*xis-oriV*, Δ2: *repA-S004*, Δ3: *repA-traN*_S_, Δ4: *sgaDC*, Δ5 *flhC*_SGI1_-*traH_S_*, Figure 1A) and impeded SGI1 in reaching its normal 6–8 excised copies per cell (18,37). These deletions significantly reduced incompatibility with R55^ΔTn^ and caused moderate plasmid loss, as 40–70% of cells retained the plasmid after the 2nd passage and a considerable fraction of the population (10%–40%) were R55^ΔTn+^ even after the 5^th^ passage. The only exception was Δ3, eliminating the *repA-S004-traN*_S_ region, which caused somewhat slower plasmid loss, detectable only by replica plating after the 5th passage (approx. 86% of cells were R55^ΔTn+^, Figure 1B, C). The fact that these deletions reduced, but did not fully eliminate, the incompatibility suggested that the major factor responsible for plasmid loss was still present in these mutants. Only the Δ6 deletion, removing the S013-S018 region including six short ORFs with unknown functions, led to high plasmid stability as no R55^ΔTn^-free cells were observed, even after 100 generations of growth in this case (R55^ΔTn−^ < 0.23%, Figure 1C).

These results directed our attention towards the S013–S018 region, however, the high level of deviation observed among parallel samples in stability experiments (mainly in cases where the deletion interfered with excision or replication of SGI1, Supplementary Figure S1), and the fact that fine mapping of the functions of interest would require many directed mutagenesis changes to the chromosomally integrated SGI1-C, forced us to change our experimental set up to a plasmid-based system.

**Figure 2.**
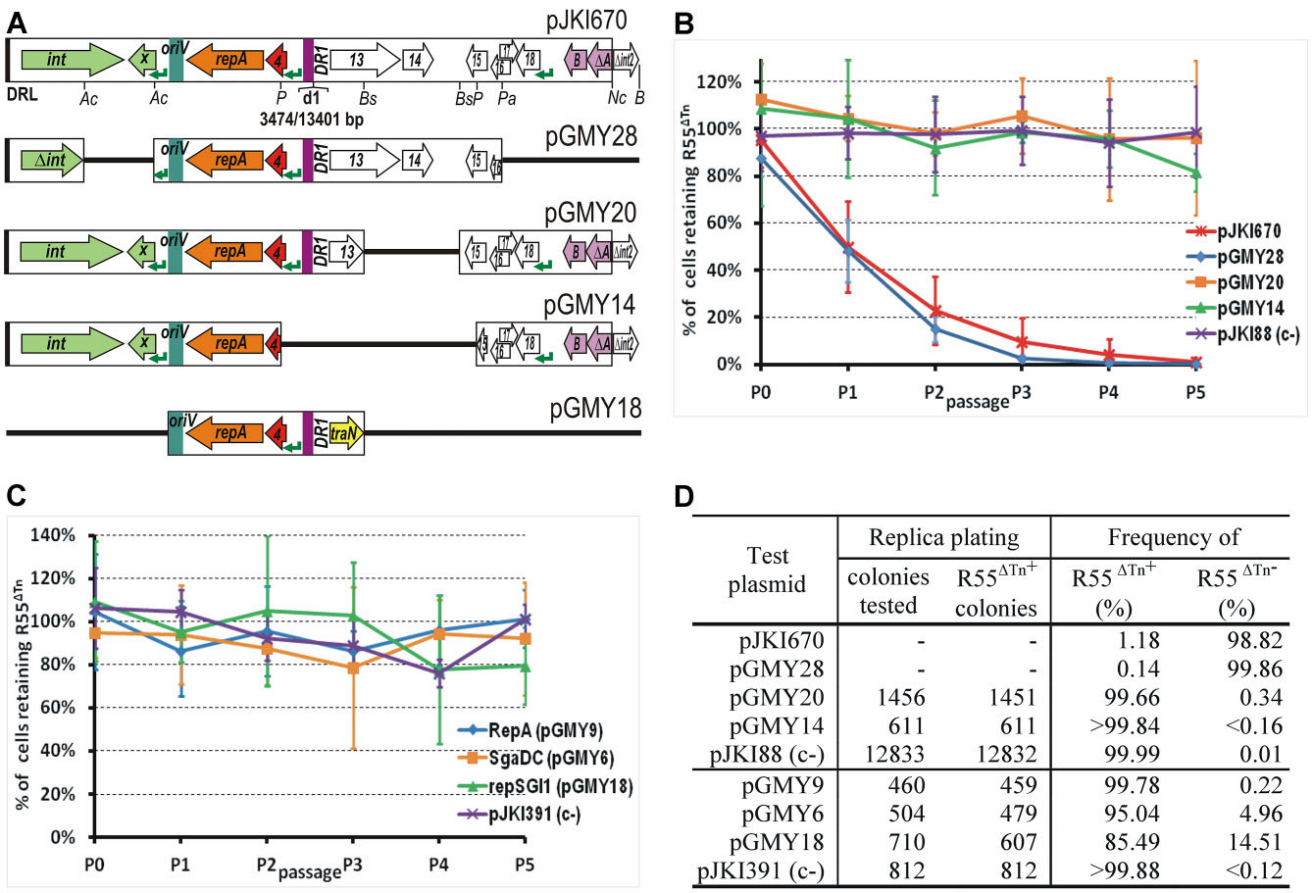
Destabilization of R55^ΔTn^ by the 5′-part of SGI1-C-d1 variant and its further truncated derivatives. (**A**) Map of the p15A-based plasmids containing parts of SGI1-C-d1. The 5′-part of SGI1-C-d1 present in pJKI670 includes SGI1 from DRL to the *Nco*I site of the *mpsB* gene, but lacks the S005-S012 region due to the ‘d1’ deletion occurred between DR1 and DR2 repeats. Deletion derivatives of pJKI670 were generated by cloning steps using the restriction enzymes indicated below the plasmid map (Ac – *Acc*I, P – *Pst*I, Bs – *Bsa*BI, Pa – *Pas*I, Nc – *Nco*I, B – *Bam*HI). ‘d1’ indicates the spontaneous deletion removing the 3474–13401 bp region of SGI1, other symbols are as in Figure 1A. (**B**) Dynamics of R55^ΔTn^-loss from TG1Nal strain harbouring R55^ΔTn^ along with pJKI670, pGMY28, pGMY20, pGMY14 or pJKI88 (empty vector used as negative control). Graph shows the mean percentage of the R55^ΔTn+^ cells in biological replicates grown under selection for the p15A plasmids (Km), but without selection for R55^ΔTn^, through 5 passages as described previously. For individual segregation curves see Supplementary Figure S2A. (**C**) Dynamics of R55^ΔTn^ loss from TG1Nal strains harbouring one of the plasmids expressing *repA*_SGI1_ (pGMY9), *sgaDC* (pGMY6) or containing the entire *rep* region of SGI1 (pGMY18). The empty expression vector, pJKI391, was used as a negative control. The data presented on the graph were obtained in similar stability assays described in part B. For individual segregation curves see Supplementary Figure S2B (**D**). Proportion of R55^ΔTn+^ cells after the 5th passage (100 generations growth) in the cultures assayed in parts B and C. Data were obtained and the threshold limit was calculated as described in Figure 1C.

We have previously captured SGI1 variants in an F plasmid-based trap vector (34) and one of them, SGI1-C-d1 (formerly known as SGI28S/1d1), carried a large deletion (designated d1) that was generated by recombination between two 31-bp imperfect directly repeated sequence motifs, DR1 and DR2, located upstream of the promoters P*_S004_* and P*_traHs_*, respectively (Figure 1A). This spontaneous deletion removed the entire *traN_S_-traH_S_* region, including *sgaDC*, without altering the S013–S018 region. The SGI1-C-d1 variant was cloned into a p15A-based vector (copy number, around 15/cell) and the 3′-part of SGI1 from within *mpsB* was deleted. The resulting plasmid, pJKI670 (Figure 2A), was used to test whether this truncated SGI1 still exerted incompatibility with R55^ΔTn^. SGI1-IncC incompatibility was examined using assays similar to those described previously, except that the SGI1 regions were cloned into a p15A-based plasmid vector and introduced to the R55^ΔTn^-bearing host by transformation. While the p15A-based empty vector, pJKI88 (used as negative control), proved fully compatible with R55^ΔTn^, pJKI670 caused rapid R55^ΔTn^-loss, confirming that the SGI1-derived incompatibility factor was still present in the test plasmid (Figure 2B), however, loss of R55^ΔTn^ in this system appeared to be somewhat slower than that induced by wt SGI1-C (see Figure 1B and Figure 2B). To confirm our previous results, four deletions were generated in pJKI670 (Figure 2A, Supplementary Figure S2A) and the incompatibility of these constructs with R55^ΔTn^ was monitored by the method applied for SGI1-C deletion mutants. As expected, partial or complete removal of the S013–S014 region (pGMY14 and pGMY20) abolished R55^ΔTn^ loss, although the smaller deletion, which left the P*_S004_*–DR1 region intact, did not entirely extinguish the incompatibility, as very low-frequency R55^ΔTn^ loss was detectable in the 5th passage (Figure 2ABD). In contrast, a deletion inactivating the *int* and *xis* genes and removal of the entire *mpsAB* region from within S016 (pGMY28) did not significantly alter R55^ΔTn^ loss dynamics relative to unmodified pJKI670 (Figure 2A, B, D, Supplementary Dataset 1A).

The fact that deletions affecting SGI1 excision, replication, or their control reduced, but did not eliminate, the incompatibility (Figure 1BC) suggested that *repA*, the entire *rep* region or *sgaDC* may contribute to this phenomenon. To examine the incompatibility with R55^ΔTn^ exerted directly by these genes, plasmids expressing *repA* and *sgaDC* from the P_tac_ promoter (pGMY9 and pGMY6, respectively) or containing the entire *rep* region (pGMY18) were assayed as described previously. All three constructs caused low-frequency loss of R55^ΔTn^, which was only detectable at the 5th passage by replica plating (Figure 2C, D, Supplementary Figure S2B) and could not account for the strong incompatibility observed with wt SGI1 or its truncated derivative cloned in pJKI670. These results (Figure 2) support that the major factor(s) mediating incompatibility of SGI1 with R55^ΔTn^ is encoded in the S013–S014 region, although roles of SGI1 replication and *flhDC* expression in this phenomenon cannot be completely excluded.

### Fine mapping of the S013-S014 region for identification of incompatibility factors

**Figure 3.**
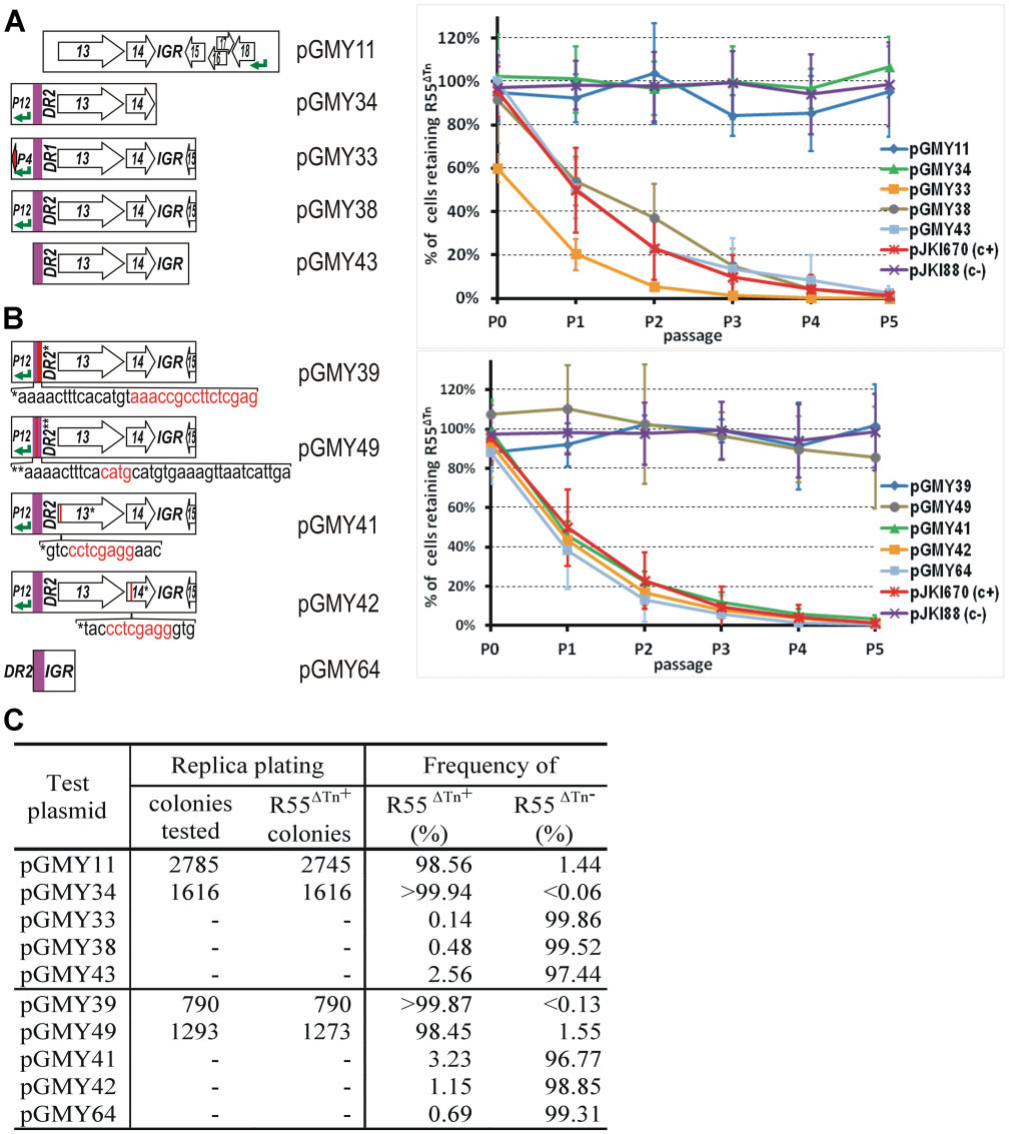
Fine mapping of incompatibility factors in the surroundings of S013-S014 ORFs. Maps of SGI1 fragments cloned in the p15A-based vector pJKI88 are shown in scale. Charts show the dynamics of R55^ΔTn^-loss during 5 passages. For comparison, curves obtained with pJKI670 (positive control) and the empty vector pJKI88 (negative control) are also shown. **(A**) Effects of replacement of P*_S004_*-DR1 with the P_S012_-DR2 and deletion of DR2 or IGR region on R55^ΔTn^-loss. (**B)** Effects of replacement or insertion mutations in DR2, S013 and S014 on R55^ΔTn^-loss. Positions of mutations are indicated by vertical red lines. Replaced or inserted bases are in red. DR2* – the last 16 bp of DR2 is replaced, DR2** – the *Pci*I site of DR2 was filled in leading to 4 bp insertion, 13* and 14* – frameshift mutations generated by insertion of an 8-mer *Xho*I linker into the *Hinc*II or the *BsaA*I site of S013 and S014, respectively. (**C**) Proportion of R55^ΔTn+^ cells in the 5th passages assayed in parts A and B (determined as described in Figure 2D). For individual segregation curves see Supplementary Figure S3.

Our further analysis focused on the region whose deletion caused complete loss of incompatibility with R55^ΔTn^ (pGMY14, Figure 2A). This fragment of SGI1-C-d1 includes the 5′-part and promoter region of S004, the DR1 repeat, the ORF S013 and S014, the intergenic region (IGR) between S014 and S015 and the 3′-part of S015. A series of plasmid constructs were created to identify which part of this segment was responsible for the incompatibility. Shortened or site-directed mutated segments (Figure 3A) were cloned into the p15A-based vector, pJKI88, and the incompatibility of the resulting plasmids with R55^ΔTn^ monitored as previously. The results obtained with plasmids, where the 5′- or 3′-part of the analysed region was deleted or exchanged, indicated that both ends carry elements involved in the incompatibility. Removal of the DR motif (pGMY11) or the entire IGR (pGMY34) almost completely ablated the incompatibility with R55^ΔTn^, however, some plasmid loss was detectable at the fifth passage (see pGMY11). In contrast, restoration of the original constitution present in wt SGI1 by replacing the P*_S004_*–DR1 segment with the P*_S012_*–DR2 located upstream of S013 (pGMY33 versus pGMY38), or even removal of P*_S012_* (pGMY43) did not lead to any sizeable change in incompatibility (only slightly alleviated it) (Figure 3A, C, Supplementary Dataset 1A). Based on these data, the shortest region exerting similarly strong destabilization of R55^ΔTn^ to that observed with pJKI670 (Figure 2B) was the DR2–IGR segment of SGI1 (pGMY43, Figure 3A, Supplementary Dataset 1A).

Since the 31-bp DR motif proved to be an important factor in the incompatibility, the DR2 sequence was first modified by replacing 13 bp at its 3′ end and by filling in the *Pci*I site located in the centre of a 22-bp inverted repeat (IR) embedded in the 5′-part of the DR. Both mutations drastically reduced the incompatibility with R55^ΔTn^ (pGMY39, pGMY49, Figure 3B, C, Supplementary Figure S3). The next question was whether the ORFs S013 and S014 are also involved in this phenomenon. Both ORFs were individually knocked out by inserting an 8-bp *Xho*I-linker into a unique restriction site localized near the 5′ end of the ORFs. Although the insertions caused frameshifts, no remarkable effects were detected, however, both resulted in a minor strengthening of the incompatibility (compare pGMY38, pGMY41 and pGMY42, Figure 3B, C, Supplementary Figure S3, Supplementary Dataset 1B). Thus, the minimal sequence causing a similar (somewhat higher) rate of R55^ΔTn^ loss to that induced by the original SGI1-derivative clone, pJKI670, was achieved by abutting the DR2 repeat and the IGR (pGMY64, Figure 3B, C, Supplementary Dataset 1A, C).

### The incompatibility exerted by DR2 and IGR is dose-dependent

**Figure 4.**
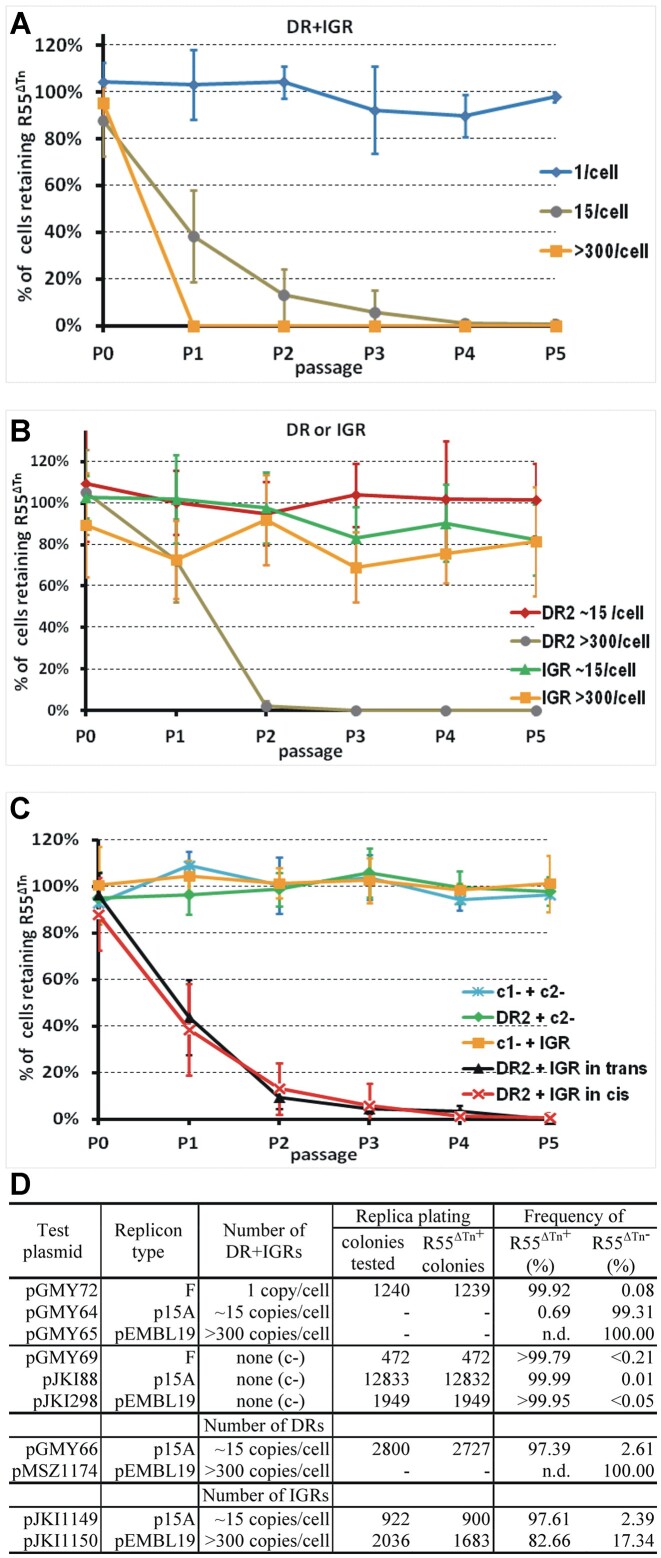
Analysis of DR2- and IGR-promoted incompatibility. (**A**) Copy number dependence of incompatibility exerted by the joined DR2 and IGR sequence. The DR2 + IGR cassette was placed on three different copy-number Km^R^ vectors: pGMY72 (pBeloBac11-derivative, approx. 1/cell), pGMY64 (p15A-derivative, approx. 15/cell, data from Figure 3C are presented here for comparison), pGMY65 (pEMBL19-derivative, >300/cell). In the case of >300 copies of DR2 + IGR (pGMY65), no R55^ΔTn+^ cells were detectable after the 2^nd^ passage. Empty vectors used as negative controls are not plotted as no R55^ΔTn^-loss was observed, thus only the data of replica plating from the 5th passage are shown in panel D for comparison. For individual segregation curves and curves of negative controls (empty vectors: pGMY69 – pBeloBac11-derivative, approx. 1/cell; pJKI88 – p15A-derivative, approx. 15/cell; pJKI298 – pEMBL19-derivative, >300/cell) see Supplementary Figure S4A. (**B**) Analysis of copy number dependence of either DR2- or IGR-promoted incompatibility with R55^ΔTn^. Effects of DR2 and IGR were monitored separately in approx. 15 and >300 copies/cell. The DR2-bearing plasmids were pGMY66 (p15A-derivative, approx. 15/cell) and pMSZ1174 (pEMBL19-derivative, >300/cell), while the IGR-bearing plasmids were pJKI1149 (p15A-derivative) and pJKI1150 (pEMBL19-derivative). In the case of > 300 copies of DR2 (pMSZ1174), no R55^ΔTn+^ cells were detectable after the 3^rd^ passage (see panel D). For individual segregation curves see Supplementary Figure S4B. (**C)** Analysis of incompatibility induced by DR2 and IGR arranged in *trans*. DR2 was provided on pGMY66, while the IGR was placed on the pBR322-derivative pGMY67 (approx. 20/cell). Empty vectors, pJKI88 (c1-) and pBR322 (c2-), were used as negative controls. Bacteria carrying R55^ΔTn^ and one of the four combinations of the p15A and pBR322-derivatives were passaged under Ap + Km selection. R55^ΔTn^ loss in the presence of DR2 and IGR in *trans* showed similar dynamics as observed with DR2 + IGR in *cis* (pGMY64, approx. 15/cell, the curve from Figure 3C is presented here for comparison). For individual segregation curves see Supplementary Figure S4C. (**D**) Proportion of R55^ΔTn+^ cells in the 5th passages assayed in parts A and B (determined as described in Figure 2D). Data of pGMY64 from Figure 3D is also presented here for comparison). n.d. – not detectable in the 5th passage.

The strength of incompatibility is often dose-dependent (40), therefore, the copy-number dependence of the incompatibility was examined by introducing the DR2 + IGR into the R55^ΔTn^-bearing host on different copy-number plasmids: a one-copy pBeloBac11-based (pGMY72), an approx. 15-copy p15A-based (pGMY64), and a >300-copy pEMBL19-based (pGMY65) vectors. The kinetics of R55^ΔTn^ loss indicated robust copy-number dependence of incompatibility (Figure 4A). While plasmid loss was almost undetectable if DR2 + IGR was present at 1 copy/cell (about 0.1% of cells were R55^ΔTn−^ in the 5^th^ passage), >300 copies per cell caused drastic plasmid loss (in the 1st passage < 0.02% of cells were R55^ΔTn+^, and were no longer detectable). In the presence of empty vectors used as negative controls, R55^ΔTn^ loss was undetectable (Figure 4D, Supplementary Figure S4A).

Next, we separated the two elements contributing to the incompatibility to identify which caused the copy number dependence. The DR2 and IGR sequences were cloned separately into a p15A- and a pEMBL19-based vector and their incompatibility with R55^ΔTn^ was assayed. The results showed that both elements caused very weak incompatibility alone at low copy number (approx. 15/cell) as R55^ΔTn−^ cells were only detectable by replica-plating (about 2.5% of cells in the 5th passage), but DR2 and IGR had different effects on R55^ΔTn^ loss when present at high copy number. While the IGR did not show a strikingly stronger effect at high copy number than at 15 copies (17.4% versus 2.6% R55^ΔTn−^ cells in the 5th passage), DR2 promoted robust plasmid loss at high copy number (R55^ΔTn+^ cells were undetectable from the 3rd passage, Figure 4BD, Supplementary Figure S4B). Finally, to test whether they could cooperate in *trans*, DR2 and IGR were supplied on different plasmids with similar copy-number (DR2: p15A-based plasmid pGMY66, IGR: pBR322-based plasmid, pGMY67, ∼20 copies/cell). The results (Figure 4C, D, Supplementary Figure S4C) showed that together, DR2 and IGR promoted similarly strong incompatibility, whether they were present in *cis* or in *trans* (compare pGMY64 and pGMY66 + pGMY67, Figure 4C, Supplementary Dataset 1A, C). These data also indicate that the two elements together exert a much stronger incompatibility than either of them alone (Figure 4).

### SGI1 DR motifs contain functional *parS* sites of IncC plasmids

**Figure 5.**
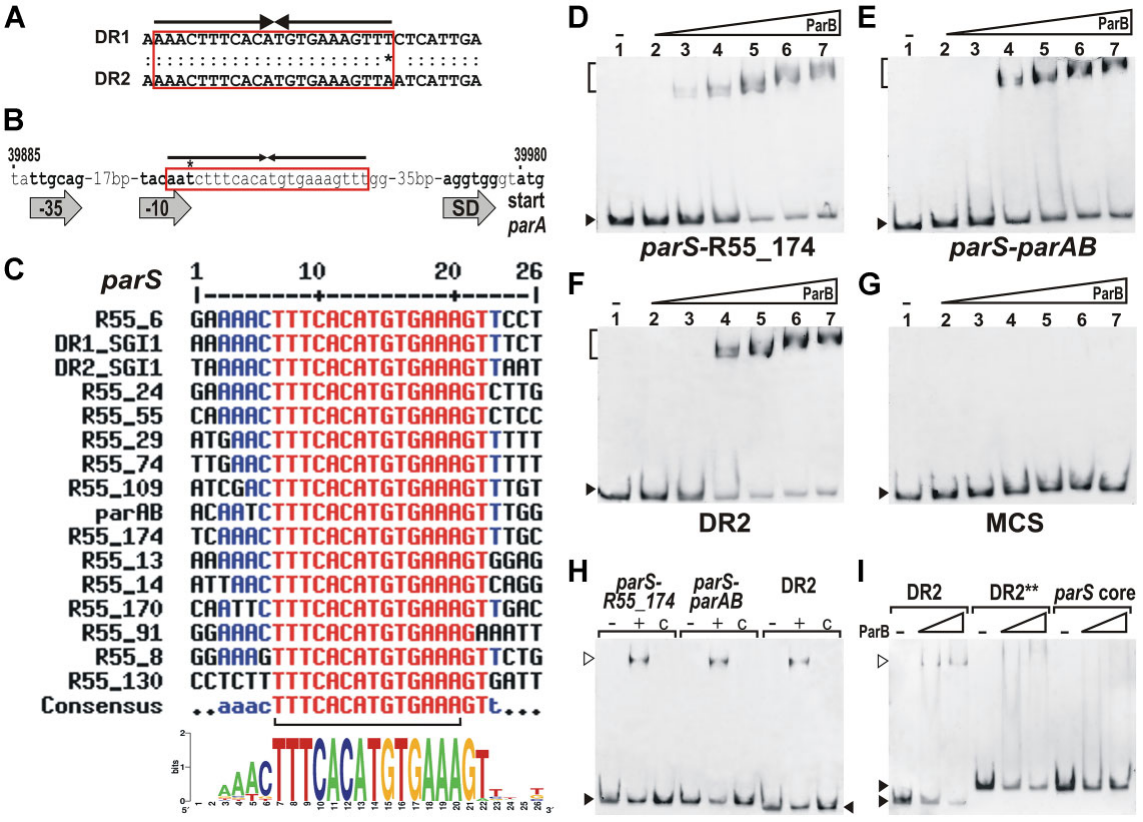
Analysis of putative *parS* sites identified in R55 backbone and the 31-bp DRs of SGI1. (**A**) Comparison of DR1 and DR2 sequences. Symmetry of the 22-bp inverted repeat motifs corresponding to the putative *parS* sites (red box) is shown by arrows. Asterisk indicates the mismatching base in the IR. (**B**) Upstream region of R55 *parA* gene. Predicted promoter boxes (−35, −10), ribosome binding site (SD) and the putative *parS* site (red box) are indicated (symbols are as in panel A). Coordinates shown above the sequence are according to the published R55 sequence (Acc. no.: JQ010984.1). (**C**) Alignment and sequence logo for 16 putative *parS* sites identified in R55 and SGI1. R55-derived sequences are named according to the nearest ORF. In the consensus, red and blue indicate >90% and >60% conserved bases, respectively. The fully conserved symmetrical core sequence is shown by brackets. (D-G) EMSAs for detection of DNA-ParB complex. FAM-labelled probes were: (**D**) the putative *parS* site located in the intergenic region near ORF R55_174, (**E**) the putative *parS* preceding the *parAB* operon, (**F**) the entire DR2 of SGI1 and (**G**) the empty multicloning site (negative control) flanking the *parS* sequences in the other three probes. The *parS* R55_174 sequence represents the IR motif present in DR1 of SGI1 and also the consensus of the 16 IR motifs. Amount of ParB protein was increased from 0–900 ng (lanes 1–7: 0, 30, 75, 150, 300, 600 and 900 ng). Brackets indicate shifted complexes, and filled arrowheads point to the unbound probe. (**H**) Binding specificity tests for three *parS*-containing probes. Binding specificity was confirmed in the presence of 25-fold unlabelled probes. Samples in all lanes contained 3 ng of labelled probes. Lanes ‘-’, no ParB protein; lanes ‘+’ and ‘c’, 300 ng parB protein; lanes ‘c’, 25-fold unlabelled DNA fragments were added to the respective binding reaction run in lanes ‘+’. (**I**) EMSA for detection of ParB binding to wt and insertion mutant DR2, and to the conserved symmetrical 14-bp core sequence of the putative *parS* sites. Probe ‘DR2**’ contained 4-bp insertion at the centre of the 22-bp IR of DR2, and probe ‘*parS* core’ carried the 14-bp symmetrical sequence shown by bracket in panel C. Amount of ParB protein was 0, 150 or 450 ng. In panels H and I, open and filled arrowheads point to shifted complexes and unbound probes, respectively.

A thorough examination of the 31-bp DR motifs identified a 22-bp IR in the 5′-part of the DRs (Figure 5A). IR motifs often serve as binding sites, thus we supposed that the 22-bp IRs in DR1 and DR2 bind a protein involved in IncC plasmid stability. The 22-bp IRs resemble those present at one or more copies in centromere sites of many plasmids and bacterial chromosomes (45). In addition, a very similar IR motif was found in the promoter region of the *parAB* operon of R55 and virtually all other IncC plasmids. Using BProm promoter prediction software, a putative promoter motif was identified 64 bp upstream from the *parA* start codon. The largest part of the 22-bp IR motif resembling those found in SGI1 DRs lies downstream of the predicted promoter, but partially overlaps its –10 box (Figure 5B). The length and position of the IR in P*_parA_* suggested that it may serve as a *parS* site for the plasmid by which the *par* operon is controlled, as a similar genetic constitution can be seen in both the *parABS* and *parMRC* systems of several other plasmids (45). A global homology search of the R55 sequence identified 13 additional IR-like motifs scattered across the plasmid backbone, supporting that these sequences may represent *parS* sites of IncC plasmids. Alignment of 14 plasmid-derived and two SGI1-derived IRs, along with their several flanking bases, indicated that the repeats are assembled from a fully conserved symmetrical 14-bp core sequence, which is flanked by less conserved AAAC and GTTT motifs (Figure 5C). The sequence logo generated for the 16 IRs suggested that the conserved G (16/16) and T (15/16) in the GTTT sequence of the right flanking arm also have an important role, presumably in protein binding.

To examine whether these IRs serve as binding sites for ParB protein, the putative CBP of the IncC ParABS system, ParB was purified (Supplementary Figure S5) and used in EMSAs with three FAM-labelled probes: the entire 31-bp DR2 sequence and the 22-bp putative *parS* sites identified upstream of *parA* and ORF R55_174 (*eexC*), respectively. The latter was chosen because its perfect symmetrical sequence is identical to both the IR motif in the other DR of SGI1 (DR1) and the consensus of the 16 IR motifs. EMSAs showed that ParB protein bound to all three putative *parS* sites, while it did not bind to the empty vector sequence flanking the *parS* sites in the labelled probes (Figure 5D–G). Binding specificity was confirmed by adding 25-fold unlabelled DNA fragments to the binding reactions (Figure 5H). These results support that all 14 plasmid-derived IR motifs can bind ParB and act as actual *parS* sites, and demonstrate that both SGI1-derived IRs found in DR1 and DR2 are also functional ParB binding sites.

Next, binding of ParB to the insertion mutant *parS* sequence in DR2 (DR2**, Figure 3B) and to the 14-bp core sequence of the *parS* sites was also examined by EMSA. The 4-bp insertion at the centre of the *parS* site did not abrogate its symmetry, but abolished ParB binding (Figure 5I), suggesting that the previously observed loss of incompatibility with R55^ΔTn^ by the same mutation (pGMY49, Figure 3B, C) was related to its inability to be bound by ParB. Surprisingly, ParB could not bind the fully conserved symmetrical 14-bp core sequence, affirming that the mostly symmetrical flanking bases, in particular the highly conserved GT bases in the right side flanking sequence, may have an important role in the recognition of *parS* sites. In summary, we conclude that the IncC-related *parS* sites present in DRs of SGI1 are important factors responsible for its incompatibility with IncC plasmids.

### Identification of the incompatibility factor localized in the IGR between S014 and S015

**Figure 6.**
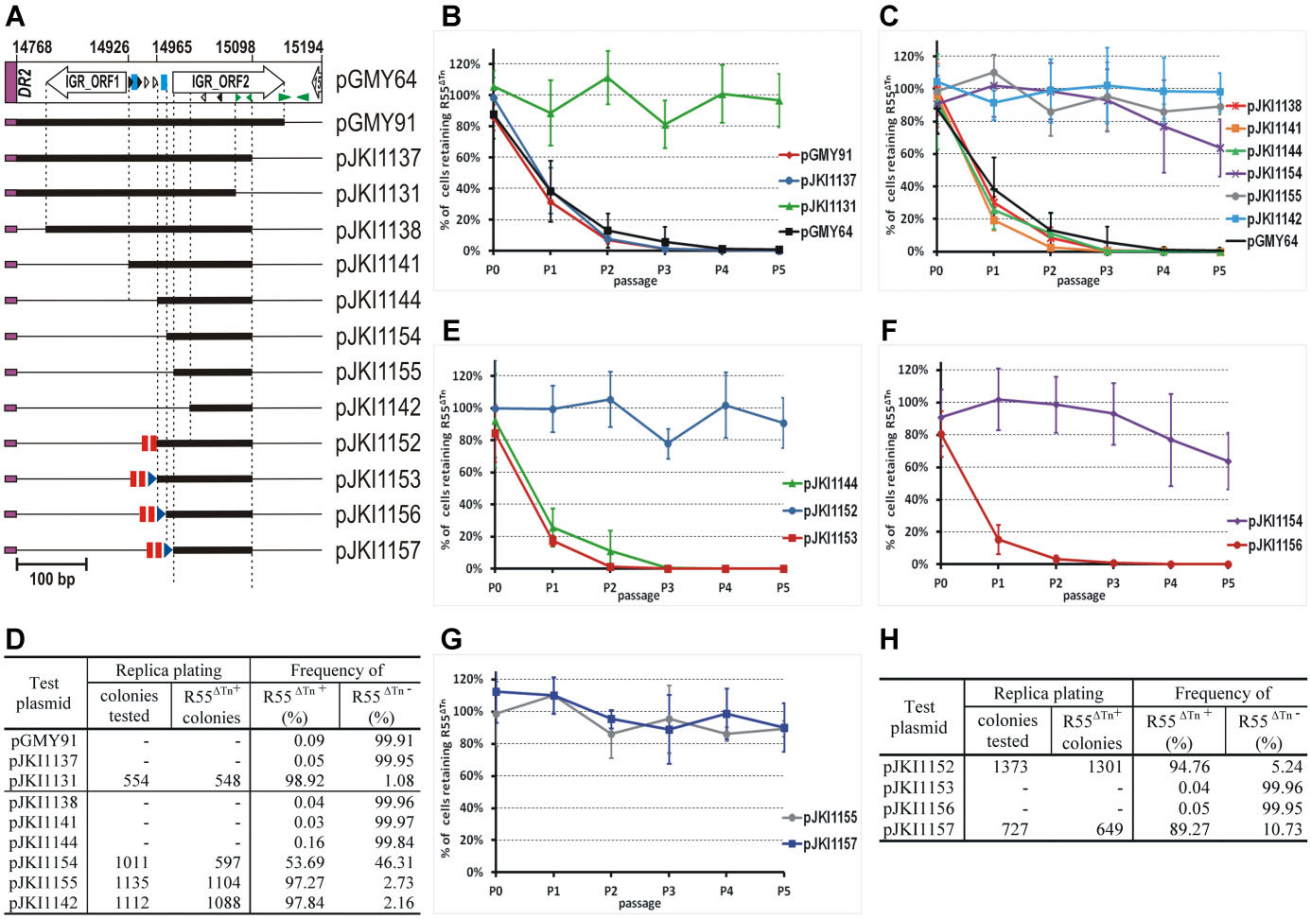
Localization of the incompatibility factor in IGR between ORFs S014 and S015. (**A**) Schematic map of the IGR sequence (drawn to scale) present in the test plasmid pGMY64. Coordinates are according to the published SGI1 sequence AF261825, with the modification that the numbering starts from the 5′ end of DRL. Arrowheads indicate the predicted –35 (filled) and –10 (open) promoter boxes. Blue rectangles show the predicted Shine-Dalgarno (SD) boxes. Short and long green arrowheads indicate the 8/8-bp and the 18/19-bp IR motifs, respectively. Thin lines show the missing regions compared to pGMY64. Double red boxes represent *rrnB*T1 and T2 terminators, and the dark blue arrowhead indicates the P*_cat_* promoter (these are not to scale). (**B** and **C**) Dynamics of R55^ΔTn^ loss from TG1Nal strains harbouring test plasmids with 3′ - and 5′-truncated IGRs. The curve obtained with pGMY64 is also shown for comparison. For individual segregation curves, see Supplementary Figure S6. (**D**) Proportion of R55^ΔTn+^ cells in the 5^th^ passages assayed in parts B and C (determined as described in Figure 2D). (**E–G**) Dynamics of R55^ΔTn^ loss from TG1Nal strains harbouring test plasmids supplemented with *rrnB*T1T2 terminator or the *rrnB*T1T2–P*_cat_* cassette. Data of unmodified test plasmids pJKI1144, pJKI1154 and pJKI1155 presented on panel C are also shown for comparison. For individual segregation curves see Supplementary Figure S7. (**H**) Proportion of R55^ΔTn+^ cells in the 5^th^ passages assayed in parts E–G (determined as described in Figure 2D).

Our data indicated that the IGR between S014 and S015 encodes an unknown factor, which drastically enhances the incompatibility with R55^ΔTn^ exerted by a single DR (Figure 4). The 415-bp IGR region contains two short divergent ORFs, along with putative SD-boxes and promoter-like motifs, which presumably express functional proteins (Figure 6A). Furthermore, there are two imperfect IR motifs near the 3′ end of IGR, which may act as protein binding sites or form stem-loop structures with a regulatory role in a hypothetical RNA. Therefore, we first conducted deletion mapping focused on the role of these elements in incompatibility. Since the IGR alone had a weak effect on R55^ΔTn^ stability (Figure 4), the deletion mapping was performed on plasmid pGMY64, which contained the DR2 joined to the entire IGR and exhibited strong incompatibility with R55^ΔTn^ (Figure 3B). This setup ensured easier detection of any changes in the strength of incompatibility in stability assays. Thus, the test plasmids were pGMY64 analogues containing one of the differently truncated or modified IGR fragments joined to the DR2 sequence.

Three deletions were generated in the 3′ end of the IGR. The shortest removed the downstream noncoding region of IGR_ORF2, along with the majority of the distal 18/19-bp IR motif (pGMY91). The next also removed the last 44 bp of IGR_ORF2, but left the proximal 8/8-bp IR motif (pJKI1137) unaffected, while the longest deletion (pJKI1131) eliminated both IRs, along with the last 68 bp of the ORF (Figure 6A). Stability assays indicated that the two smaller deletions had no significant effect, as pGMY91 and pJKI1137 showed strong incompatibility, comparable to that of pGMY64 (Supplementary Dataset 1C). However, the longest deletion reduced the strength of incompatibility to a similar level to that observed with DR2 alone (Figure 6A, B, D, Supplementary Figure S6, see also DR2-bearing pGMY66 in Figure 4B, D). This result suggests that the integrity of the long IR and the 3′ end of IGR_ORF2 is not important for R55^ΔTn^ destabilization, while the region containing the 8/8-bp IR appears to be necessary.

Next, the 5′-region of the IGR was gradually shortened in a series of plasmid constructs, where the 3′-part was terminated after the 8/8-bp IR, as in pJKI1137. Removal of the non-coding sequence downstream of IGR_ORF1 (pJKI1138) and complete deletion of IGR_ORF1 (pJKI1141) did not considerably change (slightly increased) the strong incompatibility observed with pJKI1137 or pGMY64. Surprisingly, the next deletion, additionally removing the two predicted promoters preceding IGR_ORF2 (pJKI1144) also had no effect (Figure 6A, C, D, Supplementary Figure S6, Supplementary Dataset 1C). In contrast, removal of the entire upstream region of IGR_ORF2, including the putative SD-box (pJKI1154), caused a drastic reduction in incompatibility, however, this construct was still a more efficient destabilizer of R55^ΔTn^, than the single DR2-bearing plasmid, pGMY66. Finally, deletion of the entire upstream region of IGR_ORF2, along the replacement of the original GTG (Val) START codon with a GTC (Val) and the second GCA (Ala) codon with GAC (Asp) (pJKI1155), or additional removal of the first seven codons of IGR_ORF2 (pJKI1142) reduced the incompatibility to the basal level caused by DR2 alone.

Since DR2 and the promoterless IGR_ORF2 together exerted strong incompatibility with R55^ΔTn^, we supposed that an outer promoter controls the expression of the putative ‘incompatibility gene’ in pJKI1144 (e.g. a promoter-like element found in DR2). To confirm this hypothesis, the *rrnB*T1T2 terminator was inserted into pJKI1144 between DR2 and IGR_ORF2 (pJKI1152). This led to loss of incompatibility, which was entirely restored by insertion of the strong P*_cat_* promoter (pJKI1153) downstream of the terminator (Figure 6DEH, Supplementary Figure S7, Supplementary Dataset 1C). Inserting the same *rrnB*T1T2–P*_cat_* cassette upstream of the intact but SD-box-deleted IGR_ORF2 (pJKI1156) also restored the strong incompatibility (pJKI1154 vs. pJKI1156, Figure 6DFH, Supplementary Figures S6 and S7, Supplementary Dataset 1D), while this did not considerably increase the weak incompatibility of the START codon-deleted construct (pJKI1155 vs. pJKI1157, Figure 6D, G, H, Supplementary Figures S6 and S7).

We conclude that an unknown incompatibility factor is expressed from the predicted promoter region located upstream of IGR_ORF2 and can be inactivated by deletion of the START codon or the last 31 codons of IGR_ORF2, including the 8/8-bp IR motif. The importance of intactness of the N-terminal region, and particularly the START codon of the ORF suggest that the sought incompatibility factor is the 51 amino acids (aa) small protein encoded by IGR_ORF2. However, as removal of the last 14 codons (almost one third of the putative protein) had no negative effect on the strength of incompatibility, and given the apparent importance of the 8/8 bp IR in the 3′-part of IGR_ORF2, we considered the possibility that a small RNA is synthesized from this region, and can act as an incompatibility factor.

### A small RNA or a small protein?

**Figure 7.**
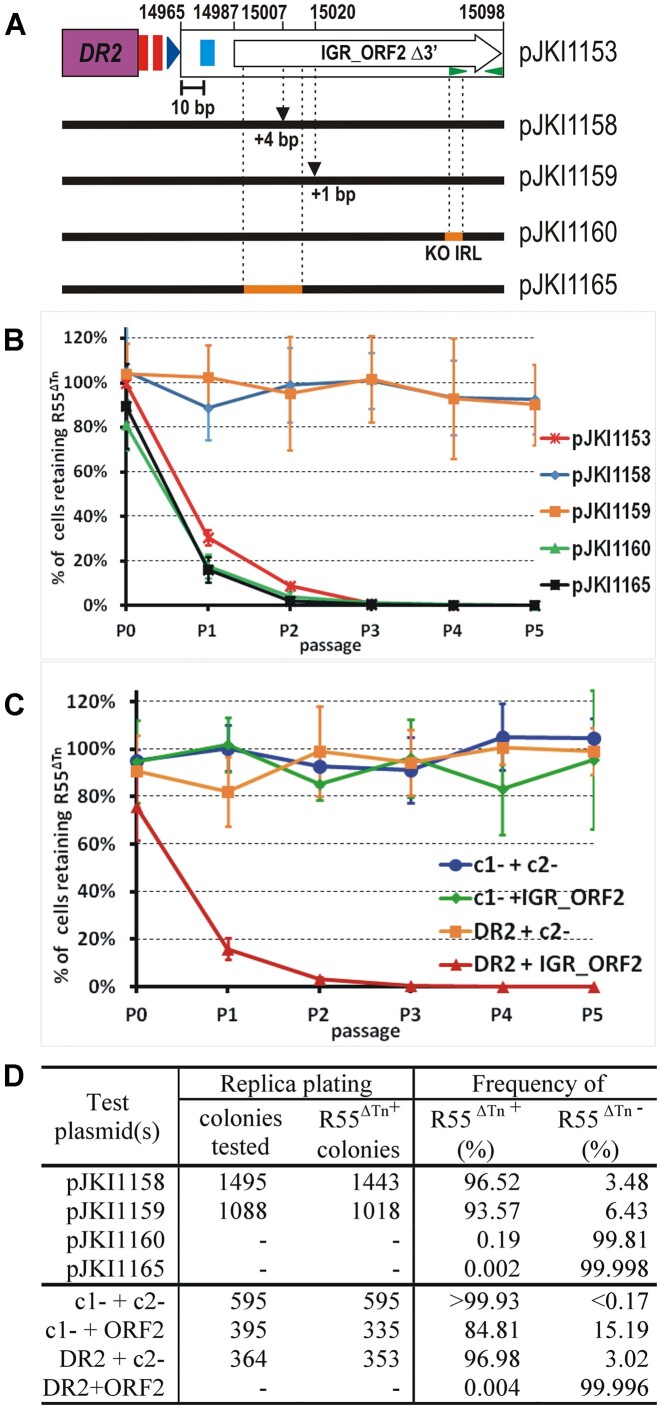
Identification of the incompatibility factor encoded in IGR. (**A**) Schematic map of the functional part of IGR_ORF2 in the test plasmid pJKI1153. The green arrowheads represent the 8/8 bp IR motif. Coordinates and other symbols are as in Figure 6A. The horizontal thick lines represent the relevant part of the mutated test plasmids. The 4 or 1-bp insertions are indicated by arrowheads, and the samesense codon replacements are highlighted by orange. Note that the replacement in pJKI1160 eliminated the left arm of the 8/8-bp IR. (**B**) Dynamics of R55^ΔTn^ loss from TG1Nal strains harbouring the plasmids with modified IGR_ORF2Δ3' (panel A). The curve obtained with the unmodified construct pJKI1153 (Figure 6E) is also shown for comparison. For individual segregation curves see, Supplementary Figure S8A. (**C**) Dynamics of R55^ΔTn^ loss from TG1Nal strains containing the components of the *trans* system in different combinations. DR2 – the DR2-bearing p15A plasmid pGMY66, IGR_ORF2 – the pKK223-3-based expression vector pJKI1161 producing Sci protein (during passages, protein expression occurred by leaking of the P_tac_ promoter without IPTG induction). The empty vectors, pJKI88 (p15A, c1–), and the pKK223-3 derived expression vector pJKI1130 (pBR322, c2–), were used as negative controls. For individual segregation curves see Supplementary Figure S8B. (**D**) Proportion of R55^ΔTn+^ cells in the 5th passages assayed in parts B and C (determined as described in Figure 2D).

To answer this question, we generated several mutations in pJKI1153, which contained DR2 and a 3′ truncated IGR_ORF2 with its 22-bp upstream region under the control of the P*_cat_* promoter (Figure 7A) and exerted strong incompatibility with R55^ΔTn^. First, the unique *Bgl*II site located near the 5′ end of IGR_ORF2 was filled in (pJKI1158), which generated a 4-bp insertion and a frameshift in the ORF. This mutation abolished the incompatibility (Figure 7B, D, Supplementary Figure S8A), reducing it to the level exerted by a single DR-containing pGMY66 (Figure 4B, D), however, the insertion also significantly changed the predicted stem-loop structure in the 5′-part of the presumed small RNA (Supplementary Figure S9A, B), which could also be responsible for the loss of function. The next frameshift mutation was generated by introducing a single base insertion after the 34^th^ bp position of IGR_ORF2 (pJKI1159) in a region not involved in forming specific secondary structures in the RNA, according to secondary structure prediction (Supplementary Figure S9A). Similar to the previous frameshift mutation, this also abolished the incompatibility (Figure 7BD), despite causing no significant change in the predicted RNA structure (Supplementary Figure S9C). Next, two mutations were designed to modify the putative RNA structure, but not the protein sequence. First, two neighbouring Leu codons (aa positions, 30–31) overlapping the left arm of the 8/8-bp IR were reversed (pJKI1160), which destroyed the IR motif and caused a radical change in the predicted RNA secondary structure (Supplementary Figure S9AD) without changing the protein sequence. Then, codons 2–10, overlapping the sites of the two frameshift mutations, were replaced with same-sense codons (pJKI1165), which modified the predicted 5′ hair-pin structure in the RNA (Supplementary Figure S9A, E). Despite these alterations in the putative RNA structure, both constructs exerted similarly strong (or even somewhat stronger) incompatibility with R55^ΔTn^ than the unmutated plasmid, pJKI1153 (Figure 7B, D). These data strongly suggest that the sought incompatibility factor is more likely the small protein encoded by the IGR_ORF2, rather than a small RNA.

For additional proof, a *trans* system was constructed, where the DR2 sequence was supplied by the p15A-based plasmid pGMY66, while the protein was expressed from a pKK223-based vector, where the complete IGR_ORF2 was placed under the control of P_tac_ (pJKI1166). In stability assays, the empty parental vectors of both constructs were used as negative controls and loss of R55^ΔTn^ was examined as previously. Together, the DR2-bearing and IGR_ORF2-expressing plasmids exerted strong incompatibility (Figure 7CD, Supplementary Figure S8B), similar to or even stronger than that obtained using constructs with these elements in *cis* (e.g. pGMY64 or pGMY91, Figure 6B, Supplementary Dataset 1D). As expected, the negative control combinations caused no (c1-/c2-) or low (c1-/IGR_ORF2 and DR2/c2-) rates of R55^ΔTn^ loss. These results are also consistent with those obtained with the system testing DR2 and the whole IGR in *trans* (Figure 4C). Considering all these findings, we conclude that the small protein (named Sci from SGI1-IncC incompatibility) expressed from IGR_ORF2 (the *sci* gene) acts as another SGI1-borne incompatibility factor, in addition to the *parS* sites localized in DR1 and DR2.

Based on protein structure predictions, Sci is mainly helical protein with a 13 aa disordered region at its C-terminus (Supplementary Figure S10), which appears to be unnecessary for the activity (Figure 6A–C). Sci has no homologs in the protein databases among proteins with known functions and does not contain any identified domains or belong to established protein families included in the Pfam database. However, a Blastn search in GenBank using IGR_ORF2 sequence retrieved 225 hits, including the vast majority of identified SGI1 variants and more distant relatives, such as PGI1 variants, PGI2, AGI1, and an unnamed element found on *Shewanella* sp. W3-18-1 chromosome. The wide distribution and high conservation (79–100%) of this small gene in the SGI1-family indicates that the encoded protein has important roles in the stability or propagation of SGI1-like elements.

### A minimal SGI1 replicon bearing a *parS* site is stabilized by the ParABS system of R55

**Figure 8.**
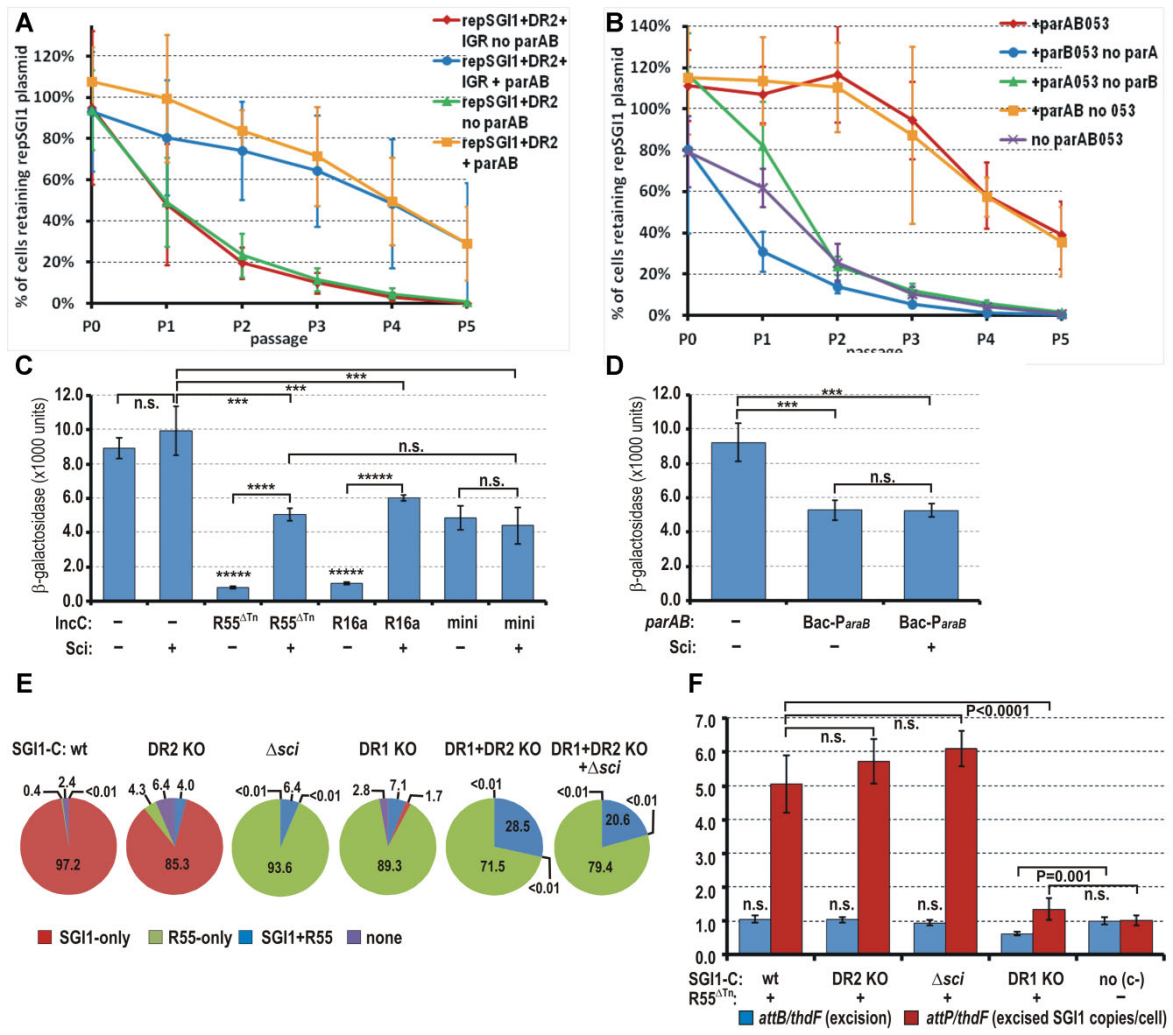
Functional analysis of DRs and the Sci protein produced by SGI1. (**A**) Stabilization of the SGI1-derived minimal replicon containing DR2 or DR2 + IGR in the presence of the *parAB053* operon of R55. Graph shows the dynamics of the loss of the SGI1-derived replicons supplemented with DR2 (pMSZ1200) or DR2 + IGR (pMSZ1175b) from TG1Nal strain with or without the *parAB053* operon (pJKI1128 or pJKI625, respectively) under the control of P*_araB_* promoter. Expression of the *par* operon was induced with 0.001% L-arabinose. The empty expression vector pJKI625 was used as a negative control. For individual segregation curves, see Supplementary Figure S11A. (**B**) Stability of the SGI1-derived replicon carrying DR2 in the presence of the KO mutants of *parAB053* operon expressing no ParA or ParB or 053 proteins. Graph shows the dynamics of the loss of SGI1-derived minimal replicon, containing an IncC *parS* site present in DR2 (pMSZ1200, from TG1Nal strain, where only two of the three proteins of the *parAB053* operon were expressed. ParB + 053 without ParA were expressed from pMSZ1263, ParA + 053 without ParB were expressed from pMSZ1272, and ParA + ParB without 053 were expressed from pGMY86. The three KO mutant expression plasmids were analogous to pJKI1128 that expressed all the three proteins (used as positive control), while pJKI625 (no *parAB053*) was used as a negative control. Expression of the *par* operon was induced with 0.001% l-arabinose. For individual segregation curves, see Supplementary Figure S11B. (**C**) β-galactosidase assay for measuring the effect of the Sci protein on the promoter activity of P*_parA_* in the presence or absence of an IncC plasmid or a minimal IncC replicon. The test plasmid pMSZ1260 carried the P*_parA_*-*lac*Z fusion and Sci was provided in *trans* from the p15A-based vectors pJKI1149 (Km^R^) or pMSZ1164 (Cm^R^) (both containing the IGR), which were applied depending on the selection marker of the IncC plasmids R55^ΔTn^ (Cm^R^) or R16a (Km^R^) or the minimal IncC replicon (Cm^R^). The minimal IncC replicon (pMSZ1248) contained the *rep* region with *oriV* and the *parAB053* operon with its own promoter region. Data were obtained from four independent colonies. (**D**) β-galactosidase assay for measuring the effect of the Sci protein on the promoter activity of P*_parA_* in the presence of ParAB053 proteins expressed from the P*_araB_*::*parAB053* cassette placed on the single copy pBeloBac11 vector (Bac-P*_araB_*= pMSZ1239, Km^R^). To measure P*_parA_* activity, the P*_parA_*-*lac*Z –bearing plasmid, pMSZ1260, was used and the Sci was provided in *trans* from the p15A-based vector pMSZ1264 (Cm^R^) carrying the entire IGR. Expression of Par proteins was induced with 1% l-arabinose. Data were obtained from five independent colonies. In the negative control (no *parAB053*, no IGR), empty vectors pMSZ1242 and pJKI405 were applied along with the test plasmid pMSZ1260. Paired t-test was used to calculate the statistical significance. *****P< 10^−9^; ****P< 10^−6^; ***P< 10^−3^; n.s., not significant (*P*> 0.05). (**E**) Segregation of wt, DR1 KO, DR2 KO and Δ*sci* mutant SGI1-C and R55^ΔTn^ plasmid under nonselective conditions. Diagrams show the distribution of R55^ΔTn^ and SGI1-C after approx. 22 generations growth without selection. Data are means obtained from four independent replicates. For the raw data set, see Supplementary Table S4. (**F**) Excision and copy number of extrachromosomal wt SGI1-C and KO mutants in cells harbouring also R55^ΔTn^. Data of RT-qPCR were normalized to the that of control strain, TG1Nal::*attP*_SGI1_ (marked with ‘c-’ on the chart), containing a single copy of *attB* and *attP*. Paired t-test was used to calculate the statistical significance, n.s., not significant (*P*> 0.05).

Partitioning systems for plasmids or ICEs have crucial roles in their stability. Although this work initially focused on the incompatibility phenomenon observed between SGI1 and IncC plasmids, the fact that SGI1 harbours two copies of the centromere-like *parS* sequence, a *cis*-element of the ParABS system of IncC plasmids, raised the possibility that their role is not limited to plasmid expulsion, but that they may also contribute to SGI1 stabilization. To test this hypothesis, we first inserted DR2 and the DR2-IGR cassette into an SGI1-derived Cm^R^ minimal replicon assembled from *oriV*_SGI1_ and *repA*_SGI1_ placed under the control of promoter P_tac_ (18). The stability of the resulting plasmids was examined in the absence or presence of the *parAB053* operon of R55 expressed in *trans* from the P*_araB_* promoter. Stability assays carried out without selection for the SGI1-based replicons showed that these plasmids were rather unstable in the absence, but became much more stable in the presence, of the *par* operon (Figure 8A, Supplementary Figure S11A). The segregation dynamics also indicated that this effect is mainly due to the presence of DR2 harbouring a *parS* site, as the presence or absence of IGR had no significant effect on the stability of the minimal SGI1 replicon. This result suggests that the Sci protein synthesized from the IGR acts independently from the plasmid stabilization exerted by the ParAB053 proteins.

The stabilizing effect of ParAB053 proteins was further analysed using three KO mutant *parAB053*-expression plasmids, in which each one of the three ORFs (*parA*, *parB* or *ORF053*) was inactivated by a frameshift mutation. In a stability assay, retention of the DR2-bearing minimal SGI1 replicon was examined in the presence of each of these mutant *parAB053*-expressing plasmids. Loss of the minimal SGI1 replicon was similarly low when all the three proteins, or both ParA and ParB, were expressed without the 053 protein (Figure 8B, Supplementary Figure S11B), whereas the SGI1 replicon was drastically destabilized in the absence of either ParA or ParB. Lack of ParA caused even more rapid plasmid loss than that detected in the negative control, expressing no Par proteins. These observations indicate that the IncC-derived ParA and ParB proteins together stabilize the *parS*-bearing minimal SGI1 replicon and the 053 protein is not involved in this process.

### Sci protein affects regulation of the IncC *parAB053* operon

Expression levels of Par systems are fine-tuned and even small changes can cause plasmid destabilization, thus, we hypothesized that the Sci protein exerts its IncC-destabilizing effect by influencing expression of the *parAB053* operon. Therefore, we cloned the promoter region of *parA* (P*_parA_*) into a β-galactosidase test plasmid and measured its expression level in the presence of Type 1 (R16a) and Type 2 (R55^ΔTn^) IncC plasmids, which provided the ParAB053 proteins in *trans*. Sci protein was expressed from the IGR placed in p15A-based vectors with appropriate resistance markers. P*_parA_* acted as a strong promoter in the absence of IncC plasmids, and its activity was not directly modified by Sci (Figure 8C). In contrast, the presence of IncC plasmids (R55^ΔTn^ or R16a) caused approximately 11- or 8-fold reduction in the promoter activity, respectively. Surprisingly, the presence of Sci significantly reduced the level of repression brought about by IncC plasmids, as the promoter activity decreased by only 1.5–1.7-fold compared to the unrepressed state (no IncC plasmid), indicating that the Sci protein somehow alleviates repression of the *parAB053* operon.

A similar assay was carried out using a minimal IncC plasmid assembled from the *rep* region and the entire *parAB053* operon with its own promoter region, which provided Par proteins. Surprisingly, the minimal replicon did not reach the repression level achieved by entire IncC plasmids and the Sci protein did not modify the strength of repression. In the next assay, ParAB053 proteins were expressed from a single-copy vector under the control of the arabinose-inducible P*_araBAD_* promoter, depriving the *par* operon of its own regulation. After induction with 1% l-arabinose, the strength of repression was similar to that obtained with the minimal IncC replicon, and Sci had no detectable effect on the repression (Figure 8D). Summarizing these observations we conclude that autoregulation of the *parAB053* operon (presumably by ParB) is modulated by Sci, however, this intervention does not occur through a direct effect on ParAB053 proteins. The fact that maximal repression was only achieved when the full IncC plasmids were present suggests that the proteins expressed from the *parAB053* operon are not sufficient to tightly regulate the P*_parA_* promoter and that an additional regulatory factor is required, which is missing from the minimal IncC plasmid and certainly encoded on the IncC backbone, but outside the *par* operon. Our results also indicate that this additional regulator may be the target of Sci, as similar levels of repression were observed in the presence of Sci and the entire IncC plasmids expressing the putative regulator (Figure 8C, R55^ΔTn^/R16a + Sci), and in the absence of the IncC backbone, independently of Sci (Figure 8C, miniIncC and Figure 8D).

### SGI1 stability is enhanced by its incompatibility factors in the presence of R55

The data we gathered suggested that the *parS* sites of SGI1 have a dual role, contributing to both destabilization of IncC plasmids and to stabilization of SGI1 itself, by serving as ParB binding sites for partitioning, whereas Sci appears to be involved only in IncC destabilization via disrupting regulation of the *parAB053* operon. To examine the effects of the *parS* sites and/or the Sci protein on SGI1 stability and its coexistence with an IncC plasmid, KO mutations were generated. We knocked out the *parS* sites by introducing the same 4-bp insertion into DRs of SGI1-C that was previously applied in the DR2** mutant plasmid pGMY49 (Figure 3B) and EMSA probe (Figure 5I), while the Sci protein was eliminated by deletion of IGR_ORF2 (*sci* gene). Stability of cohabitation of SGI1 KO mutants and R55^ΔTn^ was assayed in approx. 22-generations of growth without antibiotic selection, and the proportions of cells retaining one or both elements was determined. Although the output of such assays is significantly biased by the stochastic constitution of the cell populations, since the plasmid can destabilize SGI1 (21) and vice versa (38,36,18), and cells that retain or lose one or both components show different growth rates, our results clearly demonstrated that both DR1 KO and *sci* deletion caused severe destabilization of SGI1, while DR2 KO had a similar, but much weaker, effect on its stability (Figure 8E). Compared to wt SGI1-C, DR2 KO slightly decreased the proportion of the otherwise predominant ‘SGI1-only’ cells and increased that of ‘R55-only’ and ‘R55 + SGI1’ cells to approximately 4%. In contrast, DR1 KO and *sci* deletion radically reduced SGI1 stability, leading to populations predominated by ‘R55-only’ cells. In these cultures, the second largest subpopulation was cells with both elements retained (6–7%), while ‘SGI1-only’ cells were almost (DR1 KO) or entirely (Δ*sci*) absent. Similar distributions were observed with the double and triple mutants, however, the ‘SGI1 + R55^’^ fraction remained considerable in both cases. These data indicate that lack of *parS* sites and the Sci protein not only facilitates the co-existence of SGI1 and R55^ΔTn^, but also significantly destabilizes SGI1.

Nevertheless, the difference in the stability between DR1 and DR2 KO mutant SGI1-Cs was bewilderingly large. Since *parS* sites in DRs differed by only a single, non-conserved position (Figure 5A), which had no effect on ParB binding (Figure 5D, F), we presumed that the difference in stability was caused by the locations of the DRs, rather than by their sequence divergence. It has previously been observed that replication-deficient SGI1 mutants show similarly high instability in the presence of R55^ΔTn^ (18). Since DR1 is located close to the promoter of the *S004-repA* operon, which is responsible for replication and copy-number control of excised SGI1, we supposed that the KO mutation negatively affects the regulation of SGI1 replication. Therefore, we conducted an RT-qPCR assay to assess copy numbers of wt SGI1-C, Δ*sci*, DR1 KO and DR2 KO mutants in the presence of R55^ΔTn^ (Figure 8F). The results showed that DR2 KO and Δ*sci* did not significantly effect SGI1 copy number, while confirming that DR1 KO reduced it to approx. one copy per cell. These findings are consistent with our previous observation that this low copy number is accompanied by extreme instability of SGI1 (18), and also indicate that integrity of the *parS* site in DR1 is necessary for the normal replication, and consequently for the stability, of SGI1.

## Discussion

Conjugative plasmids and IEs are key factors in the distribution of genes conferring antibiotic and heavy metal resistance, as well as numerous other adaptive traits, among diverse host bacteria. Plasmids and IEs apply similar mechanisms to guarantee their stable maintenance, such as partitioning and post-segregational killing. Unlike plasmids, IEs ensure their vertical transfer by integration into the host chromosome, however, for horizontal transfer they have to excise, relinquishing this quiescent state, which can lead to sibling cells that have lost the element.

SGI1-family IMEs that are mobilized by IncA and IncC plasmids apply several refined mechanisms to reduce the risk of being lost when they coexist with the helper plasmid, which triggers their excision leading to segregation (21,24,36,18,37). Besides the TA system (24), another important function involved in SGI1 stabilization is the transient plasmid-like replication of the excised element (18). Further, SGI1 destabilizes the helper plasmid, which also contributes to its own stable maintenance. There is strong evidence that SGI1 replication both enhances its persistence in cells where the island coexists with the IncC helper plasmid, and appears is a key factor in plasmid destabilization. Incompatibility between SGI1 and its mobilizing IncC and IncA plasmids has been reported (38,36,18,68,39), and it is proposed that SgaDC, the SGI1-encoded master regulator may have a central role in this phenomenon. Although the exact mechanism of plasmid expulsion is unknown, interference with plasmid maintenance functions (replication, partitioning) has been suggested. Several possible ways have been proposed, such as titration of the host replication protein, DnaA, or perturbation of plasmid partitioning or post-segregational killing mechanisms, mediated by an unknown factor, the amount of which is increased by the elevated copy number of replicating SGI1 (36,18,37).

In this work, we present evidence that SGI1 intervenes in plasmid maintenance via two distinct factors. Deletion mapping and stability assays (Figures 1–3) revealed that the 31-bp direct repeats, DR1 and DR2, located upstream of ORFs S004 and *traH_S_*, respectively, and the IGR between ORFs S014 and S015, comprise elements responsible for incompatibility with IncC plasmids. One copy of the 31-bp DRs (DR2) and the IGR fragment, without any other SGI1-derived regions, exerted strong incompatibility with R55^ΔTn^ when both were present in either *cis* or *trans* in ∼15 copies. The joined DR2-IGR fragment resulted in a much lower level of IncC plasmid loss when present on a single copy plasmid, while it led to extremely rapid plasmid segregation in a high-copy state (>300 per cell) compared with that mediated in the 15-copy state (Figure 4A). Analysis of the incompatibility exerted by the two elements separately indicated that DR2-promoted plasmid loss was robustly copy-number dependent, while that of the IGR was not (Figure 4B, D), suggesting that the two elements act via different mechanisms. Furthermore, DR2 and IGR amplified the effects of one another as they induced much weaker incompatibility alone than together (Figure 4).

In the 5′-part of the DR1 and DR2 repeats, a 22-bp IR motif was identified, with striking similarity to an imperfect IR downstream of the putative –10 promoter box preceding the *parAB053* operon in the IncC plasmid, R55 (Figure 5AB). ParABS systems are generally autoregulated by ParA, ParB or both via binding to a *parS*-like sequence close to the promoter of the *parABS* operon (45) and their *parS* sites are often consists of one or more IR motifs. No *parS* sites have yet been identified in the IncC *parABS* system, however, the third ORF, *ORF053*, is necessary for stable plasmid maintenance, and it was previously hypothesized that the *parS* site may be within *ORF053* (51). Our findings, demonstrating that SGI1 carries two copies of an IR motif, which are involved in incompatibility with R55 and resemble the IR present in the promoter region of the *parAB053* operon, raise the possibility that these IRs act as *parS* sites for IncC plasmids, through which they can generate centromere-mediated incompatibility. A thorough sequence search revealed that R55 contains 13 additional IR motifs scattered through its conserved backbone that are similar to those found in the P*_parA_* promoter and in SGI1. Most of these IRs are located in intergenic regions near R55_13, R55_14, R55_24/*parB*-like regulator, R55_29, *parAB053*, R55_55, R55_74, R55_109, R55_170/DNA helicase and R55_174/*eexC* genes, while four copies are within the ORFs, R55_6, R55_08, R55_91 and R55_130/*xerC*. Alignment of the two SGI1- and 14 IncC-derived IRs showed a fully conserved symmetrical 14-bp core sequence flanked by less conserved AAAC and GTTT motifs (Figure 5A–C). EMSA experiments with three selected putative *parS* sites (one representing the consensus of the 16 IR sequences and also identical to that present in DR1 of SGI1, the others from DR2 of SGI1 and the promoter region of R55 *parAB053* operon) proved that these motifs are binding sites for the ParB protein expressed by the 2nd ORF of *parAB053* operon (Figure 5D–F). Interestingly, the fully conserved 14-bp palindromic core sequence, TTTCACATGTGAAA, was not bound by ParB (Figure 5I) indicating that the flanking AAAC and GTTT motifs, and particularly the highly conserved GT bases in the right side flanking sequence, are important for ParB recognition. ParB of IncC plasmids contains a ParB and a KorB domain (27). The prototype KorB protein, KorB encoded by the IncPα plasmid RP4 acts as ParB in the partitioning system of this plasmid, and also as a global regulator of housekeeping genes. A helix-turn-helix (HTH) motif was predicted in KorB, responsible for binding to 12 palindromic 13-bp operator sequences (69,70), similarly to other KorB homologs and ParB-family members (71). KorB can form tetramers in solution (72) and a C-terminal dimerization domain has been identified (73). Although similar details are not available for IncC ParB, structural modelling using SwissModel suggested that it also has an HTH near its N-terminus. Further, EMSAs with ParB gave unusually highly shifted bands with the three *parS* probes (Figure 5D–F), suggesting that IncC ParB behaves similarly to several other ParB proteins that also cause high shifts and have been shown to spread from the *parS* site along the DNA (74,75), potentially forming higher order complexes. A 4-bp insertion in the centre of the *parS* site in DR2 abolished ParB binding, indicating that ParB may bind to the IR of *parS* as a symmetric dimer or tetramer form. The same 4-bp insertion or removal of half of the *parS* site in DR2 eliminated incompatibility with R55^ΔTn^ (Figure 3B, C), supporting this hypothesis and indicating that ParB-*parS* binding is required for SGI1-mediated plasmid destabilization.

A Genbank search with the 22-bp *parS* sequence present in the P*_parA_* promoter revealed that the majority of IncC plasmids carry 12–14 copies of the conserved 14-bp core sequence of the *parS* sites, several plasmids have 9 copies (i.e. CP050727, CP029123 and CP050727) and plasmid pV266-a (LC056472) carries only 7 copies. IncA plasmids identified to date (76) contain 10–14 copies and no IncA or IncC plasmids were found to have more than 14 or less than 7 copies, except pRAx (5 copies), a deletion derivative of pRA1, which has 14 copies (77). Interestingly, several plasmids not belonging to the IncA or IncC groups but sharing some parts of their backbone (i.e. LC501639, CP040595 and KX832927) also contain 5–6 copies of IncC *parS*. Many strains of *Proteus*, *Salmonella*, *Klebsiella*, *E. coli*, *Shewanella* and *Morganella* carry two copies of IncC *parS* sequence in their chromosome, mostly within integrated SGI1-family elements, however, *Providencia stuartii* FDAARGOS_1040 and *Vibrio cholerae* SA3G contain 3 and 4 chromosomal copies that do not appear to be associated with SGI1-related elements.

Helper-induced transient replication of SGI1 is established as a major factor in IncC plasmid destabilization (36,18,37,68). Our data demonstrate that increased *parS* copy number accelerates plasmid loss (Figure 4B, D), consistent with a model where helper-plasmid-induced replication leads to higher SGI1 copy number (with two *parS* sites per copy), thus providing 14–16 extra *parS* copies, comparable to the number of *parS* sites in the plasmid itself, and this increased number of *parS* sites contributes to plasmid destabilization. The dose-dependence of incompatibility can explain why SGI1 replication appears to be a key factor in plasmid elimination. As Par systems are sensitive to such perturbations, doubling of *parS* site numbers will impair normal plasmid partitioning (78), likely due to ParB protein titration, or competition for attachment sites or other partitioning proteins (41,45). Thus, the effects of SGI1-derived *parS* sites on IncC stability are similar to, and can be treated as, a type of centromere-mediated incompatibility (45).

Nevertheless, introduction of approx. 15 extra *parS* copies led to much weaker incompatibility than that observed with the full wt SGI1 (compare Figure 4B and Figure 1B), indicating that *parS* sites are just one factor contributing to incompatibility. Deletion mapping revealed that a second factor is expressed from the IGR between ORFs S014 and S015. After ruling out the role of IGR_ORF1 and a putative small RNA overlapping IGR_ORF2, a 51 aa (5.9 kDa) protein named Sci was identified as the *parS*-mediated incompatibility-enhancing factor. Sci encoded by IGR_ORF2 appears to be a simple helical protein with a C-terminal 13 aa disordered tract (Supplementary Figure S10), which seems unimportant for its function (pJKI1137, Figure 6B). Sci alone exerted weak incompatibility, which was slightly increased when expressed from a high-copy plasmid (Figure 4B, D), however, Sci significantly enhanced the incompatibility exerted by the DR2-derived *parS* site when expressed from its own promoter or the strong P*_cat_* promoter (Figures 4C and 7C).

Removal of the mobilizing helper plasmid, which triggers excision from the chromosome and hereby causes SGI1 destabilization, appears to be an adaptive strategy for IMEs like SGI1, to ensure their stable maintenance. Further, IMEs require the helper elements for their horizontal distribution, hence, transient stabilizing mechanisms are needed during periods of coexistence with their helpers. ICEs face similar challenges when they excise before conjugal transfer and exist in an extrachromosomal state, despite not being destabilized by other elements in this context. Plasmid-like replication is an important stabilizing mechanism for IEs (44,18), but cannot provide sufficient stability, as demonstrated for locked-out Δ*int* mutant pSGI1 (36) or SXT/R391 elements (44). Thus, further stabilization mechanisms are applied. Transient expression of a *parMRC* system, *srpMRC*, under the control of the FlhDC-family activator, SetDC, significantly contributes to stable maintenance of SXT-family elements. This means of expression control also ensures that the *srpMRC* system functions only when the ICE is excised and replicates as a plasmid. Although the occurrence of Par systems in ICEs does not appear to be unusual (44,43), similar systems in IMEs have not previously been identified. Similar to SXT, SGI1 also requires transient stabilization when in an extrachromosomal state due to helper-induced excision (21,24,18). Although SGI1 and its relatives do not include a complete Par system, they do contain functional *parS* sites, mimicking those of IncA and IncC plasmids, by which this issue can be solved by exploiting the Par system of the plasmids. Here we demonstrate that the otherwise unstable SGI1-derived minimal replicon carrying a *parS* site located in DR2 became significantly more stable if R55 *parAB053* was expressed in *trans* (Figure 8A). This stabilizing effect was manifested only if both ParA and ParB were expressed (Figure 8B), while 053 protein did not contribute to the effect. Consistently, KO mutation of *parS* sites in SGI1-C significantly reduced SGI1 stability alongside coresident R55^ΔTn^ under nonselective conditions (Figure 8E). Together, these data strongly suggest that SGI1 uses *parS* sites to both expel helper plasmids and hijack the plasmid-encoded partitioning system to increase its own stability through active partitioning.

While the function of SGI1-borne *parS* sites is relatively clear, the mode of action of Sci protein in incompatibility and SGI1 stability is rather puzzling. The fact that the incompatibility exerted by a *parS* site was significantly amplified by Sci expression (Figures 4C and 7C) suggests that Sci also influences plasmid partitioning, for example, via modulating regulation of the *parAB053* operon or ParB-*parS* binding. Expression of the *parAB053* operon from a minimal IncC replicon reduced expression from P*_parA_* (Figure 8C). Since the –10 box of the P*_parA_* promoter overlaps with the *parS* site to which ParB binds, we conclude that ParB is a repressor of its own synthesis, as established in many ParABS systems (45,79,80), however, Sci had no effect on expression from P*_parA_*. in this minimal system A similar result was obtained when ParAB053 proteins were expressed from the inducible P*_araBAD_* promoter (Figure 8D), which released the operon from its original regulation, ruling out feedback effects between the β-galactosidase measuring plasmid and the ParAB053-producer plasmid. In contrast, when an entire IncC plasmid was present, stronger repression was observed, which was clearly alleviated by Sci (Figure 8C). These findings indicate that correct regulation of P*_parA_* involves an additional IncC-plasmid-derived factor, encoded outside the *parAB053* operon, and Sci presumably exerts its effect on this putative regulator. This effect may occur, for example, via competition of Sci with the unknown regulator for its binding site. However, direct DNA binding by small helical protein such as Sci, which lacks any known domains, seems unlikely. A more realistic hypothesis is that Sci acts via protein–protein interactions by reducing the binding specificity or strength of the regulator to its target DNA, or through interfering with other activities of this factor involved in *par*-regulation. Anyway, if Sci releases strict repression of the otherwise strong P*_parA_* promoter, the increased expression of ParB can easily destabilize the single-copy helper plasmid. Plasmid destabilization by overexpression of the cognate ParB or ParR proteins has been described (81–83), and was also observed in our experimental system, where the minimal SGI1 replicon, *rep*_SGI1_, could be stabilized only if ParAB053 expression was induced by very low (0.001%) l-arabinose concentration, while stronger induction always led to rapid loss of the SGI1 replicon. Nevertheless, under natural conditions, elevated ParB expression due to the effects of Sci intervention on a single-copy IncC plasmid, may be optimal to increase the partitioning efficiency of the 7–8 copies of free plasmid-like SGI1, further enhancing SGI1 stability. This model is consistent with the observation that Δ*sci* mutant SGI1-C stability was clearly decreased under nonselective conditions compared to wt SGI1-C (Figure 8E). In light of these results, we favour the hypothesis that Sci disrupts control of the *parAB053* operon by influencing a yet unknown co-repressor of the P*_parA_* promoter, leading to elevated expression of Par proteins, which assists in SGI1 partitioning, while simultaneously perturbing that of the IncC plasmid. However, regulation of the IncC ParABS system requires further exploration to facilitate better understanding of these interactions. Therefore, investigations to examine *parAB053* operon control mechanisms and search for the Sci protein target are in progress.

Inactivation of SGI1-C *parS* sites decreased its stability, however, the segregation rate of the two DR KO mutants and their effect on the stability of the coresident IncC plasmid under nonselective conditions proved unexpectedly different (Figure 8E). The 4-bp insertion introduced into the *parS* site of DR2 moderately decreased SGI1 retention and increased the fraction of R55-only and SGI1 + R55 cells relative to wt SGI1-C. In contrast, the same mutation in DR1 caused a dramatic change in SGI1 stability, as the proportion of SGI1-only cells dropped to 1.7%, while R55-only cells dominated the population, and the ratio of R55 + SGI1 cells also exceeded 7%. The low stability of DR1 KO mutant SGI1-C can be explained by its reduced copy number (approx. 1/cell, Figure 8F), as observed in *rep* mutants (18). This change in copy number indicates that *parS* insertion mutation in DR1 impairs normal replication control of SGI1. The facts that *parS* in DR1 is 25 bp upstream of the core AcaCD-binding motif of the P*_S004_* promoter and that ParB cannot bind KO mutant *parS* (Figure 5I) strongly suggest that ParB somehow participates in regulation of the *S004-repA* operon. A similar genetic constitution is found in the promoter region of the *traG_S_H_S_* operon, where the *parS* site is only 10 bp upstream of the AcaCD binding site, however, expression of the *traG_S_H_S_* operon presumably does not influence replication. Thus, DR2 KO mutant stability may be changed solely because of the loss of one copy of *parS*, while the copy number in this mutant remained unaffected (Figure 8E, F). Interestingly, an analogous constitution occurs in the promoter regions of four AcaCD-regulated operons in the conserved IncC backbone (30). In these promoters (the first ORFs of the operons are R55_9/vcrx012, R55_55/vcrx036, HS904_RS00445/vcrx068 and *traF*) similar crosstalk can occur as that observed for P*_S004_* of SGI1. Since ParB often acts as global regulator of various genes involved in the maintenance functions of several plasmids (84), this may also be the case for ParB and IncC plasmids. Thus, investigations on the presumed multiple roles of ParB are ongoing.

SGI1 appears to efficiently exploit helper plasmid features via an intricate control circuit elucidated in recent studies (21,36,18,37). The first signal is likely the IncC-entry-induced SOS response, which triggers expression of the SGI1-encoded activator, SgaDC (68). Although SgaDC is constitutively expressed (35) from quiescent SGI1, the amount produced is insufficient to induce SGI1 excision and replication. When the plasmid enters a cell containing SGI1 in its chromosome, it encounters a basal level of SgaDC, which likely quickly increases, due to SOS induction. This may activate expression of AcaB, the activator of the plasmid *acr-acaCD* operon (32). The *acaB* gene is part of the AcaCD regulon and its promoter, like all other AcaCD-responsive promoters, is also activated by SgaDC (37). Induction of the *acr-acaCD* operon leads to elevated levels of the AcaCD activator, which induces expression of genes encoding the plasmid-borne conjugation apparatus, as well as contributing to triggering SGI1 excision (30,21) and replication (18), however, SgaDC appears to have major role in the latter processes (18,37). Due to the positive feedback loop between *acaB* and *acr-acaCD* and SOS-mediated induction of *sgaDC*, the concentrations of both activators will be sufficient for SGI1 excision and generation of 7–8 copies/cell. Meanwhile, all AcaCD-controlled conjugation genes encoded by the helper plasmid and SGI1 (*traN_S_*, and *traG_S_H_S_*) are also induced. Hence, everything is in place for the lateral transfer of SGI1, however, retention of the island is riskier in this state. To prevent SGI1-free sibling cells arising, the higher copy number of SGI1 achieved by transient plasmid-like replication, together with expression of the TA system, increase SGI1 stability, however these phenomena appear insufficient over evolutionary time scales, as SGI1 applies additional protective measures. Hijacking the *parABS* system of IncC helper plasmids using ‘stolen’ *parS* sites and a protein factor modification of expression from the *parAB053* operon is an elegant ‘two birds with one stone’ solution. Expelling the helper plasmid and increasing SGI1 stability by active partitioning is a logical way to solve the stability issue. SGI1, which bears two *parS* sites, may be more efficiently dispersed during cell division using *parABS* system proteins of the helper. Simultaneously, this upsets the partitioning of the helper plasmid by competing for ParB, ParA, attachment sites, or other partitioning factors, and through the effects of the Sci protein. In this way, SGI1 negates the need for a self-encoded Par system and schedules its partitioning and incompatibility as required.

SgaDC is considered a key element in IncC plasmid destabilization (36–38,68,39) and is indispensable for induction of SGI1 replication and maintenance of 7–8 copies per cell. Here we demonstrate that the presence of increased amounts of identified incompatibility factors due to SGI1 replication strengthens its incompatibility with R55. Thus, one can conclude that SgaDC may act only indirectly on incompatibility via its effect on replication control. Consistent with this model, no destabilization of the IncC plasmid, pRMH760, by SGI1-K variant lacking the *sgaDC* operon, due to a deletion from within *traN_S_* (S005) to within S009, was observed (38,39), despite it possessing both *parS* sites and the *sci* gene. Further, *sgaDC*-deleted mutants cannot replicate normally (36,18,37), therefore, the weak or undetectable incompatibility of SGI1-K can be explained by its low copy number, and consequent lower amount of Sci protein and fewer additional *parS* copies (Figure 8E, F), as was also observed with our replication deficient deletion mutants (Δ1–Δ5, Figure 1). Although we did not detect similarly strong incompatibility of an SgaDC-expression vector as recently reported (39), we also observed that expression of SgaDC in *trans* and the presence of the entire IncN2/N3-related replicon (including one *parS* site) on an approximately 15-copy plasmid caused low-level but detectable R55^ΔTn^ loss (Figure 2C, D), even though RepA expression itself exerted no detectable incompatibility. These observations require further investigation, but we believe that the incompatibility is not primarily caused by components of SGI1 replication machinery themselves (RepA, S004, SgaDC), but rather by reinforcement of the effects of incompatibility factors due to the effect of increased SGI1 copy number following its normal replication.

Hence, SGI1 takes advantage of IncC (and possibly IncA) plasmids in several ways, utilizing the plasmid AcaCD-dependent regulatory systems and transfer apparatus. Here we report an additional form of parasitism on IncC plasmids by SGI1 via exploitation and disruption of their partitioning system. Although elements from several other IME-families are also mobilized by IncC plasmids (30,85), the fact that SGI1-family elements are far more common suggests that this complex behaviour is a winning evolutionary strategy.

## Supplementary Material

gkae050_Supplemental_File

## Data Availability

All data is contained within the manuscript and/or the supplementary materials.

## Notes

Present address: Gábor Murányi, Institute of Experimental Medicine, Budapest, H1083 Hungary.
