# Supplementary material for: Two birds with one stone: SGI1 can stabilize itself and expel the IncC helper by hijacking the plasmid parABS system
Source: Nucleic Acids Res. 2024 Feb 1;52(5):2498–518. doi: 10.1093/nar/gkae050 (PMC10954446; doi:10.1093/nar/gkae050)
Supplement: gkae050_Supplemental_File [file gkae050_supplemental_file.pdf]

Additional Materials and Methods and Supplementary data

Content:

Supplementary Figure S1
Supplementary Figure S2
Supplementary Figure S3
Supplementary Figure S4
Supplementary Figure S5
Supplementary Figure S6
Supplementary Figure S7
Supplementary Figure S8
Supplementary Figure S9
Supplementary Figure S10
Supplementary Figure S11

Supplermentary Dataset 1

Supplementary Table S1
Supplementary Table S2
Supplementary Table S3
Supplementary Table S4

Supplementary Text S1
Supplementary References

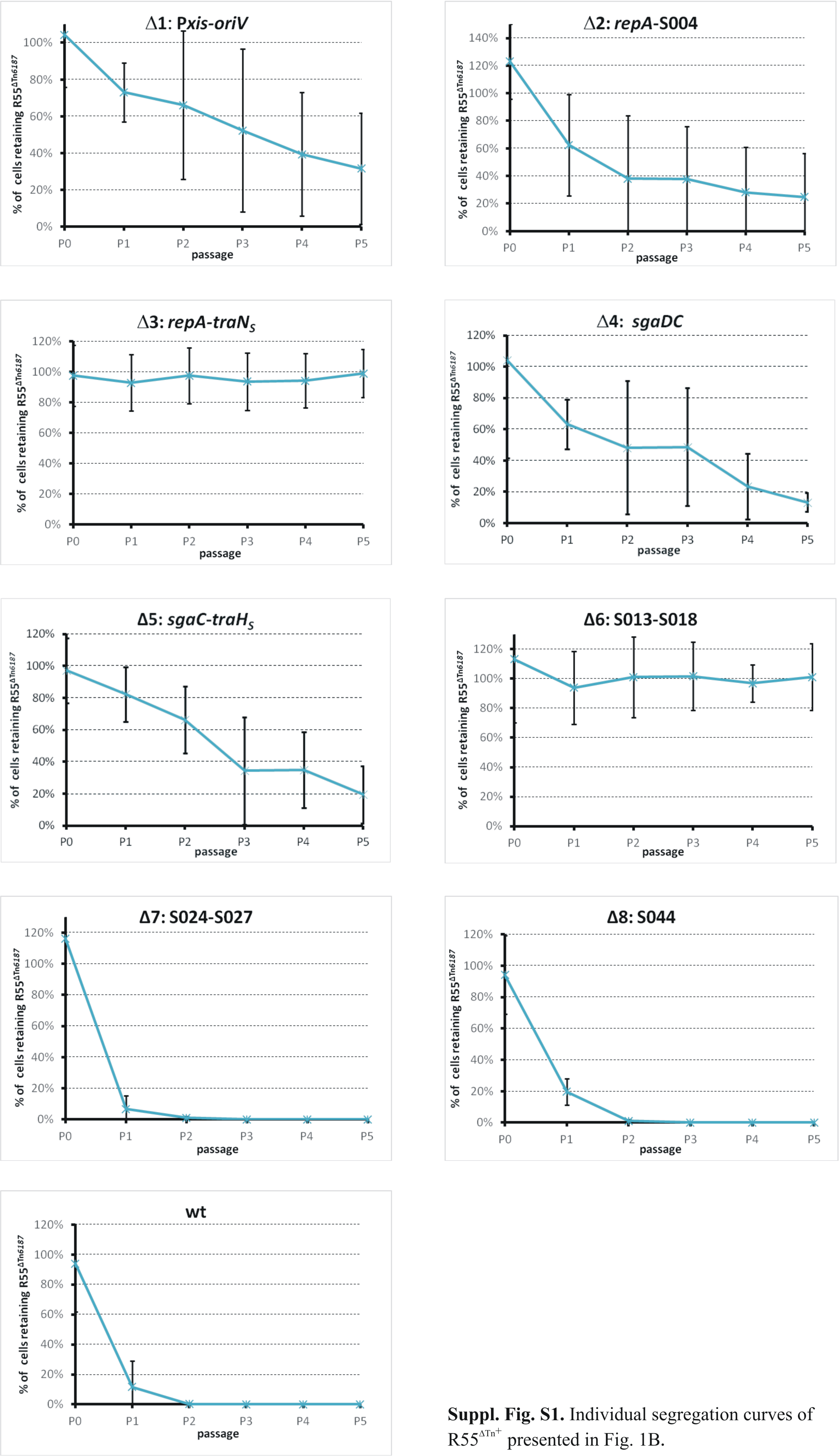

Suppl. Fig. S1. Individual segregation curves of R55$^{\Delta Tn^{+}}$ presented in Fig. 1B.

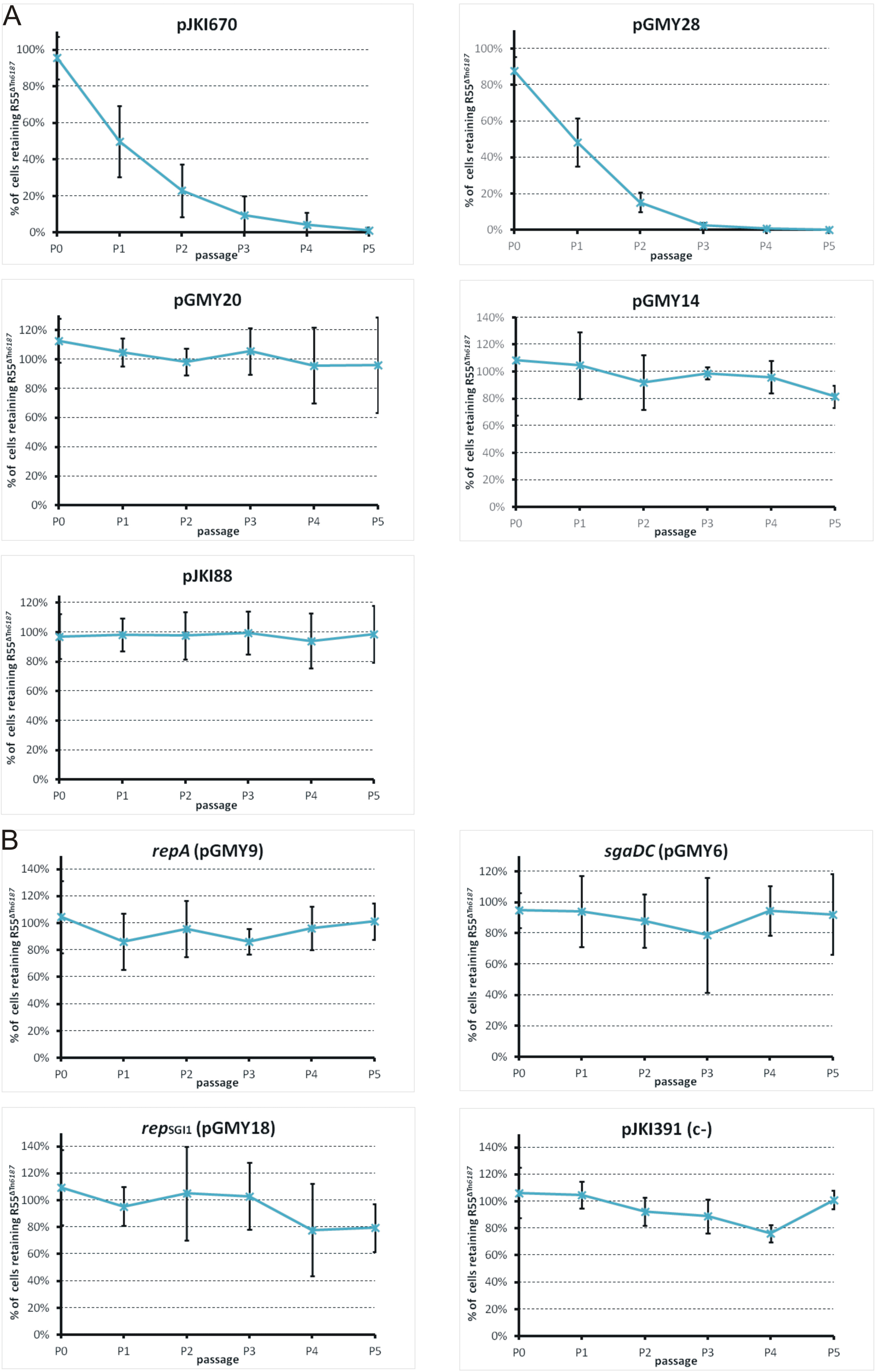

Suppl. Fig. S2. Individual segregation curves of R55$^{\Delta Tn^{+}}$ presented in Fig. 2B (A) and Fig. 2C (B).

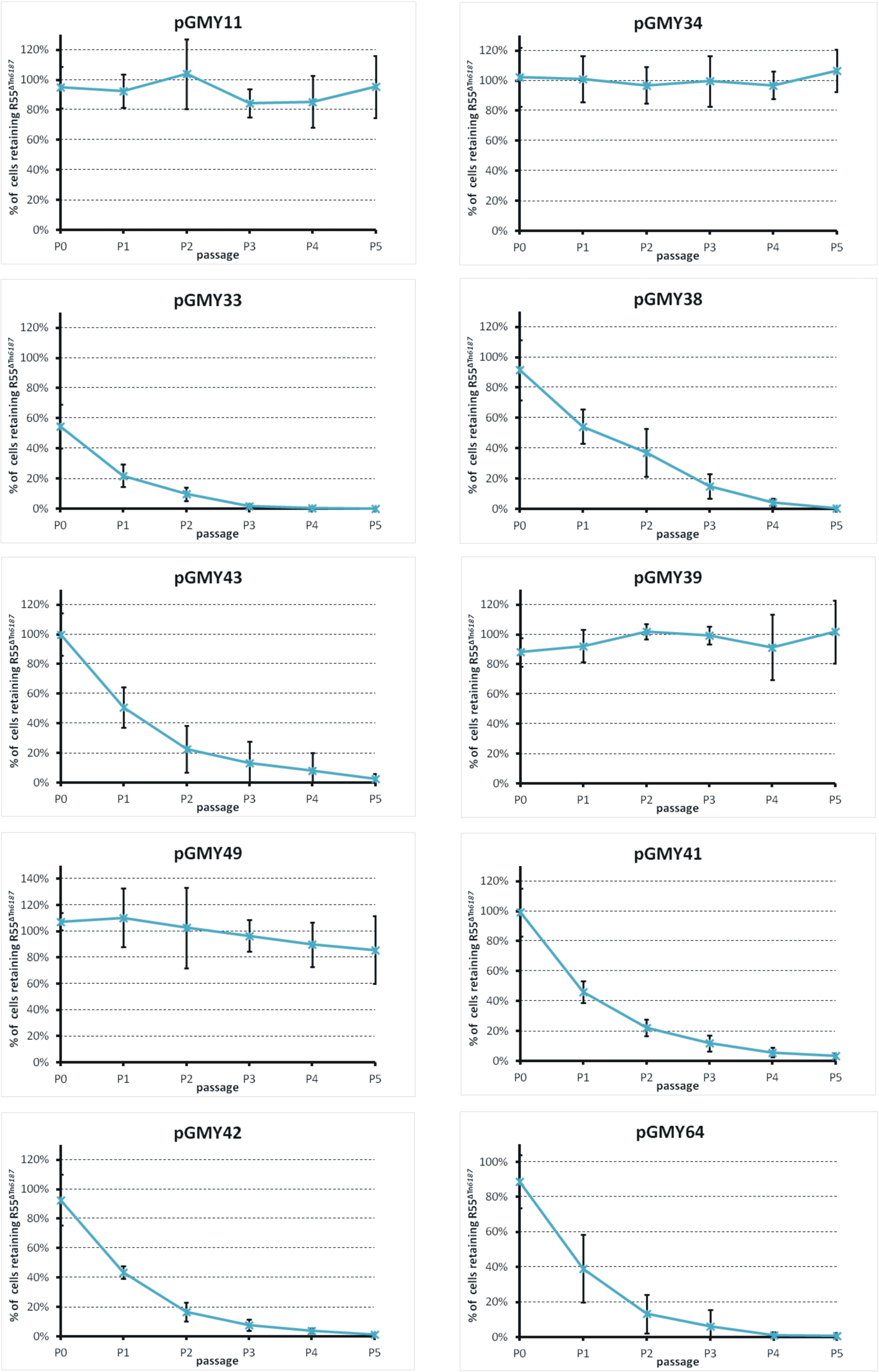

Suppl. Fig. S3. Individual segregation curves of R55$^{\Delta Tn^{+}}$ presented in Fig. 3AB.

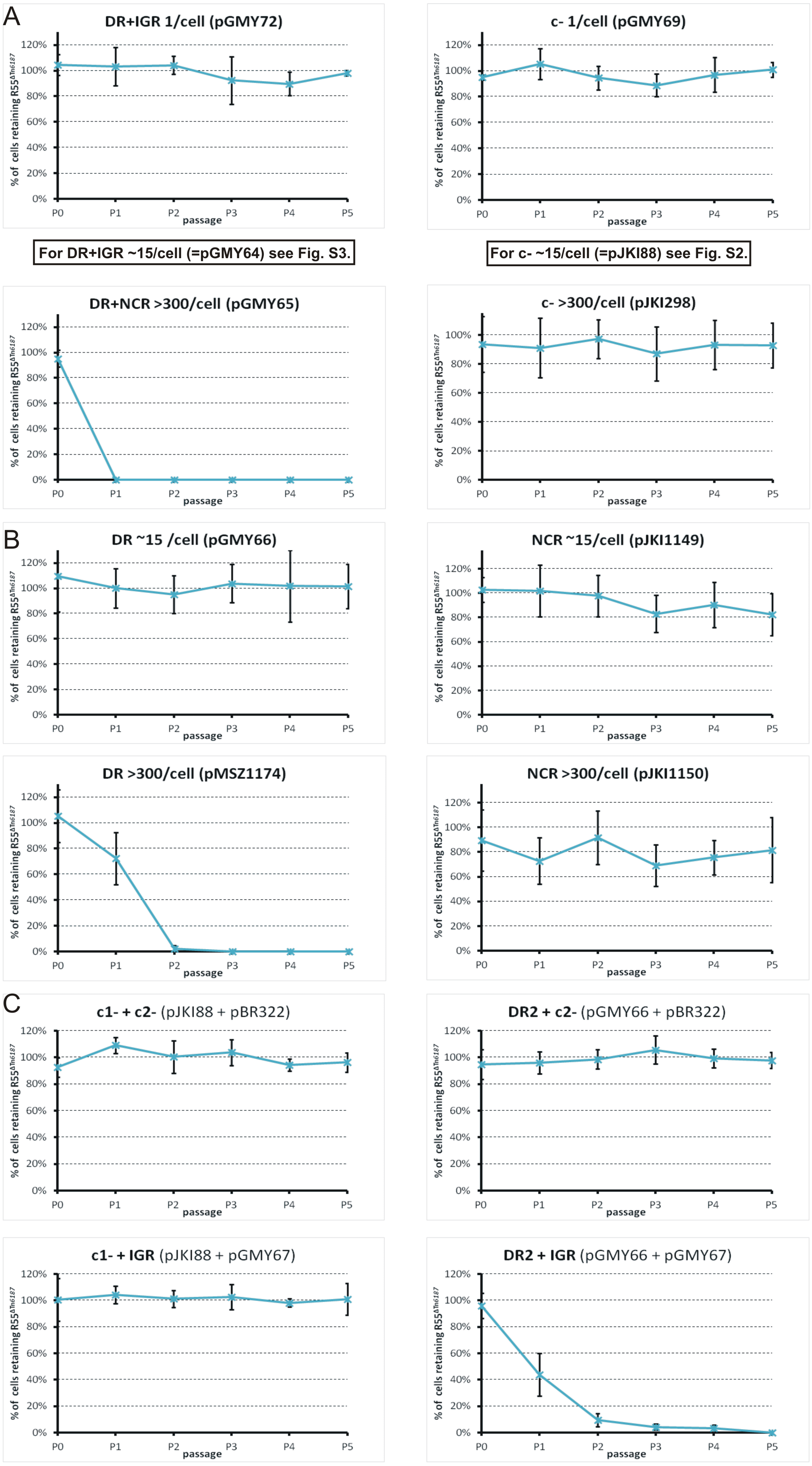

Suppl. Fig. S4. Individual segregation curves of R55$^{\Delta Tn^{+}}$ presented in Fig. 4A-C and the negative controls for Fig. 4A..

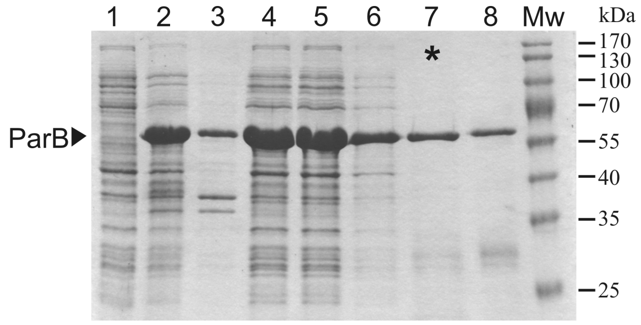

Suppl. Fig. S5. Purification of ParB protein. Samples from various stages of the purification were separated on 12.5% denaturing polyacrylamide gel. Lane 1: total cell extract from Tuner (DE3), lane 2: total cell extract from Tuner (DE3)/pMSZ1166 induced with 0.3 mM IPTG; lane 3: pellet and lane 4: cleared lysate (supernatant) of IPTG-induced, sonicated (lysed) and centrifuged Tuner (DE3)/pMSZ1166 cells; lane 5: unbound fraction of the cleared lysate; lane 6: flow-through of washing solution, lane 7: eluted ParB protein fraction; lane 8: non-eluable protein fraction bound to the affinity matrix. Mw: SM0671 protein ladder (Thermo Fisher Scientific). Asterisk indicates the ParB fraction used for EMSA experiments.

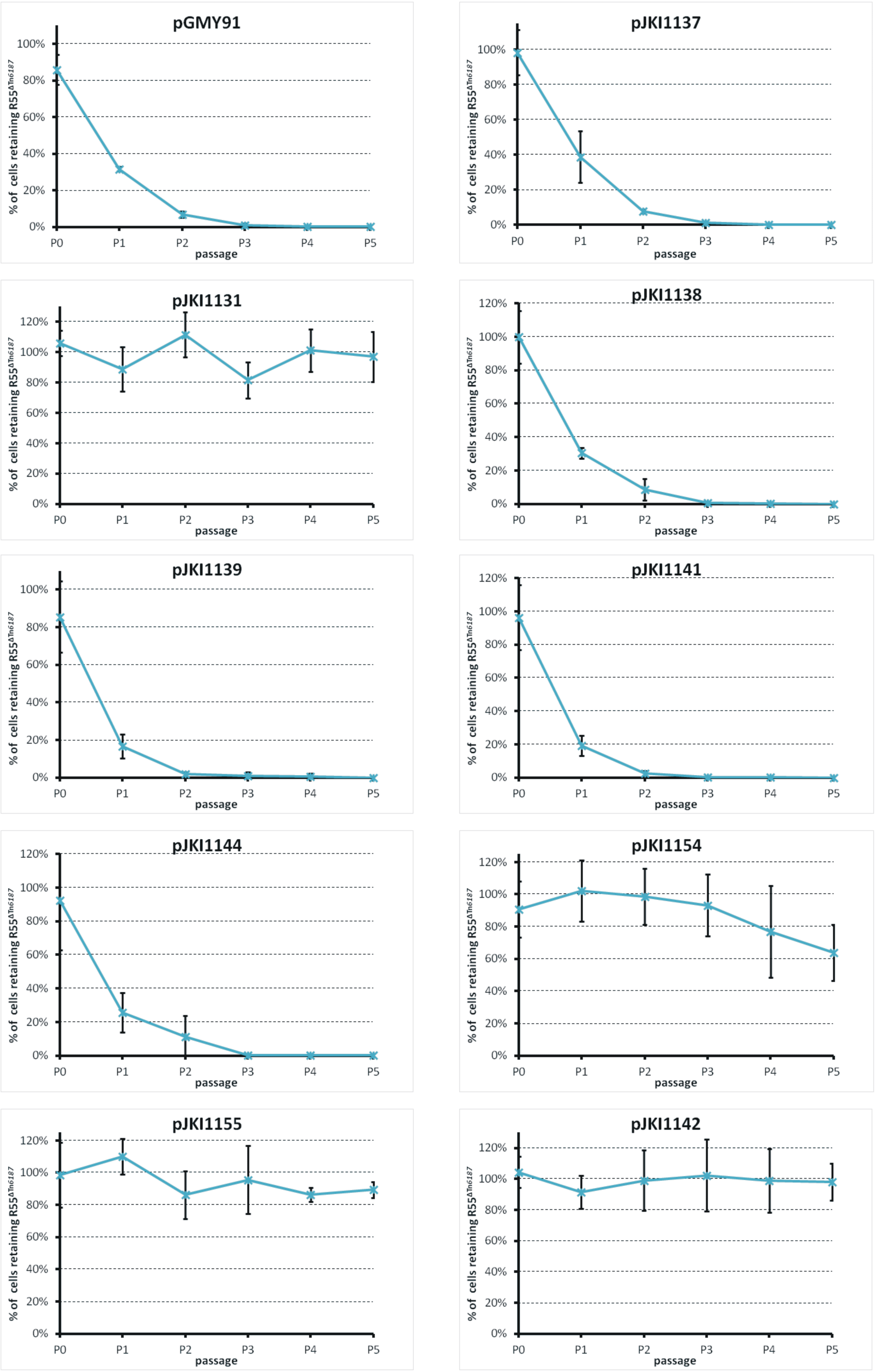

Suppl. Fig. S6. Individual segregation curves of R55$^{\Delta Tn+}$ presented in Fig. 6BC.

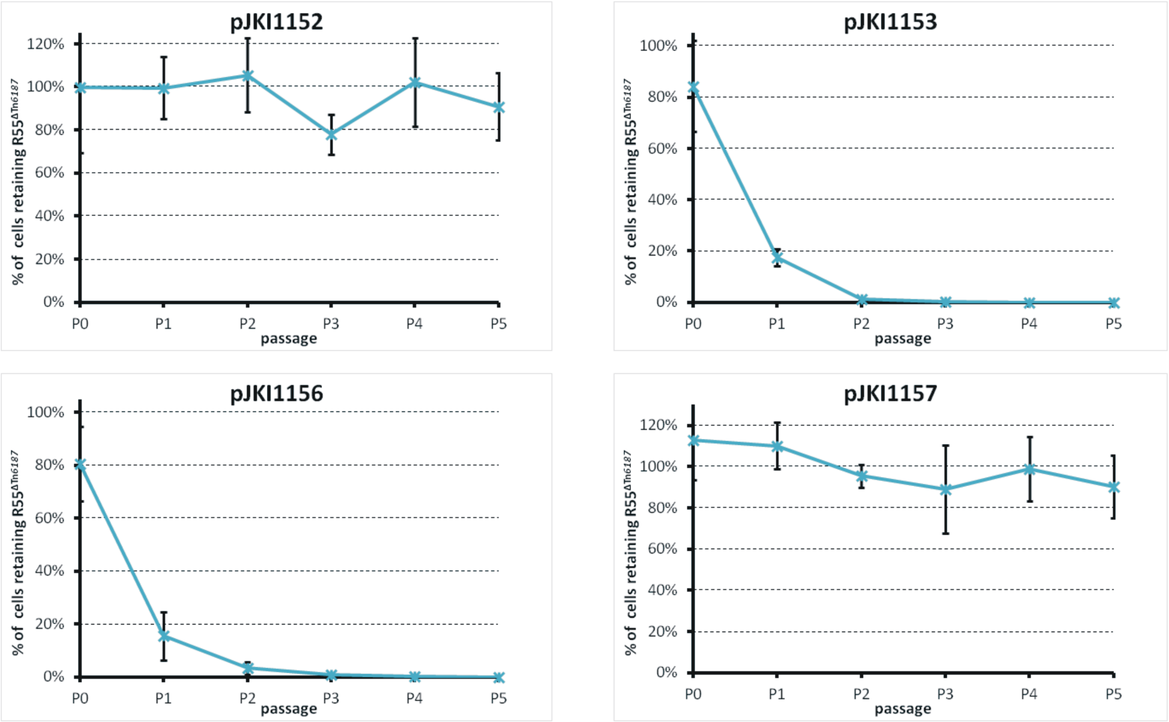

Suppl. Fig. S7. Individual segregation curves of R55$^{\Delta Tn+}$ presented in Fig. 6EFG.

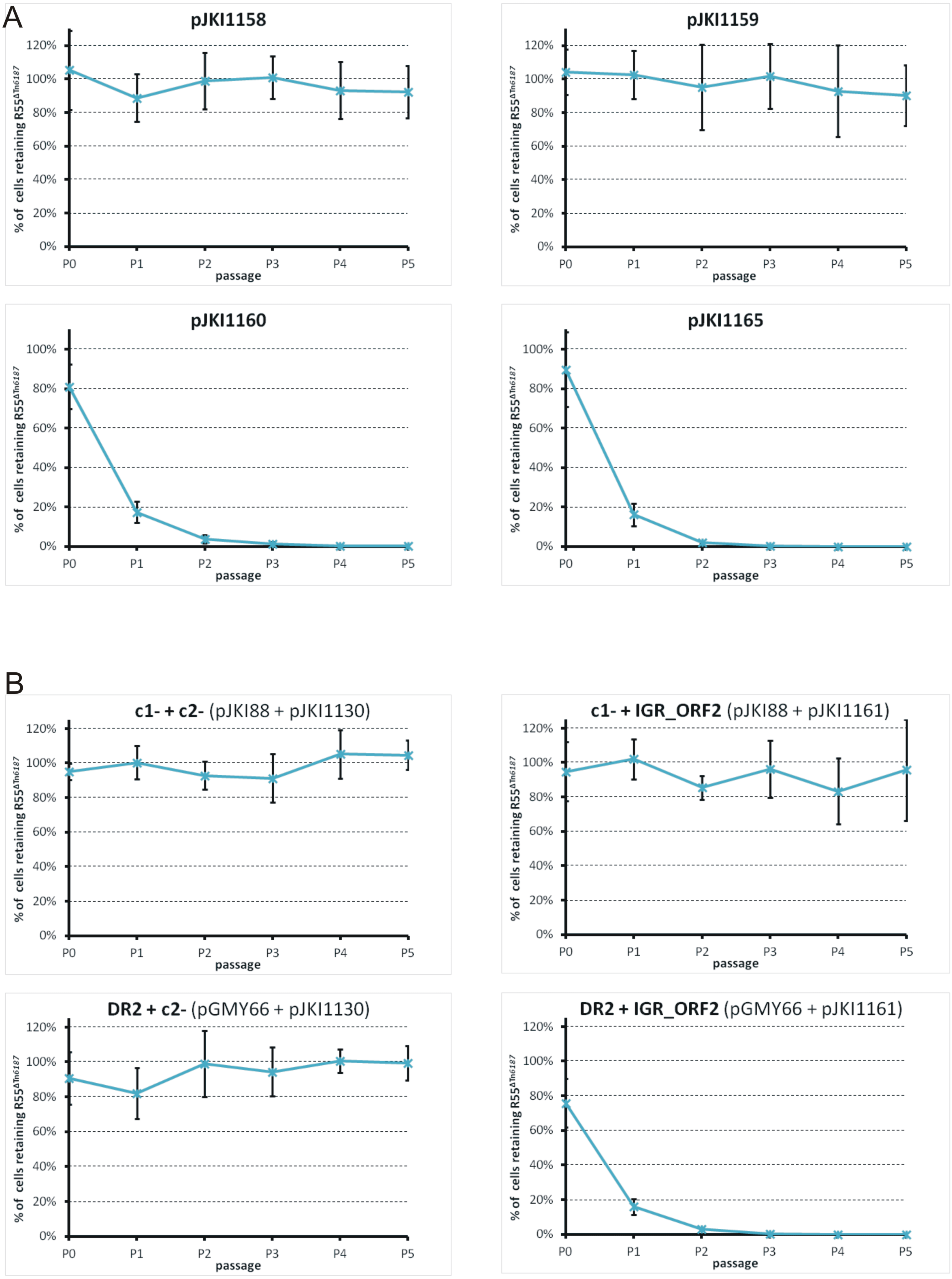

Suppl. Fig. S8. Individual segregation curves of R55$^{\Delta Tn^{+}}$ presented in Fig. 7A (A) and Fig. 7B (B).

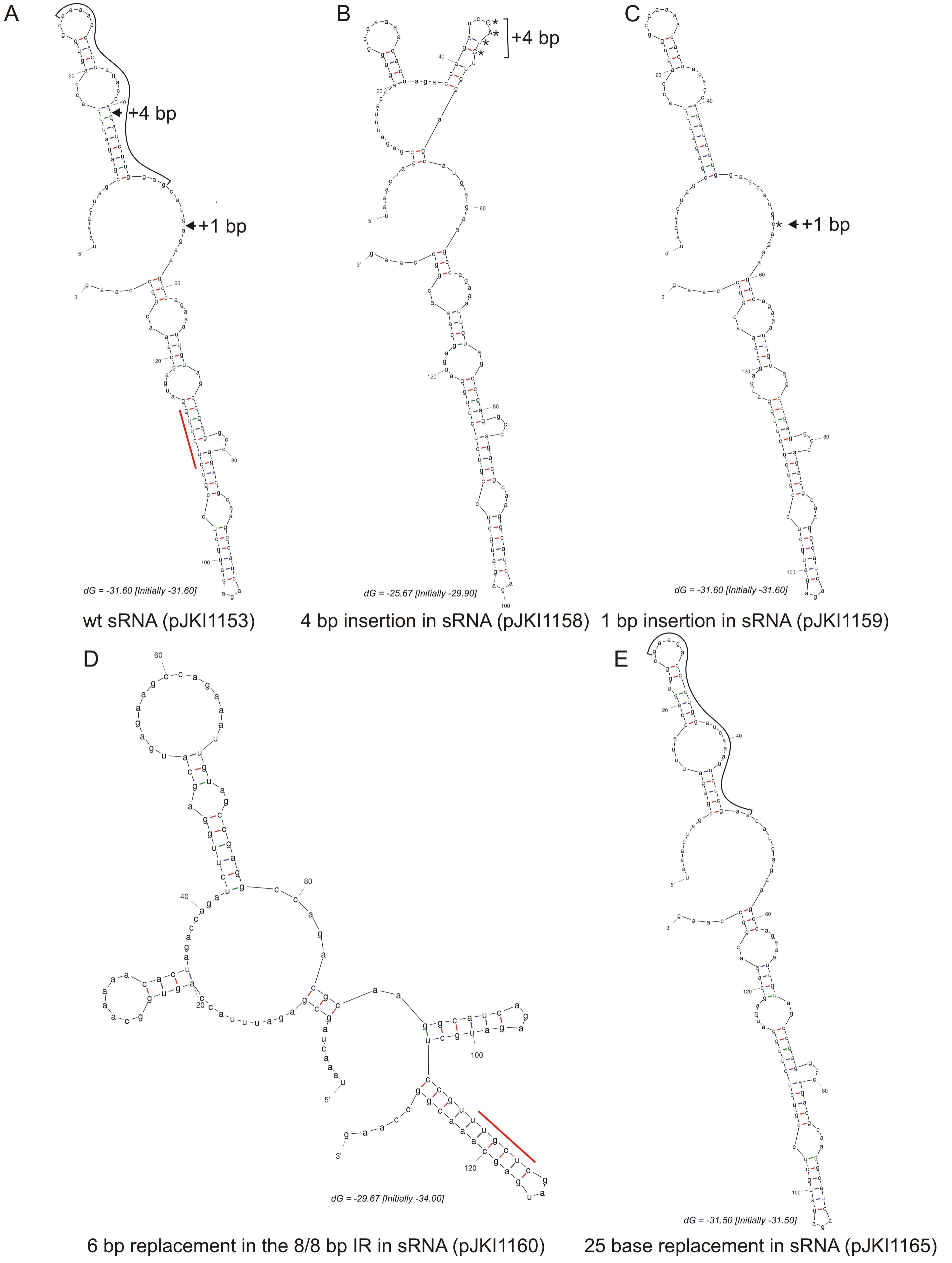

Suppl. Fig. S9. Secondary structure predictions for the putative wt and mutated small RNAs expressed from IGR_ORF2. (A) The putative wt RNA expressed from pJKI1153. Arrows indicate positions where 4 or 1 bp insertions were introduced (panels B and C). Red line indicates the Leu codons exchanged in panel D. Black curve shows the region where codons were replaced by samesense codons in panel E. (B) Secondary structure prediction for the 4-bp insertion (asterisks) mutant putative small RNA. The insertion was obtained by filling in the *Bgl*II site of pJKI1153. (C) Secondary structure prediction for the 1-bp insertion mutant putative small RNA obtained by inserting a single C base (asterisk) after the 34th bp position of IGR_ORF2. (D) Secondary structure prediction for the putative small RNA in which the Leu(30) and Leu(31) codons of IGR_ORF2 are exchanged (red line). This mutation destroys the left arm of the 8/8-bp IR motif located in the 3' part of IGR_ORF2. (E) Secondary structure prediction for the putative small RNA, in which the 2-10 codons of IGR_ORF2 are replaced with samesense codons (black curve).

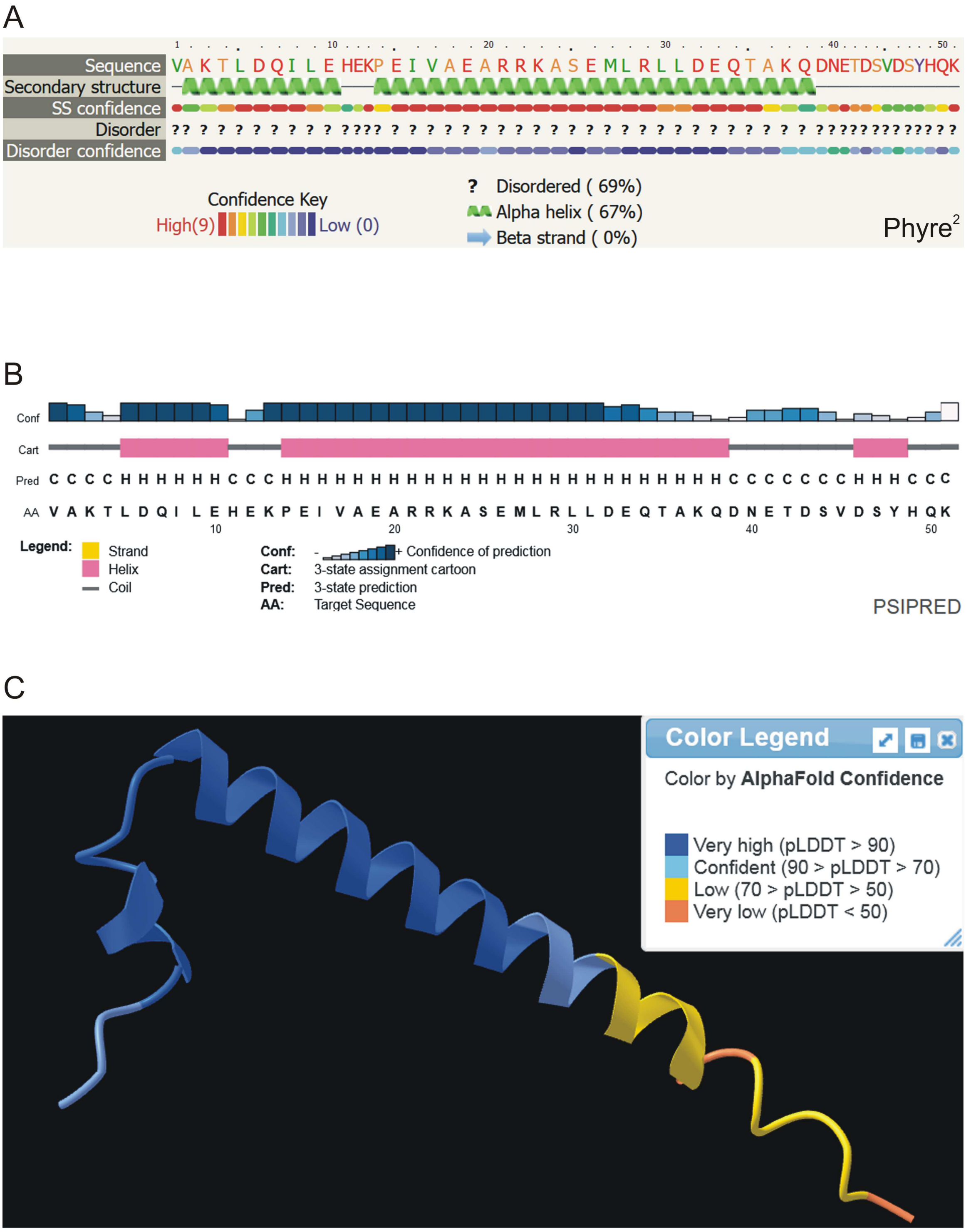

Suppl. Fig. S10. Structure predictions for the 51 aa Sci protein encoded by IGR_ORF2. (A) Phyre^2 prediction (B) PSIPRED prediction. (C) AlphaFold model.

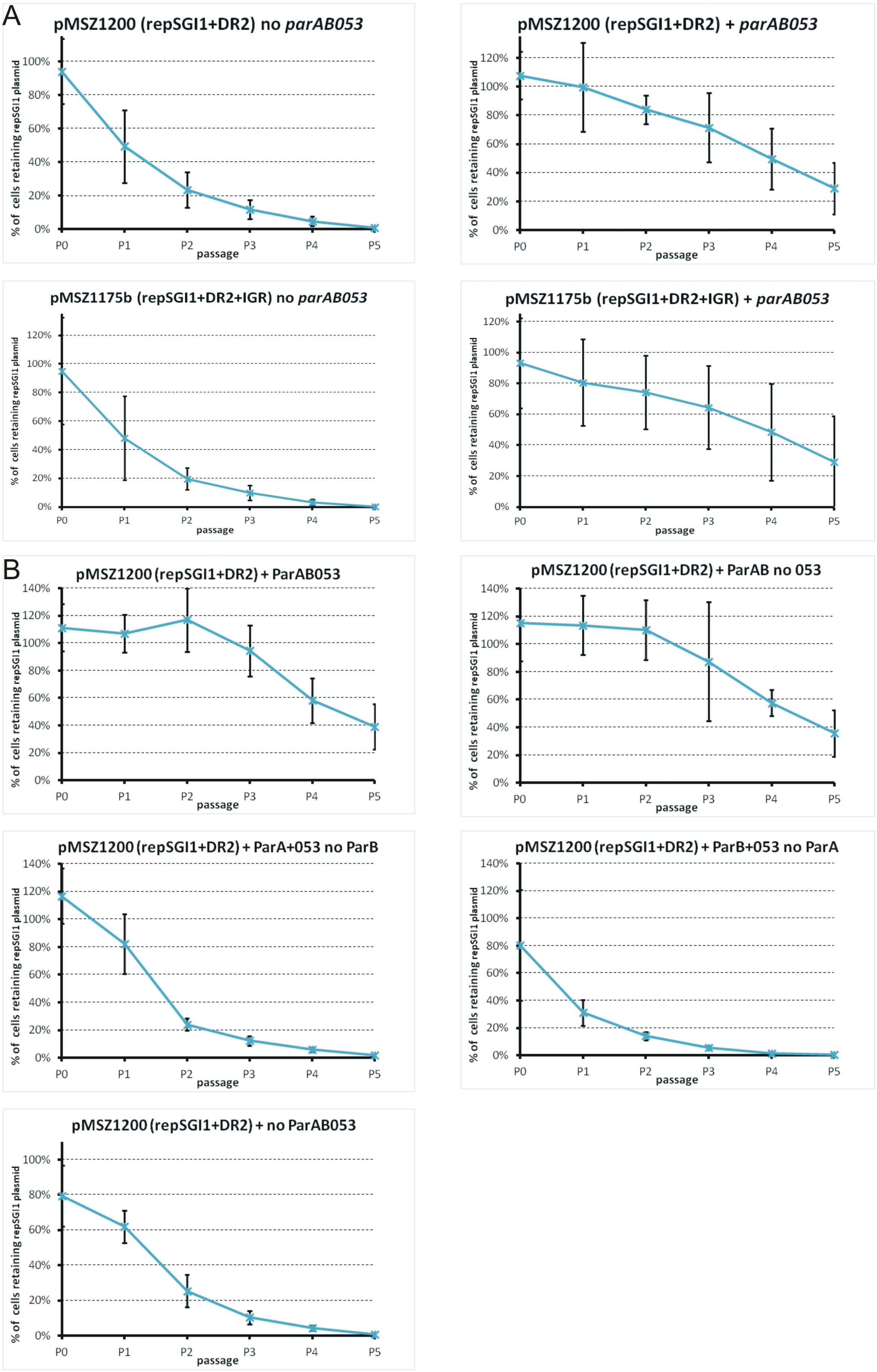

Suppl. Fig. S11. Individual segregation curves of SGI1-derived minimal replicons presented in Fig. 8AB. Rep$_{SGI1}$+DR2 - pMSZ1200, rep$_{SGI1}$+DR2+IGR - pMSZ1175b, no *parAB053* - pJKI625, *parAB053* (ParA$^+$B$^+$053$^+$) - pJKI1128, *parA$^-$B$^+$053$^+$* (ParB+053, no ParA) - pMSZ1263, *parA$^+$B$^-$053$^+$* (ParA+053, no ParB) - pMSZ1272, *parA$^+$B$^+$053$^-$* (ParAB no 053) - pGMY86.

Suppl. Dataset 1. Summary of the statistical analysis (see below for graphic representations)

Reference	No. of replicates	Half life (95% CI)	Sample	No. of replicates	Half life (95% CI)	Sample/Ref (95% CI)	Significant
A							
pJKI670 (c+)	44	1.00 (0.76-1.24)	pGMY28	4	0.80 (0.59-1.00)	0.79 (0.51-1.07)	No
			pGMY33	8	0.46 (0.27-0.65)	0.46 (0.24-0.67)	Yes
			pGMY38	16	1.30 (1.01-1.59)	1.30 (0.88-1.72)	No
			pGMY41	8	0.92 (0.68-1.16)	0.91 (0.59-1.24)	No
			pGMY42	8	0.80 (0.58-1.02)	0.79 (0.50-1.08)	No
			pGMY43	8	1.00 (0.75-1.25)	1.00 (0.65-1.34)	No
			pGMY64	40	0.68 (0.48-0.89)	0.68 (0.42-0.94)	Yes
B							
pGMY38	16	1.30 (1.01-1.59)	pGMY41	8	0.92 (0.68-1.16)	0.70 (0.46-0.95)	Yes
			pGMY42	8	0.80 (0.58-1.02)	0.61 (0.40-0.83)	Yes
C							
pGMY64	40	0.68 (0.48-0.89)	pGMY38	16	1.30 (1.01-1.59)	1.91 (1.19-2.62)	Yes
			DR2+IGR *in trans*	4	0.72 (0.51-0.92)	1.05 (0.62-1.49)	No
			pGMY91	4	0.56 (0.37-0.75)	0.82 (0.45-1.19)	No
			pJKI1137	8	0.64 (0.45-0.83)	0.94 (0.54-1.34)	No
			pJKI1138	4	0.56 (0.37-0.74)	0.81 (0.45-1.18)	No
			pJKI1141	4	0.40 (0.22-0.57)	0.58 (0.27-0.89)	Yes
			pJKI1144	8	0.51 (0.32-0.70)	0.75 (0.39-1.11)	No
			pJKI1153	8	0.37 (0.19-0.54)	0.54 (0.23-0.84)	Yes
			DR2+IGR_ORF2 *in trans*	4	0.35 (0.17-0.53)	0.51 (0.20-0.82)	Yes
D							
pJKI1153	8	0.37 (0.19-0.54)	pJKI1144	8	0.51 (0.32-0.70)	1.40 (0.55-2.25)	No
			pJKI1156	8	0.34 (0.16-0.53)	0.93 (0.26-1.61)	No
			pJKI1160	8	0.37 (0.19-0.56)	1.02 (0.32-1.72)	No
			pJKI1165	4	0.35 (0.17-0.53)	0.95 (0.28-1.62)	No

Half life - Virtual passage number associated with 50% plasmid loss; CI – confidence interval, Sample/Ref – ratio of the sample/reference half life parameter values, with 95 confidence limits. The estimated plasmid loss is regarded as significantly different from the reference value, when the ratio of the half-life parameters is significantly different from 1 (i.e the confidence interval of the ratio does not contain 1). Lower half life means faster plasmid loss and stronger incompatibility.

A

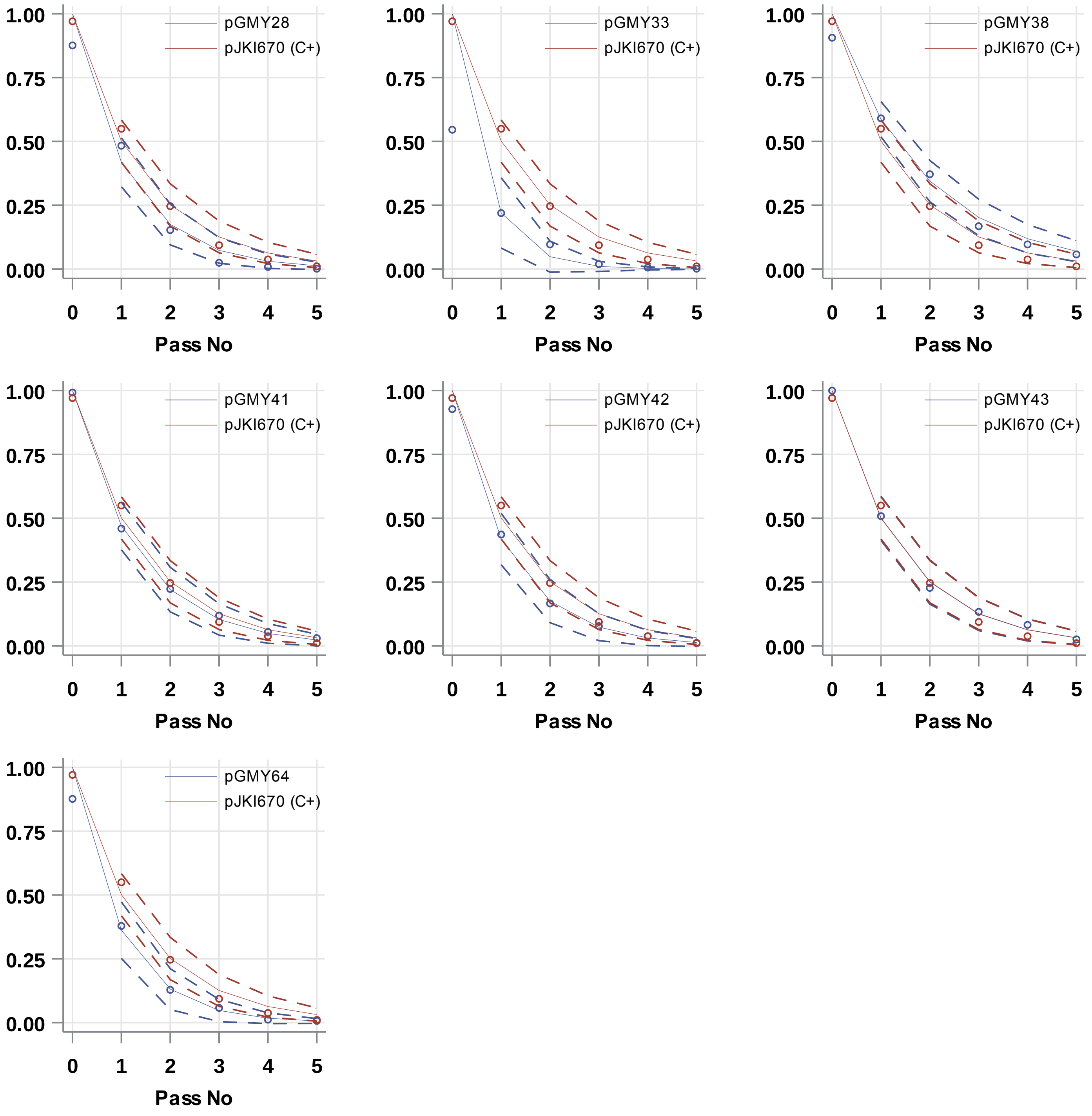
pGMY28
pJKI670 (C+)
pGMY33
pJKI670 (C+)
pGMY38
pJKI670 (C+)
pGMY41
pJKI670 (C+)
pGMY42
pJKI670 (C+)
pGMY43
pJKI670 (C+)
pGMY64
pJKI670 (C+)
1.00
0.75
0.50
0.25
0.00
0 1 2 3 4 5
Pass No

B

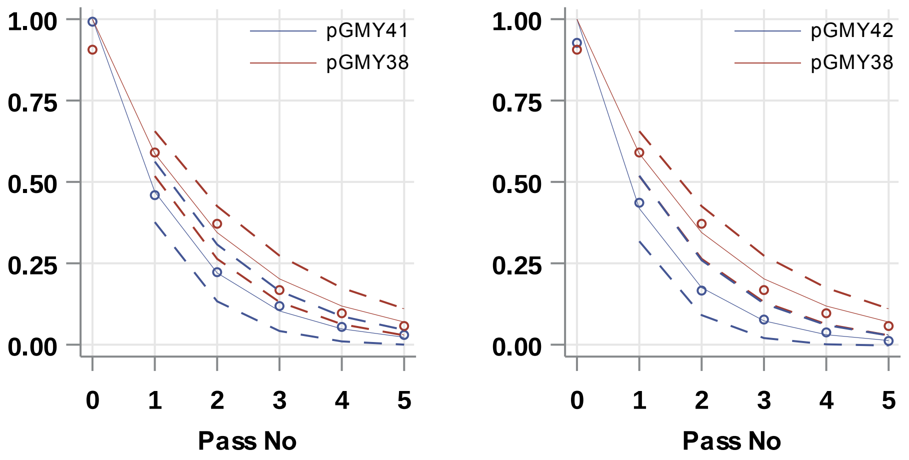
pGMY41
pGMY38
pGMY42
pGMY38
1.00
0.75
0.50
0.25
0.00
0 1 2 3 4 5
Pass No

C

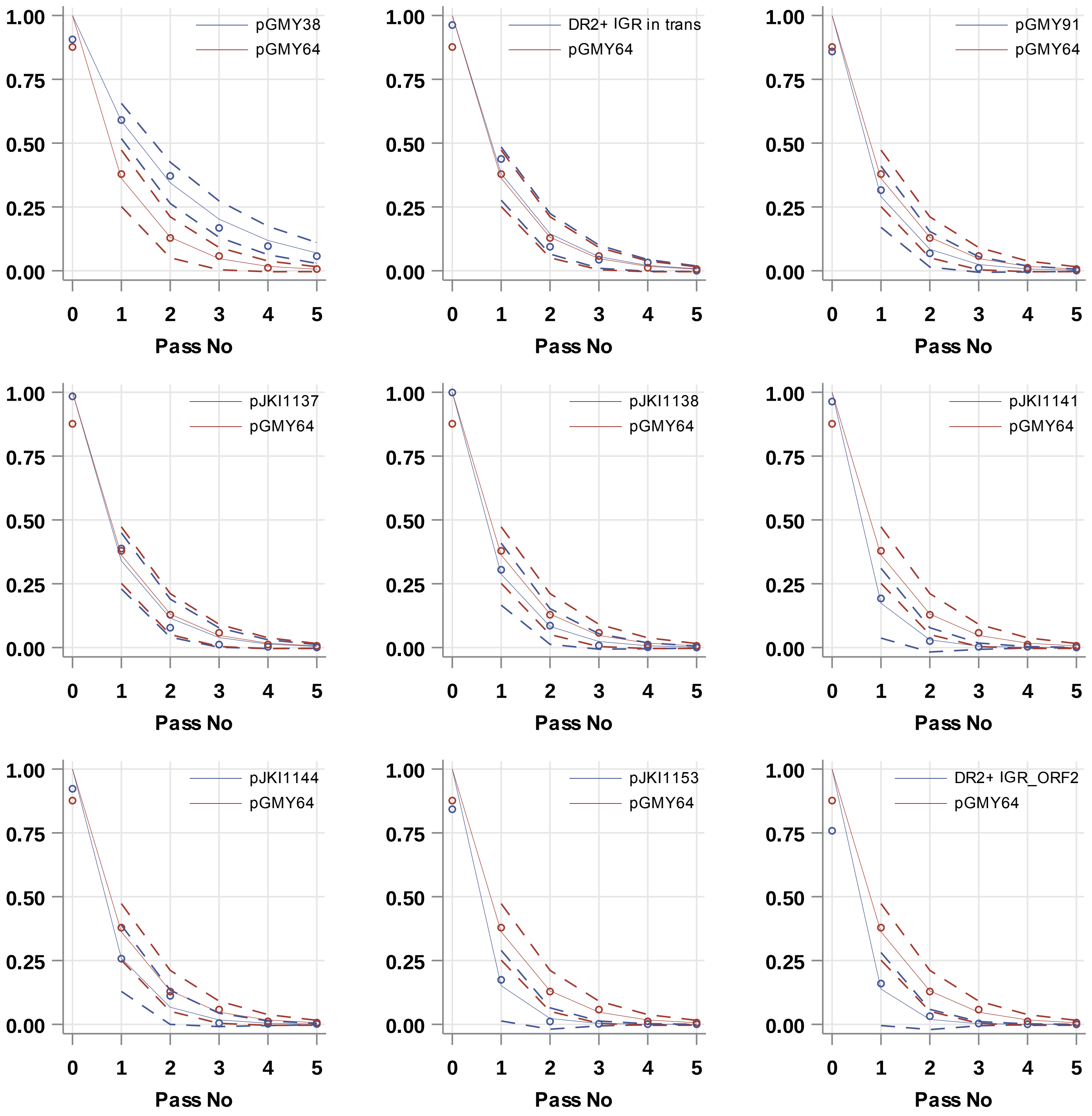
pGMY38
pGMY64
DR2+ IGR in trans
pGMY64
pGMY91
pGMY64
pJKI1137
pGMY64
pJKI1138
pGMY64
pJKI1141
pGMY64
pJKI1144
pGMY64
pJKI1153
pGMY64
DR2+ IGR_ORF2
pGMY64
1.00
0.75
0.50
0.25
0.00
0 1 2 3 4 5
Pass No

D

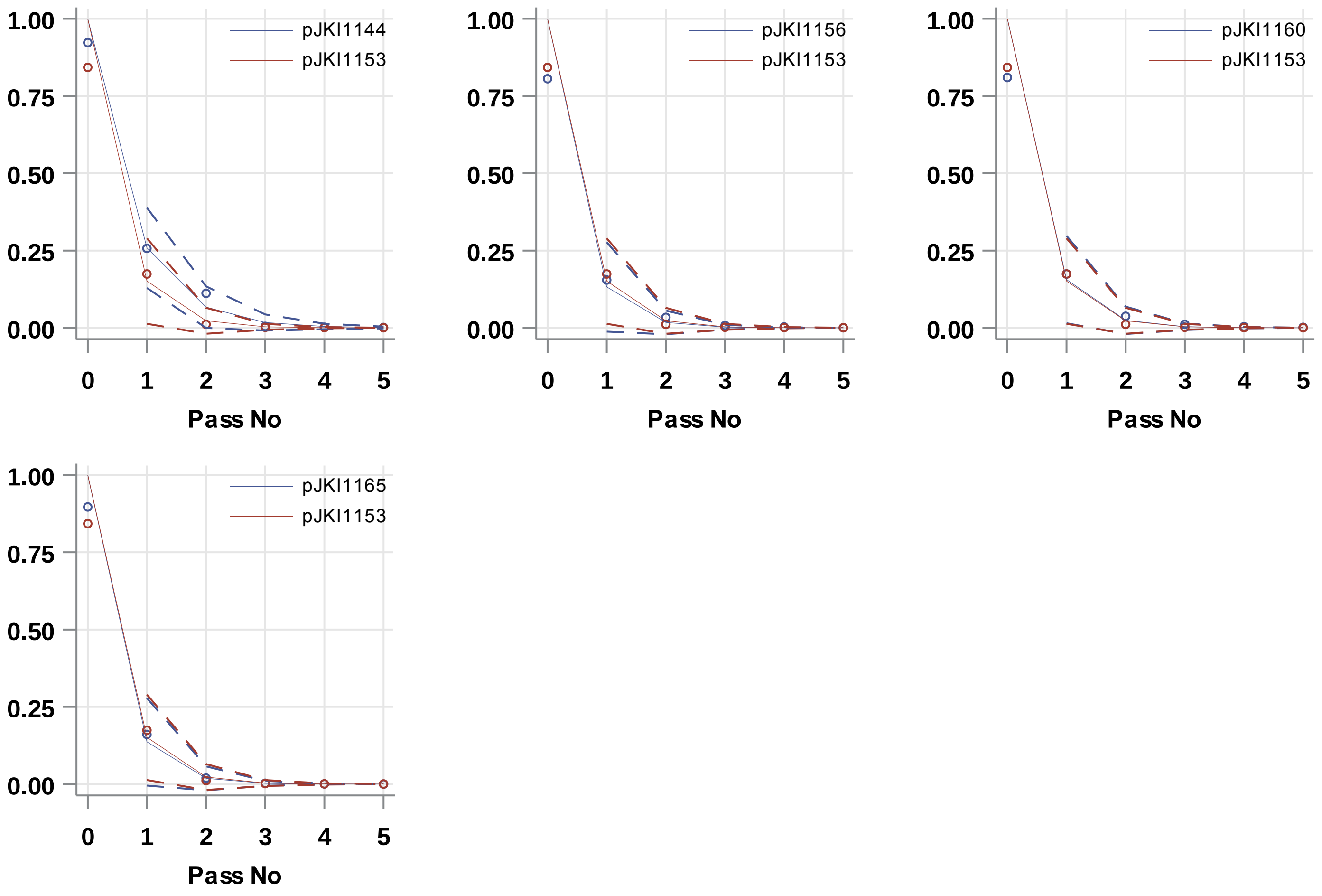

Red curves show the reference plasmid-induced $R55^{\Delta Tn}$-loss in each comparison.

Suppl. Table S1. Oligonucleotide primers used.

Name	Sequence (5'→3')[a]	References
attsgirev2	tgacagtcagaatgcggcttcg	(1)
attsgirev3	ctggagaaaatccgccccagc	(1)
attsgifor2	caacatggaaggcggcttcctg	(1)
d012seqrev	gtttaacgctctggacatctctg	this work
delNCRfor	catccacggaactgaacctctcttgtaaaaccttatatacttagagtctaCATATGAATATCCTCCTTAGTTC	this work
delNCRrev	ctttgctacttctggtgataggaatctactgaatcggtttcattatcctgGTGTAGGCTGGAGCTGCTTC	this work
DRnNCfor	aattcaaaactttcacatgtgaaagttaatcattgagatatcctcttggatgagcaaacggccaagcctgca	this work
DRnNCrev	ggcttggccgtttgctcatccaagaggatatctcaatgattaactttcacatgtgaaagttttg	this work
EcoRIDRHincIINCfor	ggcgaattcaaaactttcacatgtgaaagttaatcattgagtcaacaattattcagcatagataacagggccg	this work
Eco_parABS_Xbafor	aattcaatctttcacatgtgaaagtttt	this work
Eco_parABS_Xbarev	ctagaaaactttcacatgtgaaagattg	this work
Eco_parS2_Xbafor	aattcaaactttcacatgtgaaagtttt	this work
Eco_parS2_Xbarev	ctagaaaactttcacatgtgaaagtttg	this work
KODR1_ABfor	gtgccctatttgggcacctaggcgatttattccgacacaaaaactttcacatgcatgtgaaagtttctcattgaTCAACAGGTT GAACTGCGGATC	this work
KODR1_Crev	atctttcctgacttggatggcaatggtggttgatgtttatcaatgagaaactttcacatgcatgtgaaagttttGATTTAGGTGA CACTATAGAATAC	this work
KODR2_ABfor	aaccgaactcccaaactgtccaatttgggcagtccaaataaaactttcacatgcatgtgaaagttaatcattgaTCAACAGGT TGAACTGCGGATC	this work
KODR2_Crev	cataagtttgtactgacgtgacaaagttgccgacattattcaatgattaactttcacatgcatgtgaaagttttGATTTAGGTG ACACTATAGAATAC	this work
KOloop_NC5_Pstrev	cgctgcaggcttggccgtttgctcatcgagcaaacggagcatctctgatgc	this work
MCSfor_P	gcaattgctcgagaagcttcccgggagatctactagtctgca	this work
MCSrev_P	gactagtagatctcccgggaagcttctcgagcaattgctgca	this work
NC1_Salfor	agagtcgacatacaaacttccgaacgtcacaatgg	this work
NC3_Salfor	agagtcgacctgaacctctcttgtaaaaccttatatacttagagtc	this work
NC4_Salfor	agagtcgactaaactagcgagatttaccagtggc	this work
NC4_Salfor_ssmut	agagtcgactaaactagcgagatttaccagtggcgaagaccttggatcaaattctcgaacatgagaagccagaaattgtagcc	this work
NC4.5_Pstrev	gtacgctgcagggagcatctctgatgccttgc	this work
NC5_Pstrev	gtacgctgcaggcttggccgtttgctc	this work
NC6_Pstrev	gtacgctgcagcttctggtgatagg	this work
NC7_BgmutSpfor	accagatcttggagcatgcagaagccagaaattg	this work
NCR_ORF1_Pstrev	cacctgcagttattgatcagcgcgattttgattgtg	this work
NCR_ORF2_Ndfor	aaacatatggcaaaaacactagaccagatcttg	this work
NCR_ORF2_BPrev	aactgcagggatccctacttctggtgataggaatctactg	this work
ParaB	aattagagctctaaggaggttataacatatgtctagacagctgcggccgcaattgctcgaggatcctgcagc	this work
ParaBrev	aattgctgcaggatcctcgagcaattgcggccgcagctgtctagacatatgttataacctccttagagctct	this work
parA_Pfor	aactgcagcagcacaaaccttgtcaataagcg	this work
parA_Ncrev	ttgccatggccacctctattttggtttctcgc	this work
parA_Ndefor	ggccatatggcaaaagtaatcagcttcg	this work
parB_Ndefor	ggccatatggcattgaacaatttaaaaggcctg	this work
parB_BamHIrev	ataggatccttagccttcgacactcaggag	this work
parB_BstXIfor	gaagaaggcaagttcgtcatcc	this work
parB_XBstXrev	gggtctagacaccaccaggtcaatggtc	this work
parScore_(X)	ctagttttcacatgtgaaaa	this work
parS053Erev	gggaattcttacagttttccgtaccggttc	this work
promcatfor	aatctcgagaacttttggcgaaaatgagacg	(2)
promcatrev	attgtcgacaagctttttagcttccttagctcctgaaaatc	(2)
sgiPxisdelfor1	caccagttattgatagacctagtttatgagagacagctaccaatcctCATATGAATATCCTCCTTAGTTC	(3)
sgiPxisdelrev	gcggaattctgatgatgtccagcccccccaccgattccataccgtaattgaGTGTAGGCTGGAGCTGCTTC	(3)
003delfor	tgcaaaacaataacataaaaaaaccggcacattggccggttattaagCATATGAATATCCTCCTTAGTTC	(1)
004delrev	gtcggaataaatcgcctaggtgcccaaatagggcacttccagaggccatgGTGTAGGCTGGAGCTGCTTC	this work
005delrev	agcgaacatgtgttgaatgcccattctgccgggtctcaaaagctgcctgaGTGTAGGCTGGAGCTGCTTC	(1)
006delfor	aattagcatcgctatttgcccttttgcggcatacgcggatgtattcaCATATGAATATCCTCCTTAGTTC	(1)
007delrev	tagtttcaatcaatctgggccgcagaaaaaaaggtaaggaggactactgaGTGTAGGCTGGAGCTGCTTC	(1)
012delrev	gggttaattaaaactttttaaaaatcataagtttgtactgacgtgacaaaGTGTAGGCTGGAGCTGCTTC	this work
013delfor	gcaaatgagtacaaaccgaactcccaaactgtccaatttgggcagtcCATATGAATATCCTCCTTAGTTC	this work
018delrev	accgtcctggagagcctccgcgcccaaaaaacaccggctctgctgattaaGTGTAGGCTGGAGCTGCTTC	this work
023delfor	gccttttgggcggtgtttggatgctcaactaacttactatcggctcaCATATGAATATCCTCCTTAGTTC	this work
027delrev	atacatgttaaaatacttgtataaaattctatgttaaaaaaacgtacatgGTGTAGGCTGGAGCTGCTT	this work
044delfor	atgaactgttaagaaaatggtggtttgttgaacaagaaaataaaagctcgGTGTAGGCTGGAGCTGCTTC	this work
minidelrev	acttaccaatcacaaaacacataaagtattgattatatgaagatattactCATATGAATATCCTCCTTAGTTC	this work
pucfor24_5'FAM	FAM-cgccagggttttcccagtcacgac	this work
pucrev25_5'FAM	FAM-atttcacacaggaaacagctatgac	this work
LJ3	aacctaaccataagagaacttcc	(1)
RJ5	gatcagggagaattcgagtagag	(1)
S012promfor_Eco	ccgaattctgttgctccaataggtttcgtgtc	this work
S012promrev_P	tgctgcagcaaagttgccgacattattcaatg	this work
S013Ecofor	ttgaattctgtcggcaactttgtcacgtc	this work
S013DR_Eco	gcgaattcagtccaaataaaactttcacatg	this work
S013DR_EPAccrev	gggtcgacctgcaggaattcaaaactttcacatg	this work
S013_DRmod	ctttcacatgtaaaccgccttctcgagataatgtcggcaactttgtcacgtcag	this work
S014-15NCR_PAccfor	gggtcgacctgcagttagtgatgtaattactaagaaaagc	this work
S014_noncode_Bamrev	acggatccttagtgatgtaattactaagaaaagc	this work
S014-S015noncodeEcofor	ctgaattcttcagcatagataacagggccgtttaatac	this work
S014-S015noncodeBamrev	tgtggatccaacggccctgttatctatgctgaataattc	this work
S018Bamrev	taggatccatctacgcctatgtcatattccttaataagac	this work

[a] Restriction sites are underlined, uppercase indicates the 3' part of the KO oligos that anneals to the template plasmids pKD3 or pSG76-CS. Red letters indicate the inserted bases.

Suppl. Table S2. Relevant features of plasmids used in this study.

Name	Relevant features	References
pCP20	Ap^R, Cm^R, thermo-inducible FLP recombinase expression (λ pR::FLP), temperature-sensitive pSC101 replication system, λ cI857	(4)
pEMBL19	pMB1-based Ap^R cloning vector	(5)
pGMY6	pJKI391 derivative expression vector containing the *flhDC*$_{SGI1}$ genes under the control of P_{tac} promoter	(6)
pGMY9	pJKI391 derivative expression vector containing the *repA* gene of SGI1 under the control of P_{tac} promoter	(1)
pGMY11	pJKI88 derivative containing the 13405-16298 bp region of SGI1 including the ORFs from S013 to S018	this work
pGMY14	*Pst*I-deletion derivative of pJKI670 containing parts of SGI1 including DRL, *int*, *xis*, *oriV*, *repA* and the ORFs S016-S019.	this work
pGMY18	pJKI88 derivative containing the entire replicon (1901-3697 bp region) of SGI1	this work
pGMY20	*Bsa*BI-deletion derivative of pJKI670 containing parts of SGI1 including DRL, *int*, *xis*, *oriV*, *repA*, DR1 and the ORFs from S015 to S019. The deletion removed the C-terminal half of S013, S014 and the IGR downstream of S014.	this work
pGMY28	*Acc*I and *Pas*I-*Bam*HI-deletion derivative of pJKI670 containing parts of SGI1 including DRL, *oriV*, *repA*, S004, DR1, and the ORFs from S013 to S015. The *Acc*I deletion inactivated the *int* and *xis* genes.	this work
pGMY33	pJKI88 derivative containing P_{S004}, DR1, S013, S014 and the IGR downstream of S014, and the 5'-end of S015.	this work
pGMY34	pJKI88 derivative containing P_{S012}, DR2, S013, S014 and the IGR downstream of S014.	this work
pGMY38	pJKI88 derivative containing P_{S012}, DR2, S013, S014 and the IGR downstream of S014, and the 5'-end of S015.	this work
pGMY39	pGMY38 derivative containing a replacement mutant DR2 (DR2*)	this work
pGMY41	pGMY38 derivative containing a frameshift mutation in S013.	this work
pGMY42	pGMY38 derivative containing a frameshift mutation in S014.	this work
pGMY43	pJKI88 derivative containing DR2, S013, S014 and the IGR downstream of S014.	this work
pGMY49	pGMY38 derivative containing 4 bp insertion in the centre of the 21-bp inverted repeat in DR2 (DR2**) obtained by filling in the *Pci*I site of DR2.	this work
pGMY64	pJKI88 derivative containing the 31-bp DR2 joined to IGR (14768-15194 bp fragment of SGI1).	this work
pGMY65	Km^R Ap^R derivative of pEMBL19, containing the 31-bp DR2 joined to IGR (14768-15194 bp fragment of SGI1).	this work
pGMY66	pJKI88 derivative containing the 31-bp DR2.	this work
pGMY67	pBR322 derivative, containing the IGR (14768-15194 bp fragment of SGI1).	this work
pGMY69	Km^R Cm^S derivative of pBelobac11.	this work
pGMY72	Km^R Cm^S derivative of pBelobac11 containing the 31-bp DR2 joined to IGR (14768-15194 bp fragment of SGI1)	this work
pGMY86	Km^R *parA*$^+$*parB*$^+$*053*$^-$ derivative of pJKI1128 containing the whole *parAB053* operon (7) of R55under the control of P_{araB}. The *orf 053* is knocked out by a frameshift mutation generated by filling in its unique *Bgl*II site.	this work
pGMY91	pJKI88 derivative containing the 31-bp DR2 joined to the 3'-shortened IGR (14768-15139 bp fragment of SGI1) lacking the 18/19 bp imperfect IR motif near the 3'-end of IGR_ORF2	this work
pKD3	Cm^R, Ap^R r6kγ-based PCR template plasmid for one-step recombination gene-KO	(8)
pKD46	Ap^R, L-ara-inducible expression of λ Red recombinase, temperature-sensitive pSC101 replication system	(8)
pJKI88	Km^R p15a-based cloning vector	(9)
pJKI298	pEMBL19 derivative carrying a Km^R gene	(10)
pJKI391	pJKI88 derivative expression vector containing P_{tac} followed by a MCS and the *lacI*^{q} gene	(3)
pJKI405	Cm^RKm^S derivative of pJKI88	this work
pJKI625	p15A-based Km^R L-arabinose-inducible expression vector containing the *araC* gene and the P_{araB} followed by a MCS.	this work
pJKI670	pJKI88 derivative containing the 3' part of SGI1-Cd1 variant (11) including DRL *int*, *xis*, *oriV*, *repA*-S004, P_{S004}, DR1, S013-*mpsB*, and a truncated *mpsA*	this work
pJKI1128	pJKI625 derivative containing the whole *parAB053* operon (7) of R55 (39980-42272 bp) under the control of P_{araB}	this work
pJKI1130	pKK223-3 derivative expression vector (used as negative control)	this work
pJKI1131	pJKI88 derivative containing the 31-bp DR2 joined to the 3'-shortened IGR (14768-15071 bp of SGI1) lacking the last 23 codons of IGR_ORF2 (including the potential stem-loop motif) and the subsequent 18/19 bp imperfect IR motif	this work
pJKI1137	pJKI88 derivative containing the 31-bp DR2 joined to the 3'-shortened IGR (14768-15098 bp of SGI1) lacking the last 14 codons of IGR_ORF2 and the subsequent 18/19 bp imperfect IR motif	this work
pJKI1138	pJKI88 derivative containing the 31-bp DR2 joined to a fragment of IGR (14811-15098 bp of SGI1) lacking the downstream sequence of IGR_ORF1, the last 14 codons of IGR_ORF2, and the subsequent 18/19 bp imperfect IR motif	this work
pJKI1141	pJKI88 derivative, containing the 31-bp DR2 joined to a fragment of IGR (14926-15098 bp of SGI1) lacking the entire IGR_ORF1, the last 14 codons of IGR_ORF2, and the subsequent 18/19 bp imperfect IR motif	this work
pJKI1142	pJKI88 derivative, containing the 31-bp DR2 joined to a fragment of IGR (15007-15098 bp of SGI1) lacking the entire IGR_ORF1, the promoter region with the first seven codons and the last 14 codons of IGR_ORF2, and the subsequent 18/19 bp imperfect IR motif	this work
pJKI1144	pJKI88 derivative, containing the 31-bp DR2 joined to a fragment of IGR (14965-15098 bp of SGI1) lacking the entire IGR_ORF1, the promoter region and the last 14 codons of IGR_ORF2, and the subsequent 18/19 bp imperfect IR motif	this work
pJKI1149	pJKI88 derivative containing the full length IGR (14773-15194 bp of SGI1)	this work
pJKI1150	pEMBL19 derivative carrying a Km^R gene and the full length IGR (14773-15194 bp of SGI1)	this work
pJKI1152	pJKI1144 derivative containing the *rrnB*T1T2 terminator in front of the SD box of the 3'-truncated IGR_ORF2	this work
pJKI1153	pJKI1144 derivative containing the *rrnB*T1T2 terminator and the P_{cat} promoter in front of the SD box of the 3'-truncated IGR_ORF2	this work
pJKI1154	pJKI88 derivative, containing the 31-bp DR2 joined to a fragment of IGR (14978-15098 bp of SGI1) lacking the entire IGR_ORF1, the promoter region with the SD box, and the last 14 codons of IGR_ORF2, and the subsequent 18/19 bp imperfect IR motif	this work
pJKI1155	pJKI88 derivative, containing the 31-bp DR2 joined to a fragment of IGR (14992-15098 bp of SGI1) lacking the entire IGR_ORF1, the promoter region with the SD box and the original START (GTG-Val) and second (GCA-Ala) codons (these were replaced by GTC-Val and GAC-Asp, respectively), and the last 14 codons of IGR_ORF2 with the subsequent 18/19 bp imperfect IR motif	this work
pJKI1156	pJKI1154 derivative containing the *rrnB*T1T2 terminator and the P_{cat} promoter in front of the START codon of the 3'-truncated IGR_ORF2	this work
pJKI1157	pJKI1155 derivative containing the *rrnB*T1T2 terminator and the P_{cat} promoter in front of the 3'-truncated IGR_ORF2 lacking the START codon	this work
pJKI1158	pJKI1153 derivative containing the *rrnB*T1T2 terminator and the P_{cat} promoter in front of the SD box of the 3'-truncated IGR_ORF2, which was inactivated by a frameshift mutation generated by filling in the *Bgl*II site of IGR_ORF2	this work
pJKI1159	pJKI1153 derivative containing the *rrnB*T1T2 terminator and the P_{cat} promoter in front of the SD box of the 3'-truncated IGR_ORF2, which was inactivated by a frameshift mutation generated by a single base insertion in the IGR_ORF2	this work
pJKI1160	pJKI1153 derivative containing the *rrnB*T1T2 terminator and the P_{cat} promoter in front of the SD box of the 3'-	this work

	truncated IGR_ORF2, in which the potential stem-loop motif located in the 3'-part of IGR_ORF2 was destroyed without changing the aminoacid sequence of the putative protein expressed from the ORF	
pJKI1161	A pKK223-based Ap^R expression vector containing IGR_ORF2 under the control of P_{tac}	this work
pJKI1165	A pJKI1156 analog containing the 31-bp DR2, the *rrnB*T1T2 terminator and the P_{cat} promoter in front of the START codon of the 3'-truncated IGR_ORF2, whose 2-10 codons were replaced by synonyme codonsmodifying the secondary structure of the mRNA without changing the protein sequence	this work
pMSZ934	Ap^R, Km^R mobilizable derivative of the I-SceI producer plasmid pSTKST (12). Temperature-sensitive pSC101 replication system, Tc^R, P_{tet}::SCEI, $oriT_{RK2}$	(10)
pMSZ1166	pET16b (Novagen) derivative containing *parB* gene of R55 under the control of P_{T7}	this work
pMSZ1174	pEMBL19 derivative carrrying DR2 of SGI1 (13516-13546 bp)	this work
pMSZ1175b	Cm^R SGI1-derived replicon ($oriV_{SGI1}$+P_{tac}:$repA_{SGI1}$) harbouring the DR2 (including a *parS* site) followed by the entireIGR between ORFs S014 and S015 (14768-15194 bp) of SGI1	this work
pMSZ1177	pEMBL19 derivative harbouring the *parS* site located in the promoter region of *parAB* operon of R55 (39912-39334 bp)	this work
pMSZ1178	pEMBL19 derivative harbouring the *parS* R55_174 (representing the consensus of *parS* sites of R55 and SGI1)	this work
pMSZ1200	Cm^R SGI1-derived replicon ($oriV_{SGI1}$+P_{tac}::$repA_{SGI1}$) harbouring the DR2 (including a *parS* site)	this work
pMSZ1239	Km^R single copy plasmid derivative of pBeloBac11 containing the whole *parAB053* operon (7) of R55 (39980-42272 bp) under the control of P_{araB}	this work
pMSZ1242	Km^R single copy plasmid derivative of pBeloBac11	this work
pMSZ1248	A Cm^R, Sm^R minimal IncC replicon containing the *rep* region with *oriV* of R16a and the entire *parAB053* operon of R55 both controlled by their original promoters.	this work
pMSZ1256	pEMBL19 derivative harbouring the mutant DR2** with 4-bp insertion at the centre of the putative *parS* site	this work
pMSZ1257	pEMBL19 derivative harbouring the100% conserved and symmetric 14-bp core sequence of the putative *parS* sites of R55	this work
pMSZ1260	pJKI990 derivative β-galactosidase tester plasmid containing the 176-bp promoter region of *parAB053* operon of R55 (39807-39982 bp) cloned upstream of the *lacZ* gene, $Sm^RSp^RAp^R$	this work
pMSZ1263	Km^R $parA^-parB^+053^+$ derivative of pJKI1128 containing the whole *parAB053* operon (7) of R55 under the control of P_{araB}. The *parA* gene is knocked out by a frameshift mutation generated by insertion of a 8-mer *Xho*I-linker into the unique *Bsa*AI site.	this work
pMSZ1264	Cm^RKm^S derivative of pJKI1149 containing the full length IGR (14773-15194 bp) of SGI1	this work
pMSZ1272	Km^R $parA^+parB^-053^+$ derivative of pJKI1128 containing the whole *parAB053* operon (7) of R55under the control of P_{araB}. The *parB* gene is knocked out by a frameshift mutation generated by filling in its unique *Sty*I site.	this work
pSG76-CS	Cm^R R6K based template plasmid	(12)
R16a	IncC Type 1, tra+, $Ap^RKm^RSul^R$	(13)
$R55^{\Delta Tn}$	The Tn*6187*-deleted derivative of the IncC Type 2 plasmid R55 (=$R55^{\Delta Tn6187}$), tra+, Cm^R,$Flo^RSul^RAp^SKm^SGm^S$	(10)

Suppl. Table S3. List of bacterial strains

E. coli strains	Genotypes	References
TG1	*supE hsd*Δ*5 thi*Δ(*lac-proAB*) F'[*traD36 proAB*$^+$ *lacIq lacZ*Δ*M15*]	(14)
TG1Nal	Nal^R derivative of TG1	(11)
TG90/R55$^{\Delta Tn}$	TG1 derivative (15), *pcn B80 zad*::Tn*10* (Tc^R), R55$^{\Delta Tn}$ transconjugant, Flo^R/Cm^{R}Tc^R	this work
Tuner (DE3)	F$^-$ *ompT hsdS*$_B$(r$_B^-$ m$_B^-$) *gal dcm lacY1* (DE3)	Novagen
TG1Nal/R16a	R16a transconjugant in TG1Nal, Ap^{R}Km^{R}Sul^{R}Nal^R	(13)
TG1Nal/R55$^{\Delta Tn}$	R55$^{\Delta Tn}$ transconjugant in TG1Nal, Flo^R/Cm^{R}Nal^R	(10)
TG1Nal::SGI1-C (wt)	SGI1-C transconjugant in TG1Nal. Single copy SGI1-C is chromosomally integrated at *attB* site (*trmE*), Nal^{R}Sm^{R}Sp^{R}Sul^R	(3)
TG1Nal::SGI1-C ΔP$_{xis}$-*oriV* (Δ1)	P$_{xis}$-*oriV* deletion mutant of SGI1-C in TG1Nal strain (primers used for generation: sgiPxisdelfor1-sgiPxisdelrev), Nal^{R}Sm^{R}Sp^{R}Sul^R	(3)
TG1Nal::SGI1-C ΔS003-S004 (Δ2)	S003-S004 deletion mutant of SGI1-C in TG1Nal strain (primers used for generation: 003delfor-004delrev), Nal^{R}Sm^{R}Sp^{R}Sul^R	this work
TG1Nal::SGI1-C Δrep_region (Δ3)	S003-S005 deletion mutant of SGI1-C in TG1Nal strain (primers used for generation: 003delfor-005delrev), Nal^{R}Sm^{R}Sp^{R}Sul^R	(1)
TG1Nal::SGI1-C Δ*flhDC*$_{SGI1}$ (Δ4)	*sgaDC* (*flhDC*$_{SGI1}$) deletion mutant of SGI1-C in TG1Nal strain (primers used for generation: 006delfor-007delrev), Nal^{R}Sm^{R}Sp^{R}Sul^R	(1)
TG1Nal::SGI1-C ΔS006-S012 (Δ5)	S006-S012 deletion mutant of SGI1-C in TG1Nal strain (primers used for generation: 006delfor-012delrev), Nal^{R}Sm^{R}Sp^{R}Sul^R	this work
TG1Nal::SGI1-C ΔS013-S018 (Δ6)	S013-S018 deletion mutant of SGI1-C in TG1Nal strain (primers used for generation: 013delfor-018delrev), Nal^{R}Sm^{R}Sp^{R}Sul^R	this work
TG1Nal::SGI1-C ΔS023-S027 (Δ7)	S023-S027 deletion mutant of SGI1-C in TG1Nal strain (primers used for generation: 023delfor-027delrev), Nal^{R}Sm^{R}Sp^{R}Sul^R	this work
TG1Nal::SGI1-C ΔS044 (Δ8)	S044 deletion mutant of SGI1-C in TG1Nal strain (primers used for generation: 044delfor-minidelrev), Nal^R Sm^R Sp^R Sul^R	this work
TG1Nal::*attP*$_{SGI1}$	TG1Nal::[miniTn*10*::*attP*$_{SGI1}$-Km^R] containing the junction fragment of circular SGI1 (*attP*: 42126-42433::1-496 bp), Nal^{R}Km^R	(1)
TG1Nal::SGI1-C KODR1	DR1 KO mutant SGI1-C in TG1Nal strain carrying CATG insertion at the *Pci*I cleavage site of *parS* located in the DR1 repeat (primers used for generation:: KODR1_ABfor-KODR1_Crev), Nal^R Sm^R Sp^R Su^R	this work
TG1Nal::SGI1-C KODR2	DR2 KO mutant SGI1-C in TG1Nal strain carrying CATG insertion at the *Pci*I cleavage site of *parS* located in the DR2 repeat (primers used for generation:: KODR2_ABfor-KODR2_Crev), Nal^{R}Sm^{R}Sp^{R}Sul^R	this work
TG1Nal::SGI1-C KODR1+KODR2	Double KO mutant SGI1-C in TG1Nal strain carrying CATG insertion at the *Pci*I cleavage sites of both *parS* sites located in the DR1 and DR2 repeats, Nal^{R}Sm^{R}Sp^{R}Sul^R	this work
TG1Nal::SGI1-C Δ*sci*	IGR deletion mutant SGI1-C in TG1Nal strain lacking the IGR_ORF2 (*sci* gene) in the intergenic region between S014 and S015 (primers used for generation: delNCRfor-delNCRrev), Nal^{R}Sm^{R}Sp^{R}Sul^R	this work
TG1Nal::SGI1-C Δ*sci*+KODR1+KODR2	Triple KO mutant SGI1-C in TG1Nal strain lacking the IGR_ORF2 (*sci* gene) in the intergenic region between S014 and S015 and carrying CATG insertion at the *Pci*I cleavage site of both *parS* sites located in the DR1 and DR2 repeats, Nal^{R}Sm^{R}Sp^{R}Sul^R	this work

Suppl. Table S4

SGI1-C	total Nal	colony counts R55+ NalCm	SGI1+ NalSm/Sp	R55+SGI1+ NalCmSm/Sp	R55+SGI1- NalCm-NalCmSmSp	R55-SGI1+ NalSmSp-NalCmSmSp	SGI1+R55 mean	st.dev.	SGI1 mean	st.dev.	R55 mean	st.dev.		none mean	st.dev.
DR1 KO															
1/1	652	652	17	17	635	0	2,6%		0,0%		97,4%		100,0%	0,0%	
1/2	305	300	39	34	266	5	11,1%		1,6%		87,2%		100,0%	0,0%	
1/3	340	340	15	15	325	0	4,4%		0,0%		95,6%		100,0%	0,0%	
1/4	189	157	22	12	145	10	6,3%		5,3%		76,7%		88,4%	11,6%	
							6,1%	3,2%	1,7%	2,2%	89,2%	8,2%	97,1%	2,9%	5,0%
DR2 KO															
2/1	853	95	777	48	47	729	5,6%		85,5%		5,5%		96,6%	3,4%	
2/2	314	22	284	11	11	273	3,5%		86,9%		3,5%		93,9%	6,1%	
2/3	653	83	511	40	43	471	6,1%		72,1%		6,6%		84,8%	15,2%	
2/5	979	21	954	7	14	947	0,7%		96,7%		1,4%		98,9%	1,1%	
							4,0%	2,1%	85,3%	8,8%	4,3%	2,0%	93,6%	6,4%	5,3%
Δ*sci*															
3/1	300	300	10	10	290	0	3,3%		0,0%		96,7%		100,0%	0,0%	
3/4	530	530	56	56	474	0	10,6%		0,0%		89,4%		100,0%	0,0%	
3/5	267	267	28	28	239	0	10,5%		0,0%		89,5%		100,0%	0,0%	
3/6	83	83	1	1	82	0	1,2%		0,0%		98,8%		100,0%	0,0%	
							6,4%	4,2%	0,0%	0,0%	93,6%	4,2%	100,0%	0,0%	0,0%
DR1+DR2 KO															
4/3	157	157	50	50	107	0	31,8%		0,0%		68,2%		100,0%	0,0%	
4/4	214	214	61	61	153	0	28,5%		0,0%		71,5%		100,0%	0,0%	
4/5	281	281	71	71	210	0	25,3%		0,0%		74,7%		100,0%	0,0%	
4/6	300	300	85	85	215	0	28,3%		0,0%		71,7%		100,0%	0,0%	
							28,5%	2,3%	0,0%	0,0%	71,5%	2,3%	100,0%	0,0%	0,0%
DR1+DR2 KO + Δ*sci*															
5/1	420	420	107	107	313	0	25,5%		0,0%		74,5%		100,0%	0,0%	
5/3	170	170	30	30	140	0	17,6%		0,0%		82,4%		100,0%	0,0%	
5/5	91	91	22	22	69	0	24,2%		0,0%		75,8%		100,0%	0,0%	
5/6	362	362	55	55	307	0	15,2%		0,0%		84,8%		100,0%	0,0%	
					0		20,6%	4,3%	0,0%	0,0%	79,4%	4,3%	100,0%	0,0%	0,0%
wt															
6/1	218	1	208	0	1	208	0,0%		95,4%		0,5%		95,9%	4,1%	
6/2	769	0	732	0	0	732	0,0%		95,2%		0,0%		95,2%	4,8%	
6/4	200	0	199	0	0	199	0,0%		99,5%		0,0%		99,5%	0,5%	
6/5	858	12	847	1	11	846	0,1%		98,6%		1,3%		100,0%	0,0%	
							0,0%	0,1%	97,2%	1,9%	0,4%	0,5%	97,6%	2,4%	2,1%

Supplementary Text S1.

SGI1 fragments were amplified using the genomic DNA of TG1Nal::SGI1-C strain (3) as template DNA unless otherwise specified.

pGMY11: The 13405-16298 bp region of SGI1 includinging ORFs from S013 to S018 was amplified from pJKI670 template DNA with primers S013Ecofor and S018Bamrev. The amplicon was digested with *Eco*RI and *Bam*HI and cloned into the *Eco*RI-*Bam*HI site of pJKI88 (9).

pGMY14: Plasmid pJKI670 was digested by *Pst*I and recircularized by self-ligation.

pGMY18: The upstream region of S004 was completed in pMSZ1008 contaning the SGI1-derived replicon (1). The 3288-3697 bp region of SGI1 was amplified with primers sgiseq1for - sgiseq1rev (1), and the amplicon was digested with *Pst*I and ligated into the *Pst*I site of pMSZ1008 resulting in pMSZ1015, a functional SGI1-based minimal replicon. Then, pMSZ1015 was linearized with *Xba*I and ligated into the *Xba*I site of pJKI88 resulting in pGMY17. The Cm^R (*cat*) gene was deleted from pGMY17 by *Not*I-*Bam*HI digestion and self-ligation after blunting the incompatible ends with Klenow polymerase.

pGMY20: Deletion was generated in pJKI670 by *Bsa*BI digestion and self-ligation.

pGMY28:First, pJKI670 was digested with *Acc*I having 4 cleavage sites, but only those sites produce compatible ends, which are present in *int* and *xis*, thus self-ligation and antibiotic selection for the transformants provided the *Acc*I-deletion derivative, pGMY15. Then, pGMY15 was digested with *Pas*I and *Bam*HI and recircularized after blunting the incompatible ends with Klenow polymerase.

pGMY33: The *Pst*I fragment of pJKI670 was ligated into the *Pst*I site of pJKI88.

pGMY34: The 13405-15194 bp region of SGI1 including the ORFs S013-S014 was amplified from pJKI670 with primers S013Ecofor and S014-S015noncodeBamrev. The amplicon was digested with *Eco*RI and *Bam*HI and cloned into the *Eco*RI-*Bam*HI site of pJKI88, resulting in pGMY12. Then, the fragment containing the upstream region of S012 was amplified from SGI1-C with primers S012promforEco and d012seqrev. The amplicon was digested with *Eco*RI and *Pac*I and cloned into the *Eco*RI-*Pac*I site of pGMY12.

pGMY38: The *Eco*RI-*Mfe*I fragment of pGMY34 was ligated into the *Eco*RI-*Mfe*I site of pGMY33.

pGMY39: The DR2 sequence of SGI1 was amplified from pGMY38 template using primers S013_DRmod and d012seqrev, which led to a replacement of the 3' 13 bp of DR2 (DR2*). The amplicon was digested with *Eco*RI and *Pac*I and cloned into the *Eco*RI-*Pac*I site of pGMY38.

pGMY41: A 8 bp *Xho*I-linker (CCTCGAGG) was cloned into the *Hinc*II site of pGMY38.

pGMY42: A 8 bp *Xho*I-linker (CCTCGAGG) was cloned into the *Bsa*AI site of pGMY38.

pGMY43: The 1361-15194 bp region of SGI1 containing DR2, S013-S014 and the IGR separating ORFs S014 and S015 was amplified with primers S013DR_Eco and S014_noncode_Bamrev. The amplicon was digested with *Eco*RI and *Bam*HI and cloned into the *Eco*RI-*Bam*HI site of pJKI88.

pGMY49: The plasmid pGMY38 was digested with *Pci*I and religated after filling in the sticky ends using Klenow polymerase.

pGMY64: The IGR was amplified with primers EcoRIDRHincIINCfor (containing the DR2 sequence) and S014_noncode_Bamrev. The amplicon was digested with *Eco*RI-*Bam*HI and cloned into the *Eco*RI-*Bam*HI site of pJKI88.

pGMY65: The *Eco*RI-*Sal*I fragment of pGMY64 was inserted into the *Eco*RI-*Sal*I site of pJKI298 (10).

pGMY66: The IGR was deleted from pGMY64 by *Hinc*II digestion and religation.

pGMY67: The *Hinc*II fragment (IGR) of pGMY64 was ligated into the *Eco*RV site of pBR322 (16).

pGMY69: The Km^R cassette of pUC4K was cloned into the *Eco*RI site of pBelobac11 (New England Biolabs).

pGMY72: The *Eco*RI-*Not*I fragment of pGMY65 was inserted into the *Eco*RI-*Not*I site of pBelobac11.

pGMY74: The DRnNCfor and DRnNCrev primers containing the DR2 sequence (13371-13401 bp of SGI1) joined to an inverted repeat motif in the IGR (15076-15097 bp of SGI1) were annealed and cloned into the *Eco*RI-*Pst*I site of pEMBL19.

pGMY86: The *orf 053* was knocked out in pJKI1128 by self-ligation of blunted ends after *Bgl*II digestion and filling in the sticky ends using Klenow polymerase.

pGMY91: The 14768-15139 bp region of SGI1 was amplified with primers EcoRIDRHincIINCfor and NC6_Pstrev. The amplicon was digested with *Eco*RI-*Pst*I and cloned into the *Eco*RI-*Pst*I site of pJKI88.

pJKI405: The Cm^R (*cat*) cassette was cut out with *Sal*I from pAW302 (17) and ligated into the *Xho*I-*Sal*I-digested pJKI88.

pJKI625: The *EcoR*I-*Cla*I fagment of pKD46 (Datsenko and Wanner, 2000) harbouring the *araC* gene with the P_{araB} promoter was inserted into the *EcoR*I-*Sal*I site of pJKI88 in two steps, leading to pJKI624. Then, the ParaB and ParaBrev primers were annealed and cloned into the *EcoR*I site of pJKI624.

pJKI670: Deletion was generated in pJKI669 (10) by NcoI digestion and religation.

pJKI1128: *parB* and ORF R55_053 were amplified with primers parB_Ndefor and parS053Erev and the *Stu*I-*EcoR*I fragment of the amplicon was cloned into the *EcoR*V-*EcoR*I site of pBluescriptII SK resulting in pMSZ1201. *parA* and the 5'-part of *parB* (39979-41056) were amplified from R55 with primers parANdefor and parBXBstXrev, the amplicon was digested with *Nde*I-*Xba*I and cloned into the *Nde*I-*Xba*I site of pJKI391

resulting in pMSZ1202. Then, the *Sty*I-*Xba*I fragment of pMSZ1201 was cloned into the *Sty*I-*Xba*I site of pMSZ1202 resulting in pMSZ1211, in which the whole three-gene *par* operon of R55 was placed under the control of P_{tac}. Finally the *Nde*I-*Sal*I fragment of pMSZ1211 was cloned into the *Nde*I-*Xho*I site of pJKI625 placing the *par* operon under the control of P_{araB}.

pJKI1130: The ORF of IS*30* transposase has been deleted from pJKI397, a pKK223-3 derivative expression vector that is analogous to pJKI132 (18), but contains an *Nde*I and a *Xho*I site overlapping the start codon of the transposase and the *Nde*I site in the vector backbone has been eliminated by filling in. The *Nde*I-*Pst*I digested pJKI397 was self-ligated after blunting the incompatible ends.

pJKI1131: The 14768-15071 bp region of SGI1 was amplified with primers EcoRIDRHincIINCfor and NC4.5_Pstrev and the *Eco*RI-*Pst*I-digested amplicon was cloned into the *Eco*RI-*Pst*I site of pJKI88.

pJKI1137: The 14768-15098 bp region of SGI1 was amplified with primers EcoRIDRHincIINCfor and NC5_Pstrev and the *Eco*RI-*Pst*I-digested amplicon was cloned into the *Eco*RI-*Pst*I site of pJKI88.

pJKI1138: The 14811-15098 bp region of SGI1 was amplified with primers NCR_ORF1_Pstrev and NC5_Pstrev, then the *Pst*I-digested amplicon was ligated into the *Pst*I site of pGMY66.

pJKI1139: The 14850-15098 bp region of SGI1 was amplified with primers NC1_Salfor and NC5_Pstrev. The amplicon was digested with *Sal*I and *Pst*I and cloned into the *Sal*I-*Pst*I site of pGMY66.

pJKI1141: The 14926-15098 bp region of SGI1 was amplified with primers NC3_Salfor and NC5_Pstrev. The amplicon was digested with *Sal*I and *Pst*I and cloned into the *Sal*I-*Pst*I site of pGMY66.

pJKI1142: The *Hinc*II-*Bgl*II fragment of pJKI1139 was deleted by the appropriate digestion and recircularization after filling in the protruding end with Klenow polymerase.

pJKI1144: The 14965-15098 bp region of SGI1 was amplified with primers NC4_Salfor and NC5_Pstrev. The amplicon was digested with *Sal*I and *Pst*I and cloned into the *SalI*I-*Pst*I site of pGMY66.

pJKI1149: The 14773-15194 bp region of SGI1 (the entire IGR separating the ORFs S014 and S015) was amplified with primers S014-S015noncodeEcofor and S014_noncode_Bamrev. The amplicon was digested with *Eco*RI and *Bam*HI and cloned into the *Eco*RI-*Bam*HI site of pJKI88.

pJKI1150: The 14773-15194 bp fragment of SGI1 was amplified with primers S014-S015noncodeEcofor and S014_noncode_Bamrev. The amplicon was digested with *Eco*RI and *Bam*HI and cloned into the *Eco*RI-*Bam*HI site of pEMBL19, resulting in pJKI1148. Then, the Km^R cassette was cut out with *Xba*I-*Sph*I digestion from pJKI298 and inserted into the *Xba*I-*Sph*I site of pJKI1148.

pJKI1151: The P_{cat} promoter region was amlified from pAW302 with primers promcatfor and promcatrev. The amplicon was digested with *Xho*I and *Sal*I and ligated into the *Sal*I site of pJKI988 (3).

pJKI1152:Th *rrnB*T1T2 terminator cassette was cut out from pJKI988 (3) with *Xho*I-*Sal*I and ligated into the *Sal*I site of pJKI1144.

pJKI1153: The *rrnB*T1T2-P_{cat} cassette was cut out with *Xho*I and *Sal*I from pJKI1151 and ligated into the *Sal*I site of pJKI1144.

pJKI1154: The 14965-15099 bp region of SGI1 was amplified with primers NC5_Salfor and NC5_Pstrev. The amplicon was digested with *Sal*I and *Pst*I and cloned into the *SalI*I-*Pst*I site of pGMY66.

pJKI1155: The 14992-15099 bp region of SGI1 was amplified with primers NC6_Salfor and NC5_Pstrev. The amplicon was digested with *Sal*I and *Pst*I and cloned into the *SalI*I-*Pst*I site of pGMY66.

pJKI1156: The *rrnB*T1T2-P_{cat} cassette was cut out with *Xho*I and *Sal*I from pJKI1151 and ligated into the *Sal*I site of pJKI1154.

pJKI1157: The *rrnB*T1T2-P_{cat} cassette was cut out from pJKI1151 with *Xho*I and *Sal*I digestion and ligated into the *Sal*I site of pJKI1155.

pJKI1158: pJKI1153 was digested with *Bgl*II and self-ligated after filling in the sticky ends.

pJKI1159: The 15003-15098 bp region of SGI1 was amplified with primers NC7_BgmutSpfor and NC5_Pstrev. The amplicon was digested with *Bgl*II-*Pst*I and ligated into the *Bgl*II-*Pst*I site of pJKI1153.

pJKI1160: The DR2-*rrnB*T1T2-P_{cat}-IGR_ORF2 region was amplified from pJKI1153 template with primers S013DR_EPAccrev and KOloop_NC5_Pstrev. The amplicon was digested with *Eco*RI-*Pst*I and ligated into the *Eco*RI-*Pst*I site of pJKI88.

pJKI1161: The IGR_ORF2 of SGI1 was amplified with primers NCR_ORF2_Ndfor and NCR_ORF2_BPrev. The amplicon was digested with *Nde*I-*Pst*I and ligated into the *Nde*I-*Pst*I site of pJKI397 (see pJKI1130).

pJKI1165: The 14965-15098 bp region of SGI1-C was amplified with primers NC4_Salfor_ssmut and NC5_Pstrev. The *Sal*I-*Pst*I-digested amplicon was ligated into the *Sal*I-*Pst*I site of pGMY66, leading to pJKI1164. Then, the *rrnB*T1T2-P_{cat} cassette was cut out from pJKI1151 with *Xho*I and *Sal*I and ligated into the *Sal*I site of pJKI1164.

pMSZ1166: The *parB* gene of R55 (40769-41950 bp) was amplified with primers parB_Ndefor and parB_BamHIrev. The amplicon was digested with *Nde*I-*BamH*I and ligated into the *Nde*I-*BamH*I site of pET-16b (Novagen).

pMSZ1174: The inverted repeat was deleted from pGMY74 by *EcoR*V-*Hind*III digestion and religation after end repair using the NEBNext® End Repair Module.

pMSZ1175b: The fragment containing the DR2 repeat (13371-13401 bp) and the IGR between ORFs S014 and S015 (14768-15195 bp) was amplified from pGMY64 template with primers S014-15NCR_PAccfor - S013DR_EPAccrev. The amplicon was digested with *Acc*I and cloned into the *Cla*I site of pMSZ1012 (1).

pMSZ1177: The Eco_parABS_Xbafor and Eco_parABS_Xbarev oligonucleotides including the putative *parS* site located in the promoter region of *parAB053* operon of R55 (39912-39334 bp) were annealed and cloned into the *Eco*RI-*Xba*I site of pEMBL19.

pMSZ1178: The Eco_parS2_Xbafor and Eco_parS2_Xbarev oligonucleotides including the putative *parS* site located upstream of ORF R55_174 (166296-166317 bp) were annealed and cloned into the *Eco*RI-*Xba*I site of pEMBL19.

pMSZ1200: The *Pst*I fragment of pMSZ1175b containing the DR2-IGR cassette was replaced with the annealed MCSfor_P and MCSrev_P primers resulting in a new SGI1-derived basic replicon, pMSZ1199. The DR2 was cut out from pGMY74 with *Eco*RI-*Eco*RV and cloned into the *Mfe*I-*Sma*I site of pMSZ1199.

pMSZ1239: The *Xmn*I-*Xba*I fragment of pJKI1128 containing the Km^R gene, *araC* and the P_{araB}::*parAB053* operon of R55 was ligated into the *Nru*I-digested pBeloBac11 after filling in the protruding end generated by *Xba*I.

pMSZ1242: The P_{araB}::*parAB053* operon was deleted from pMSZ1239 with *Bam*HI and the remaining vector backbone was self-ligated.

pMSZ1248: The minimal replicon was constructed via several consecutive steps as follows: The *rep* region with *oriV* was cut out by *Xmn*I-*Bse*JI digestion from R16a and ligated into the *Hinc*II-digested pSG76-CS resulting in pMSZ1162. Then, the annealed oligos MCSfor_P and MCSrev_P were cloned into the unique *Pst*I site of pMSZ1162 leading to pMSZ1179. The R6Kγ replication origin of pSG76-CS was deleted from pMSZ1179 by I-*Sce*I-*Spe*I digestion followed by self-ligation after blunting the incompatible ends using the NEBNext® End Repair Module, which resulted in pMSZ1185. The *rrnB*T1T2 terminator cassette was cut out from pJKI988 (3) with *Sal*I-*Xho*I and ligated into the *Xho*I site of pJKI626, resulting in pAHG5, from where the terminator cassette was cut out with *Pst*I-*Mfe*I and ligated into the *Pst*I-*Mfe*I site of pMSZ1185, resulting in pMSZ1194. The 2709 bp *Pst*I-*Bgl*II fragment containing the *parAB* genes of R55 with its own promoter region was cloned into the *Pst*I-*Bam*HI site of pJKI691 (3), leading to pJKI822. The 3' part of *parB* and the entire orf *053* was amplified from R55 with primers parB_BstXIfor and parS053Erev, the amplicon was digested with *Bst*XI-*Eco*RI and ligated into the *Bst*XI-*Eco*RI-digested pJKI822, resulting in pMSZ1172, which contained the entire *parAB053* operon. The operon then was cut out from pMSZ1172 with *Eco*RI-*Hind*III and ligated into *Mfe*I-*Hind*III site of pMSZ1194, leading to pMSZ1198. Finally, the Sm^R cassette of pHP45Ω (19) bracketed by transcription and translation terminators was cut out with *Sma*I and inserted into the *Sma*I site of pMSZ1198, resulting in the single copy minimal IncC replicon, pMSZ1248.

pMSZ1256: The insertion mutant DR2** was amplified from pGMY49 with primers S013DR_Eco and S012promrev_P. The amplicon was digested with *Eco*RI-*Pst*I and cloned into the *Eco*RI-*Pst*I site of pEMBL19.

pMSZ1257: The parScore_(X) primer containing the and symmetric 14-bp 100% conserved core sequence of the putative *parS* sites was self-annealed and cloned into the *Xba*I site of pEMBL19.

pMSZ1260: The promoter region of *parAB053* operon (39807-39982 bp) was amplified from R55 with primers parA_Pfor and parA_Ncrev. The amplicon was digested with *Nco*I-*Pst*I and ligated into the *Nco*I-*Pst*I site of pJKI990 (3).

pMSZ1263: The *parA* gene was knocked out in pJKI1128 by ligation of a 8-mer *Xho*I-linker into the unique *Bsa*AI site.

pMSZ1264: The Cm^R (*cat*) cassette was cut out from pAW302 (17) with *Sal*I and ligated into the *Xho*I-*Sal*I-digested pJKI1149

pMSZ1272: The *parB* gene was knocked out in pJKI1128 by self-ligation of blunted ends after *Sty*I digestion and filling in the sticky ends using Klenow polymerase.

Supplementary references

1. Szabó,M., Murányi,G. and Kiss,J. (2021) IncC helper dependent plasmid-like replication of Salmonella Genomic Island 1. *Nucleic Acids Res.*, **49**, 832–846.

2. Nagy,I., Szabó,M., Hegyi,A. and Kiss,J. (2021) Salmonella Genomic Island 1 requires a self- encoded small RNA for mobilization. *Mol. Microbiol.*, **116**, 1533–1551.

3. Kiss,J., Papp,P.P., Szabó,M., Farkas,T., Murányi,G., Szakállas,E. and Olasz,F. (2015) The master regulator of IncA/C plasmids is recognized by the Salmonella Genomic island SGI1 as a signal for excision and conjugal transfer. *Nucleic Acids Res.*, **43**, 8735–8745.

4. Cherepanov,P.P. and Wackernagel,W. (1995) Gene disruption in Escherichia coli: TcR and KmR cassettes with the option of Flp-catalyzed excision of the antibiotic-resistance determinant. *Gene*, **158**, 9–14.

5. Dente,L., Cesareni,G. and Cortese,R. (1983) pEMBL: A new family of single stranded plasmids. *Nucleic Acids Res.*, **11**, 1645–1655.

6. Murányi,G., Szabó,M., Olasz,F. and Kiss,J. (2016) Determination and Analysis of the Putative AcaCD-Responsive Promoters of Salmonella Genomic Island 1. *PLoS One*, **11**, e0164561.

7. Hancock,S.J., Phan,M.-D., Peters,K.M., Forde,B.M., Chong,T.M., Yin,W.-F., Chan,K.-G., Paterson,D.L., Walsh,T.R., Beatson,S.A., *et al.*

(2017) Identification of IncA/C Plasmid Replication and Maintenance Genes and Development of a Plasmid Multilocus Sequence Typing Scheme. *Antimicrob. Agents Chemother.*, **61**, e01740-16.

8. Datsenko,K.A. and Wanner,B.L. (2000) One-step inactivation of chromosomal genes in Escherichia coli K-12 using PCR products. *Proc. Natl. Acad. Sci.*, **97**, 6640–6645.
9. Kiss,J. and Olasz,F. (1999) Formation and transposition of the covalently closed IS 30 circle : the relation between tandem dimers and monomeric circles. *Mol. Microbiol.*, **34**, 37–52.
10. Kiss,J., Szabó,M., Hegyi,A., Douard,G., Praud,K., Nagy,I., Olasz,F., Cloeckaert,A. and Doublet,B. (2019) Identification and Characterization of oriT and Two Mobilization Genes Required for Conjugative Transfer of Salmonella Genomic Island 1. *Front. Microbiol.*, **10**, 1–16.
11. Kiss,J., Nagy,B. and Olasz,F. (2012) Stability, entrapment and variant formation of Salmonella genomic island 1. *PLoS One*, **7**, e32497.
12. Kolisnychenko,V., Plunkett,G., Herring,C.D., Fehér,T., Pósfai,J., Blattner,F.R. and Pósfai,G. (2002) Engineering a reduced Escherichia coli genome. *Genome Res.*, **12**, 640–7.
13. Szabó,M., Nagy,T., Wilk,T., Farkas,T., Hegyi,A., Olasz,F. and Kiss,J. (2016) Characterization of Two Multidrug-Resistant IncA/C Plasmids from the 1960s by Using the MinION Sequencer Device. *Antimicrob. Agents Chemother.*, **60**, 6780–6786.
14. Gibson,T.J. (1984) Studies on the Epstein-Barr virus genome. Thesis.
15. Gonzy-Treboul,G., Karmazyn-Campelli,C. and Stragier,P. (1992) Developmental regulation of transcription of the Bacillus subtilis ftsAZ operon. *J. Mol. Biol.*, **224**, 967–979.
16. Bolivar,F., Rodriguez,R.L., Greene,P.J., Betlach,M.C., Heyneker,H.L., Boyer,H.W., Crosa,J.H. and Falkow,S. (1977) Construction and characterization of new cloning vehicles. II. A multipurpose cloning system. *Gene*, **2**, 95–113.
17. Stalder,R. and Arber,W. (1989) Characterization of in vitro constructed IS30-flanked transposons. *Gene*, **76**, 187–93.
18. Farkas,T., Kiss,J. and Olasz,F. (1996) The construction and characterization of an effective transpositional system based on IS30. *FEBS Lett.*, **390**, 53–58.
19. Prentki,P. and Krisch,H.M. (1984) In vitro insertional mutagenesis with a selectable DNA fragment. *Gene*, **29**, 303–313.
